# Designing the framework structure of noble-metal based nanoalloy catalysts driving redox electrocatalysis†

**DOI:** 10.1039/d4sc03142c

**Published:** 2024-06-26

**Authors:** Guanzhen Chen, Jie Zhang, Wen Chen, Ruihu Lu, Chao Ma, Ziyun Wang, Yunhu Han

**Affiliations:** a Institute of Flexible Electronics (IFE) and Frontiers Science Center for Flexible Electronics, Northwestern Polytechnical University Xi'an 710072 China iamyhhan@nwpu.edu.cn; b School of Chemical Sciences, The University of Auckland Auckland 1010 New Zealand ziyun.wang@auckland.ac.nz; c Department of Chemistry, Tsinghua University Beijing 100084 China

## Abstract

Noble metal-based nanoalloys (NAs) with different entropies have great potential in the field of energy and catalysis. However, it is still very difficult for the reported synthesis strategies to achieve the universal synthesis of small-sized alloys with controllable morphology. Here we develop a general synthesis strategy that combined cation exchange and spatial confinement (CESC). We used this method to construct a library with 21 NAs having low to high entropies. Importantly, we also demonstrate that the method can controllably achieve framing of almost all the NAs obtained, which can be realized by adjusting the amount of non-precious metals, despite the differences in the number of elements. Moreover, the CESC method showed outstanding ability to suppress the sintering of NAs and regulate the particle size of NAs. In the NA library, the framed PtCu/HCN as a redox electrocatalyst shows superior properties. For the methanol oxidation reaction (MOR), the specific and mass activities (7.02 mA cm^−2^ and 2.81 A mg_Pt_^−1^) of PtCu/HCN show 28.1- and 13.4-fold enhancement compared to those of commercial Pt/C, and the peak current density is only attenuated by 5% after 50k seconds of chronoamperometry. For the hydrogen evolution reaction (HER), it can operate at ultralow overpotential (23.5 mV and 10 mA cm^−2^) for 150 h, far exceeding most of the reported catalysts. Moreover, the catalyst is capable of long-term hydrogen evolution at ultra-low overpotentials. Our work offers opportunities for synthesizing framed superfine noble metal-based NAs with different entropies.

## Abstract 2

We develop a robust and general strategy, for successful construction of a library of 21 nanoalloy materials with different entropies. Amazingly, the framing of nanoalloys can be achieved by controlling the feeding amount of non-noble metals.

## Graphical abstract

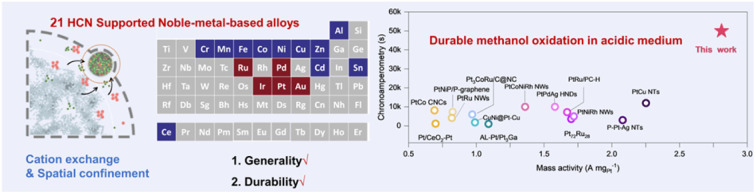

## 1 Introduction

Nanoalloys (NAs) with different entropies are widely used in catalysis, electronics, energy storage and conversion due to their tunable adsorption energy, atomic efficiency and local environment.^1–10^ When several metals are randomly dissolved in an equal atomic ratio, the configurational entropy of the alloy system reaches the maximum value. The alloy systems are classified based on the configurational entropy: if the configurational entropy is less than 1*R*, it is a low-entropy alloy (LEA), consisting of one or two elements. The configurational entropy of a medium-entropy alloy (MEA) is between 1*R* and 1.5*R*, and the alloy is composed of three or four elements. For a high-entropy alloy (HEA), the configurational entropy is greater than 1.5*R* and it consists of at least five elements.^11,12^ NAs with different entropies usually exhibit significantly different physical, chemical, and mechanical properties, and the synthesis methods of these alloys are also significantly different. It is well known that the alloying miscibility process proceeds along with a limited number of similar compositions and is seriously affected by differences in, for example, atomic radius and electronegativity,^13,14^ leading to difficulties in the general fabrication of NAs with different entropies. In addition, much effort has been devoted to the design and construction of noble metal-based NAs with open-framework structures, such as nano-cages and nano-frameworks, which facilitate the exposure of noble metal sites inside the alloys, thus improving the intrinsic activity of the catalysts and enhancing the noble metal atom utilization.^15–18^ However, the controlled and general preparation of the above-mentioned NAs with open-framework structures is complicated and difficult, even less so for multi-element alloys.

Although a lot of effort has been devoted to the development of synthesis methods for NAs, the reported approaches still suffer from insurmountable drawbacks. The wet-chemical methods inevitably use a lot of difficult-to-remove surfactants in the NA synthesis.^19–22^ Furthermore, the general synthesis of NAs with different entropies is difficult. The just-reported sulfur-anchoring method realizes the preparation of binary to senary Pt-based NAs through multiple calcinations.^23^ Recently, the carbothermal shock technique has achieved the creation of NAs containing multiple metal components at a very high temperature and extremely fast ramping/cooling rate.^24^ The laser scanning ablation method can be used to obtain various HEAs with a size of tens of nanometers on different substrates.^25^ Both the methods require rigorous conditions and special equipment. Therefore, the development of a simple general technique to fabricate small-sized NAs with different entropies, while being able to construct noble metal-based NAs with desirable structures such as nanocages or nano-framework structures, is crucial and attractive for the broad applications of the NA materials.

Herein, we introduce a robust and universal strategy combining cation exchange and spatial confinement (CESC) for synthesizing nanoalloys ranging from LEAs to HEAs. The novelty of this strategy is introducing a cation exchange process to achieve the universal preparation of nanoalloys with different entropies. Moreover, we use a spatial confinement method to control the size of nanoalloys and drive the alloying metal combinations. Specifically, the exchange of NH_4_^+^-rich SiO_2_ spheres with various metal ions is the key to ensuring the universality of the strategy, and the elastic space created by the coating polymer to drive the uniform alloying of each metal ion is a necessary supplement for the smooth implementation of the strategy. A library containing 21 nanoalloys ranging from LEAs to HEAs has been constructed successfully by the CESC strategy, including 9 LEAs, 4 medium-entropy nanoalloys (MEAs) and 8 HEAs. Impressively, nanoalloys with nano-frame structures can also be fabricated by the CESC strategy. Most of the synthesized nanoalloys in our library show great potential: For example, we observed superior electrocatalytic performances for the MOR and HER of the LEA PtM/HCN (M = Cu, Co, Ni and Fe), especially PtCu/HCN. For the MOR, the specific and mass activities (7.02 mA cm^−2^ and 2.81 A mg_Pt_^−1^) of PtCu/HCN are 28.1- and 13.4-fold enhancements compared to those of commercial Pt/C, and the peak current density is only attenuated by 5% after 50k seconds of chronoamperometry (an order of magnitude enhancement compared to the catalysts reported; CA). It can operate at ultralow overpotential (23.5 mV and 10 mA cm^−2^) for 150 h for the HER, far beyond commercial Pt/C. Density functional theory (DFT) calculations reveal that Cu atoms lower the d-band center of Pt sites and thus reduce carbonaceous and H adsorption, enhancing the activity of the MOR and HER. This CESC strategy provides a promising method for noble metal-based nanoalloys with different entropies.

## 2 Results and discussion

### 2.1 Material synthesis and characterization

For the fabrication of nanoalloys, we developed a robust CESC strategy that introduces cation exchange to realize the generality of nanoalloys ranging from LEAs to HEAs, and create an elastic confined space for forcing nanoalloy formation (ESI Fig. S1†). Taking PtM/HCN (M = Cu, Co, Ni and Fe) as examples, the synthesis process of NAs/HCN was briefly introduced. First, Cu^2+^, Co^2+^, Ni^2+^ or Fe^3+^ was introduced separately into fresh SiO_2_*via* an ion-exchange process with NH_4_^+^ (ESI Fig. S2†). Subsequently, SiO_2_ spheres containing Cu^2+^, Co^2+^, Ni^2+^ or Fe^3+^ (ESI Fig. S3†) were calcined in air to transform these ions into the corresponding metal oxide (MO_*x*_/SiO_2_; ESI Fig. S4†) Then, polydopamine was coated onto MO_*x*_/SiO_2_ to form an elastic space. Meanwhile, Pt ions were brought into the elastic space (ESI Fig. S5 and S6†). Finally, PtM/HCN were obtained after pyrolyzing and removing SiO_2_. The as-fabricated 21 NAs/HCN nanoalloy materials ranging from LEAs to HEAs produced by the CESC strategy were used to form a nanoalloy library (ESI Fig. S7†)

**Fig. 1:**
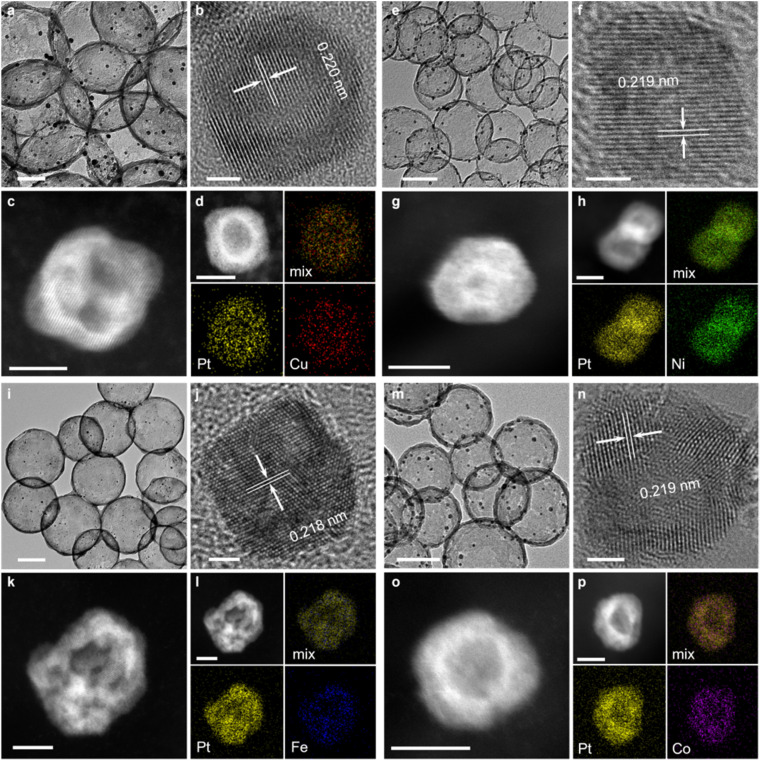
Characterization of the morphology and composition. TEM images and HRTEM images of PtCu/HCN as well as AC HAADF-STEM images and elemental mappings of framed PtM nanoparticles for (a–d) Cu, (e–h) Ni, (i–l) Fe, and (m–p) Co, respectively. Scale bars, 100 nm (a, e, l and m), 2 nm (b, f, j and n), and 5 nm (c, d, g, h, k, l, o and p).

Scanning electron microscopy (SEM), transmission electron microscopy (TEM) and aberration-corrected high-angle annular dark-field scanning transmission electron microscopy (AC HAADF-STEM) with energy-dispersive X-ray spectroscopy (EDX) were carried out to investigate the morphologies and microstructures of PtM/HCN (Fig. 1). SEM and TEM images revealed the uniform distribution of PtCu nanoparticles on HCN (Fig. 1a and ESI S8†). Interestingly, these PtCu nanoparticles possess many crystal plane dislocations and body defects and form nano-frame structures (Fig. 1b, c and ESI S9†). As shown in Fig. 1b, the lattice is assigned to the PtCu (111) diffraction peak observed in the powder X-ray diffraction (PXRD) result (Fig. 1d), revealing the PtCu alloyed composition.^26^ EDX mapping results further demonstrate the formation of the framed PtCu nanoalloy (Fig. 1d and ESI S10†). The morphologies and microstructures of PtNi/HCN, PtFe/HCN and PtCo/HCN were characterized by similar methods. From Fig. 1e–p, the framed PtNi, PtFe and PtCo nanoparticles were well distributed on HCN (ESI Fig. S11–S16†). Furthermore, PtCu/HCN has a higher degree of graphitization than others based on a lower *I*_D_/*I*_G_ value (ESI Fig. S17†)

**Fig. 2:**
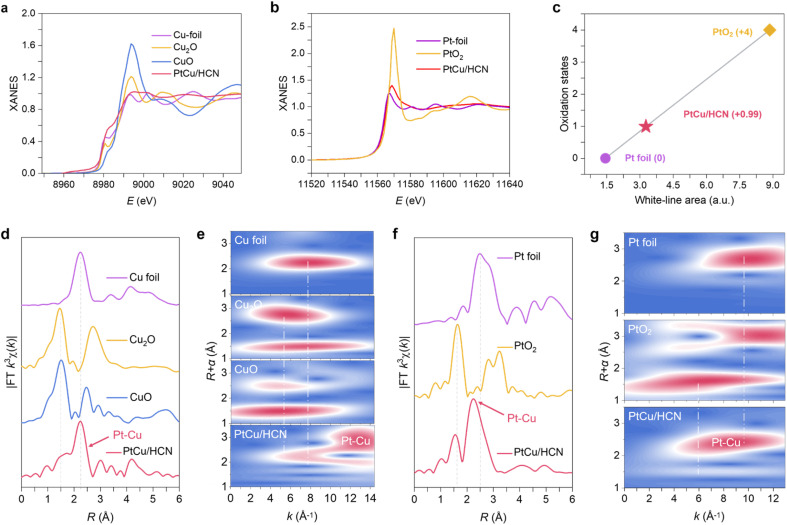
Structural characterization by XAFS spectroscopy. (a) Cu K-edge XANES spectra of PtCu/HCN and the references, (b) Pt L_3_-edge XANES curves of Pt foil, PtO_2_ and PtCu/HCN. (c) Fitted curves correlating the average oxidation state of Pt for PtCu/HCN, Pt foil and PtO_2_ at the Pt L_3_-edge. Fourier transforms of EXAFS spectra for the (d and e) Cu K-edge of PtCu/HCN and the references, and the corresponding wavelet transform for the *k*^3^-weighted EXAFS signals. (f and g) Pt L_3_-edge of PtCu/HCN and the references, and the corresponding wavelet transform for the *k*^3^-weighted EXAFS signals.

The X-ray absorption fine structure (XAFS) measurements were performed to further explore the chemical environment of Pt and Cu sites. The Cu K-edge X-ray absorption near-edge structure (XANES) spectra of PtCu/HCN and the references are shown in Fig. 2a PtCu/HCN has a median *E*_0_ at the Cu K-edge between those of Cu foil and Cu_2_O, suggesting the electron deficiency for Cu atoms. This result is consistent with the integrated conclusions of X-ray photoelectron spectroscopy (XPS) and Auger electron spectroscopy (AES; ESI Fig. S18†). From ESI S18a†, we can observe the existence of Cu(i).^27,28^ According to the Pt L_3_-edge XANES spectra of PtCu/HCN and the references, the valence of Pt atoms is situated between 0 and +4 (Fig. 2b). This result is also in agreement with the XPS spectra. From XPS results (ESI Fig. S19†) the Pt 4f spectra of PtCu/HCN can be curve-fitted into two pairs of peaks, which are assigned to Pt (0) and Pt (ii).^29^ Moreover, the oxidation state of Pt is calculated to be +0.99 from the integrated area under the white-line curve of the Pt L_3_-edge (Fig. 2c), suggesting a strong metal–support interaction.^30,31^ The quantitative result indicates that Pt atoms possess unoccupied 5d-electron states, which are beneficial to enhance HER activity.^32^

For the Fourier transformed (FT) *k*^3^-weighted EXAFS spectrum of PtCu/HCN at the Cu K-edge (Fig. 2d), the peak of 1.50 Å is assigned to the Cu–O bond. The peak of 2.24 Å shows a slight positive shift at the Cu K-edge compared to that of Cu foil, which belongs to the Pt–Cu bond. The FT-EXAFS spectrum of PtCu/HCN at the Pt L_3_-edge in Fig. 2f exhibits two main peaks at 1.56 Å and 2.24 Å, assigned to Pt–O and Pt–Cu bonds. On comparing the peak position of the Pt–Cu bond in PtCu/HCN with that of the Pt–Pt bond in Pt foil, a slightly negative shift in *R* space demonstrates the subtle change in atomic distance. EXAFS fitting was performed to extract the structural parameters and obtain the quantitative chemical configuration of Pt and Cu atoms (ESI Fig. S20, 21and Table S1†). The coordination number of Pt–Cu is approximately 8.97, and the average bond length of Pt–Cu is 2.24 Å. Wavelet transforms (WTs) were adopted to investigate Pt L_3_-edge and Cu K-edge EXAFS oscillations. The WT maximum at 13 Å^−1^ could be ascribed to the Cu–Pt bond and the WT maximum at 9 Å^−1^ corresponds to the Pt–Cu bond (Fig. 2e and g). Therefore, the FT-EXAFS and WT results further demonstrate the formation of the PtCu nanoalloy.

### 2.2 Electrocatalytic performances for the MOR

**Fig. 3:**
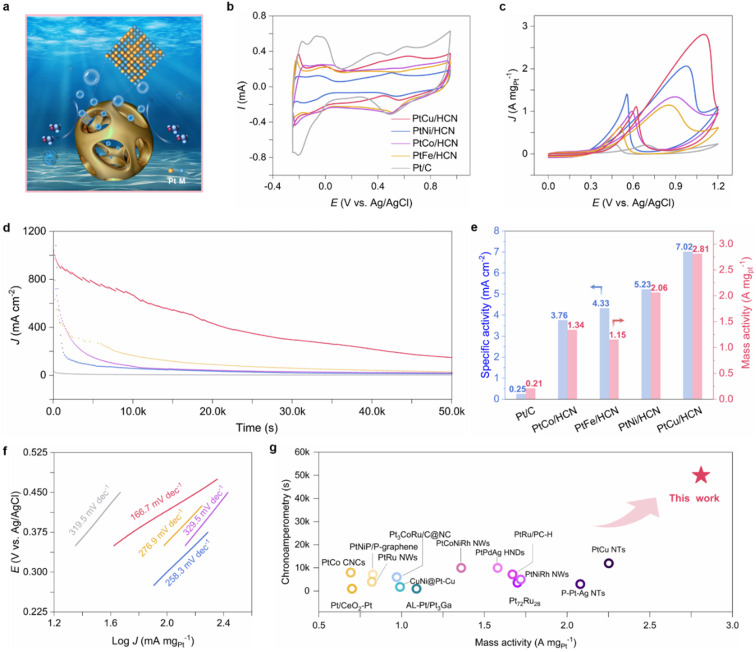
Electrooxidation of methanol performances. (a) Schematic illustration of the electrochemical tests of PtM/HCN. CV curves of PtM/HCN and commercial Pt/C in (b) 0.1 M HClO_4_ solution and (c) 0.1 M HClO_4_ + 1 M methanol solution at a sweep rate of 50 mV s^−1^ (d) CA curves of PtM/HCN and commercial Pt/C at 0.7 V *vs. E*_Ag/AgCl_. (e) Specific activity and mass activities of PtM/HCN and commercial Pt/C at peaks. (f) Tafel plots of PtM/HCN and commercial Pt/C and (g) mass activity and stability comparison of PtCu/HCN with other reported advanced Pt-based catalysts in 0.1 M HClO_4_ + 1 M methanol solution.

The MOR performances of the samples were investigated through a three-electrode system (Fig. 3). CVs were obtained in N_2_-saturated 0.1 M HClO_4_ solution to evaluate electrochemically active surface areas (ECSAs; Fig. 3b). According to ECSA results (ESI Table S2†), PtCu/HCN shows the highest ECSA compared to the others, implying the exposure of more active sites. The polarization curves and CVs of PtM/HCN and commercial Pt/C were obtained in N_2_-saturated 0.1 M HClO_4_ + 1 M methanol solution (Fig. 3c and ESI S22†). The specific and mass activities were calculated by normalizing the currents with ECSAs and Pt mass from inductively coupled plasma mass spectrometry (ICP-MS; ESI Table S3†). As shown in Fig. 3c and e, PtCu/HCN shows the highest specific and mass activities (7.02 mA cm^−2^ and 2.81 A mg_pt_^−1^), 28.1 and 13.4 times higher than those of commercial Pt/C (0.25 mA cm^−2^ and 0.21 A mg_pt_^−1^). And PtCu/HCN possesses the lowest Tafel slope compared to those of PtNi/HCN, PtFe/HCN, PtCo/HCN and commercial Pt/C, implying the fastest MOR kinetics (Fig. 3f). The MOR performance of PtCu/CN has exceeded that of most of the reported Pt-based catalysts (Fig. 3g and ESI Table S4†). Furthermore, the specific and mass activities of PtNi/HCN, PtFe/HCN and PtCo/HCN are 5.24 mA cm^−2^ and 2.06 A mg_pt_^−1^, 4.33 mA cm^−2^ and 1.15 A mg_pt_^−1^, and 3.76 mA cm^−2^ and 1.34 A mg_pt_^−1^, respectively, which are also superior to those of commercial Pt/C.

The anti-poisoning ability and stability are also key parameters for examining the electrocatalytic performances. The anti-poisoning ability could be evaluated by using the *J*_f_/*J*_b_ ratio of the forward scan to the backward scan. The ratio of *J*_f_/*J*_b_ for PtCu/HCN (2.53) is the highest compared to those of PtNi/HCN (1.47), PtFe/HCN (1.80), PtCo/HCN (1.34) and commercial Pt/C (0.98), implying an excellent poisoning toleration.^33–35^ This result may be attributed to the fast oxidation conversion of CO intermediates checked by *in situ* attenuated total-reflection surface-enhanced IR reflection absorption spectroscopy (ATR-SEIRAS; ESI Fig. S23†). Moreover, the long-term stability tests (LSTs) were conducted by keeping the potential at 0.7 V *vs.* Ag/AgCl (Fig. 3d). Apparently, after 50k seconds, PtCu/HCN still retains the largest current density and exhibits a loss of merely 5% of the peak current density (Fig. 3d, ESI S24 and S25†). Meanwhile, the structure and composition of PtCu/HCN exhibit little change, and each element still disperses uniformly (ESI Fig. S26–S28†), which demonstrates the potential of PtCu/HCN for the MOR.

### 2.3 Electrocatalytic performances for the HER

**Fig. 4:**
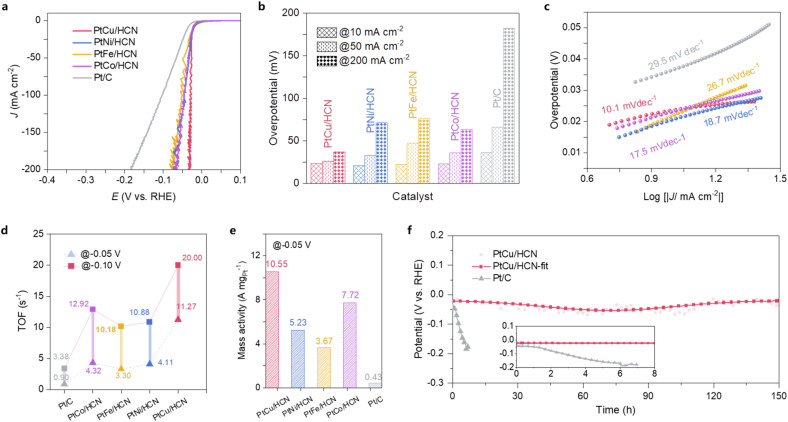
Electrocatalytic HER performances. (a) HER polarization curves, (b) the corresponding overpotential at different current densities, (c) Tafel plots, (d) a bar graph of comparative TOF values at two different overpotentials, and (e) a bar graph of mass activity by using normalized Pt loadings at a potential of −0.05 V (RHE) of PtM/HCN and commercial Pt/C obtained in 0.5 M H_2_SO_4_. (f) Chronopotentiometry curves recorded at 10 mA cm^−2^ of PtCu/HCN and commercial Pt/C in a 0.5 M H_2_SO_4_ solution; the range of 0–8 h was enlarged in the inset graph.

To further explore the performances of PtM/HCN in a reduction reaction, the corresponding HER activity and stability were tested in 0.5 M H_2_SO_4_ solution (Fig. 4). Fig. 4a and b show that PtCu/HCN has an ultralow overpotential (*η*_10_, *η*_50_ and *η*_200_ = 23.5, 26.3 and 37.3 mV) compared with commercial Pt/C (*η*_10_, *η*_50_ and *η*_200_ = 36.7, 66 and 182 mV), indicating a superior HER activity. Moreover, an ultrasmall Tafel slope (10 mV dec^−1^) also demonstrates the fastest HER kinetics of PtCu/HCN (Fig. 4c), which agrees with the results of electrochemical impedance spectra (EIS; ESI Fig. S29†). To gain an in-depth understanding of HER activity, turnover frequencies (TOFs) were calculated and are plotted in Fig. S30a.† PtCu/HCN displays the highest TOF values compared to those of the others and commercial Pt/C. Particularly, TOFs of 11.27 and 20.00 s^−1^ for PtCu/HCN obtained at −0.05 and −0.10 V *vs.* RHE are 12.5 and 5.9-fold higher than that of commercial Pt/C (Fig. 4d). In addition, mass activity is also an important factor for evaluating HER activity. The LSV curves of PtM/HCN and commercial Pt/C normalized by using the Pt mass are shown in ESI Fig. S30b†. As shown in Fig. 4e, the mass activity of 10.55 A mg_Pt_^−1^ for PtCu/HCN observed at −0.05 V *vs.* RHE is 24.5 times higher than that of commercial Pt/C (0.43 A mg_Pt_^−1^). Importantly, PtCu/HCN shows a better HER activity after the 10 000th CV cycle (ESI Fig. S31†), and operates stably at a current density of 10 mA cm^−2^ for at least 150 hours (Fig. 4f) without obvious structure changes (ESI Fig. S32†), surpassing previously reported acidic HER catalysts (ESI Table S5†). To investigate the operation potential, a homemade electrolyzer using PtCu/HCN as the cathode electrode and commercial RuO_2_ as the anode electrode (PtCu/HCN‖RuO_2_) was assembled. As shown in Fig. S33,† the assembled PtCu/HCN‖RuO_2_ electrolyzer realized a current density of 10 mA cm^−2^ at 1.49 V *vs.* RHE for 72 h with ignorable degradation, better than that of Pt/C‖RuO_2_ (1.75 V *vs.* RHE). Meanwhile, the assembled electrocatalytic system of a methanol–water electrolyzer using our PtCu/HCN as anode and cathode catalysts shows enhanced performance. The cell voltage was only 1.47 V *vs.* RHE at a driving current density of 10 mA cm^−2^, demonstrating excellent cycling stability (ESI Fig. S34†). To sum up, PtCu/HCN possesses great potential for electrolytic water splitting in an acidic medium.

### 2.4 DFT calculations

**Fig. 5:**
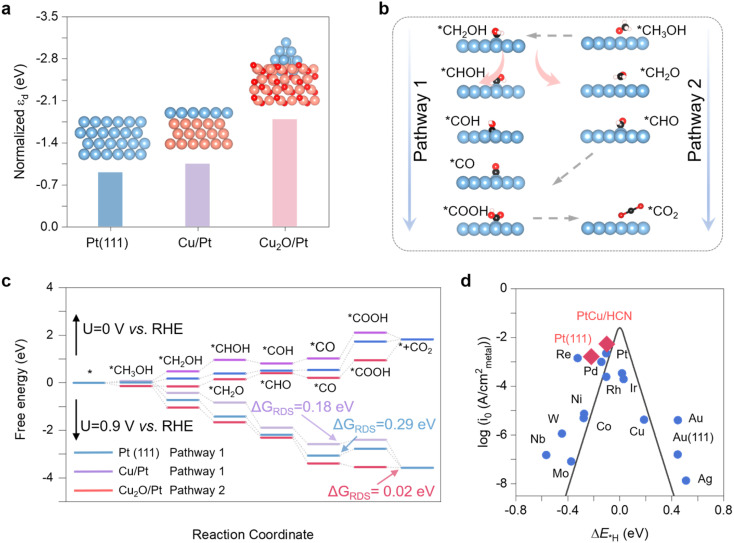
DFT calculations. (a) The average d-band center, *ε*_d(ava)_, of Pt atoms in Pt (111), CuPt, and Cu_2_O/Pt. The insets show the Pt (111), CuPt, and Cu_2_O/Pt models. The red, gold, and indigo balls denote O, Cu, and Pt atoms, respectively. (b) The free energy profiles of methanol oxidation on Pt (111), Cu/Pt, and Cu_2_O/Pt at 0 and 0.9 V *vs.* RHE. The free energy changes of the rate-determining steps are listed for the three different surfaces. (c) The possible MOR pathways on Pt (111), CuPt, and Cu_2_O/Pt. (d) A volcano plot of experimentally measured exchange current density as a function of the DFT-calculated Gibbs free energy of adsorbed atomic hydrogen. The simple kinetic model proposed by Nørskov and co-workers to explain the origin of the volcano plot is shown as solid lines.

To further elucidate the enhanced activity of PtCu/HCN, we applied DFT calculations to understand the highly catalytic sites and the catalytic mechanism. We first constructed three catalyst models, Pt (111), CuPt, and Cu_2_O/Pt, shown in the inset of Fig. 5a. From the Bader charge calculations, we found that an electron transfer of 0.26 e^−^ occurred *via* the Pt–O bond (ESI Fig. S35†), resulting in the increased Pt oxide state and decreased Cu oxide state compared to those of Cu_2_O (ESI Table S6†). The average d-band center *ε*_d(ava)_ of Pt atoms (−1.79 eV) in Cu_2_O/Pt is lower than that in Pt (111) (−0.90 eV) and CuPt (−1.04 eV), which leads to the attenuation of the adsorption on PtCu/HCN (Fig. 5a). Considering two possible MOR pathways shown in Fig. 5b, we investigated all elementary steps on Pt (111), CuPt, and Cu_2_O/Pt (ESI Fig. S36–S38†) and the corresponding free energy changes along the reaction coordinate (Fig. 5c and ESI Fig. S39†). At *U* = 0 *vs.* RHE, the total MOR process is uphill on Pt (111), CuPt, and Cu_2_O/Pt, indicating that the MOR process cannot proceed spontaneously. The MOR process prefers pathway 1 on CuPt and pathway 2 on Cu_2_O/Pt because of the prohibitively energetic change of generating *CH_2_O on CuPt and converting *COH to *CO on Cu_2_O/Pt. When the applied potential increases to 0.9 V *vs.* RHE, all electron transfer steps on Pt (111) and CuPt are endergonic except for the *CO oxidation step. Meanwhile, the CO_2_ generation step on Cu_2_O/Pt has the lowest free energy change. These results indicate that *COOH generation is the rate-determining step (RDS) for Pt (111) and CuPt, while the CO_2_ release step is the RDS for Cu_2_O/Pt. The Δ*G*_RDS_ on Pt (111), CuPt, and Cu_2_O/Pt is 0.29, 0.18 and 0.02 eV, respectively, suggesting that Cu_2_O/Pt has the highest MOR activity at 0.9 V *vs.* RHE. This verifies that the Cu_2_O/Pt sites are the catalytic sites for the MOR with high activity. As shown in Fig. 5d, the HER volcano plot shows that the catalysts lying at the left slope bind H atoms too strongly while ones lying at the right slope adsorb H weakly. Superior HER catalysts exhibit a higher exchange current density with *H adsorption energies (ΔG*_H_) close to 0 eV. Compared to Pt (111), PtCu/HCN possesses an enhanced HER exchange current density because of a relatively low ΔG*_H_ of PtCu/HCN. Besides, own to both excellent MOR and HOR, Cu_2_O/Pt exceeds Pt cluster loaded on other oxides, including Fe_2_O_3_/Pt, CoO/Pt, and NiO/Pt (ESI Fig. S40 and S41†). In sum, the theoretical results show that Cu_2_O/Pt with a low d-band center and adsorption ability has the highest electrocatalytic activities for both the MOR and HER.

### 2.5 The generality and merits of the CESC strategy

**Fig. 6:**
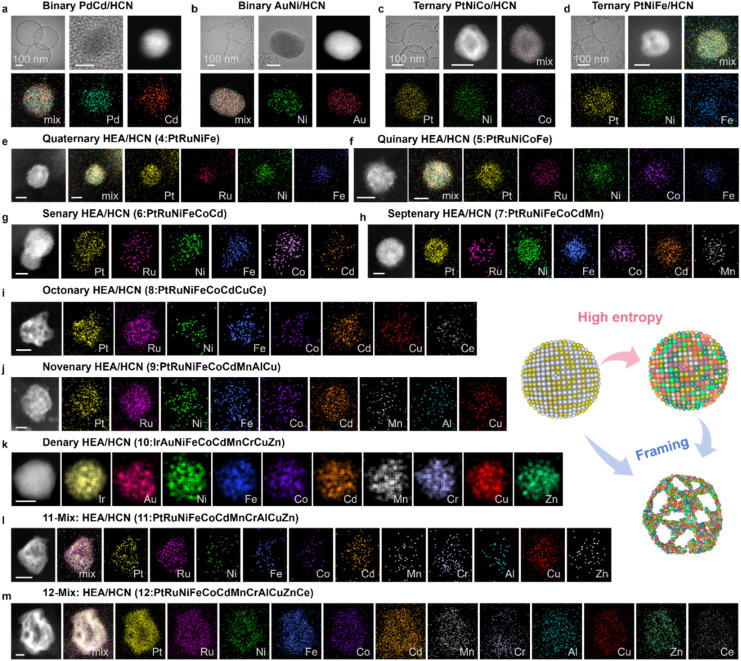
The generality of the CESC strategy. TEM images and HRTEM images of nanoalloys and AC HAADF-STEM images and corresponding elemental maps of other supported alloys: (a and b) binary PdCd and AuNi. Scale bar, 5 nm. (c and d) Ternary PtNiCo and PtNiFe. Scale bar, 5 nm. (e) Quaternary PtRuNiFe, (f) quinary PtRuNiFeCo, (g) senary PtRuNiFeCoCd, and (h) septenary PtRuNiFeCoCdMn. Scale bar, 2 nm. (i) Octonary PtRuNiFeCoCdCuCe, (j) novenary PtRuNiFeCoCdMnAlCu, (k) denary IrAuNiFeCoCdMnCrCuZn, (l) 11-element PtRuNiFeCoCdMnCrAlCuZn and (m) 12-element PtRuNiFeCoCdMnCrAlCuZnCe nanoalloys. Scale bar, 5 nm.

Fig. 6 and ESI S42–S58† demonstrate the utility and advantages of our CESC strategy for the synthesis of nanoalloys ranging from LEAs to HEAs. Compared with traditional synthetic methods and new preparation techniques,^22,36^ the CESC strategy is easier to generalize and popularize due to the introduction of the cation-exchange process, which had been certified to successfully construct a library containing at least 21 nanoalloys with different entropies. Specifically, the library consists of 9 LEAs (*e.g.*, RuSn, PdNi, PdCd, AuNi, IrCo, *etc*.; ESI Fig. S42–S46†), 4 MEAs (*e.g.*, PtFeCu, PtRuNiFe, *etc*.; ESI Fig. S47–S50†) and 8 HEAs (*e.g.*, PtRuNiCoFeCdMnCrAlCuZnCe, *etc*.; ESI Fig. S51–S58†). From Fig. 6, the as-prepared representative nanoalloys show a superfine size and uniform element distribution. With the increase in metal element species, we can find it easier to form multi-element HEAs (Fig. 6). Regardless of the LEAs or HEAs, the distribution of each metal is uniform without segregation, matching the XRD results. Moreover, the framing of nanoalloys can be adjusted by controlling the feeding dosages of earth-abundant metals (ESI Fig. S59†). The corresponding characterization studies are shown in ESI Fig. S40–S56† and indicated the smooth formation of nanoalloys with different entropies. And other electrocatalytic applications, like the ORR, can also be realized (ESI Fig. S60†). In addition, the activity and stability of the prepared high-entropy alloys are summarized in ESI Fig. S61†, and the corresponding high-temperature resistance to sintering is also demonstrated (ESI Fig. S62–S69†). The above results demonstrate that the merits of the CESC strategy we developed are not only conducive to synthesizing nanoalloys generally from LEAs to HEAs, but also applicable to the preparation of nanoalloys with nano-frames structures. Despite the differences in the number of elements, the construction of the framework structure of NAs can be achieved by adjusting the amounts of non-precious metals, which is beyond the capability of conventional synthetic strategies (ESI Table S2†). In brief, our CESC approach is elemental customizable and has great generality, providing an opportunity to merge dozen-element metal into nanoalloys.

## 3 Conclusions

In summary, we have developed a simple and general CESC strategy and successfully prepared at least 21 noble-based nanoalloy materials ranging from LEAs to HEAs. Amazingly, the CESC strategy works well for nanoalloys with nano-frame structures. In our library, LEA PtCu/HCN exhibits excellent activity and stability for the MOR and HER. The outstanding electrocatalytic performances of PtCu/HCN have been explained by DFT calculations. Our work provides an opportunity to synthesize noble-based nanoalloys ranging from LEAs to HEAs, and even nanoalloys with nano-frame structures, suggesting possibilities to explore the extraordinary electrocatalytic performances of these nanoalloy materials and their other applications.

## Date availability

All data are available from the authors upon reasonable request.

## Author contributions

Y. H. conceived the project. G. C., J. Z. and W. C. carried out most of the experiments and co-wrote the manuscript. Z. W. and R. L. performed the theoretical calculations. C. M. performed partial characterization of the materials. G. C., W. C. and R. L. participated in data analysis. All authors discussed the results and commented on the manuscript.

## Conflicts of interest

The authors declare no conflict of interest.

## Supplementary Material

SC-015-D4SC03142C-s001
