# Supplementary material for: Designing the framework structure of noble-metal based nanoalloy catalysts driving redox electrocatalysis
Source: Chem Sci. 2024 Jun 26;15(31):12550–8. doi: 10.1039/d4sc03142c (PMC11304777; doi:10.1039/d4sc03142c)
Supplement: SC-015-D4SC03142C-s001 [file SC-015-D4SC03142C-s001.pdf]

Electronic Supplementary Material (ESI) for Chemical Science.
This journal is © The Royal Society of Chemistry 2024

Electronic Supplementary Information

# Designing the framework structure of noble-metal based nanoalloy catalysts driving redox electrocatalysis

Guanzhen Chen [a, †], Jie Zhang [a, †], Wen Chen [a, †], Ruihu Lu [b, †], Chao Ma[c], Ziyun Wang [b, *], and Yunhu Han [a, *]

[a] Institute of Flexible Electronics (IFE) and Frontiers Science Center for Flexible Electronics, Northwestern Polytechnical University, Xi'an 710072, China

E-mail: iamyhhan@nwpu.edu.cn

[b] School of Chemical Sciences, The University of Auckland, Auckland 1010, New Zealand

E-mail: ziyun.wang@auckland.ac.nz

[c] Department of Chemistry, Tsinghua University, Beijing 100084, China

*Corresponding author: ziyun.wang@auckland.ac.nz (Z.W);
iamyhhan@nwpu.edu.cn (Y.H)

Materials and Methods

**Chemicals and Materials.** The chemicals and reagents were used as received. Analytical grade copper(II) nitrate trihydrate ($Cu(NO_3)_2 \cdot 3H_2O$, 99%), nickel nitrate hexahydrate ($Ni(NO_3)_2 \cdot 6H_2O$, 99%), iron(III) nitrate nonahydrate ($Fe(NO_3)_3 \cdot 9H_2O$, 99%), cobalt(II) nitrate hexahydrate ($Co(NO_3)_2 \cdot 6H_2O$, 99%), cadmium(II) nitrate tetrahydrate ($Cd(NO_3)_2 \cdot 4H_2O$, 99%), aluminum nitrate nonahydrate ($Al(NO_3)_3 \cdot 9H_2O$, 99%), chromium(III) nitrate nonahydrate ($Cr(NO_3)_3 \cdot 9H_2O$), zinc(II) nitrate hexahydrate ($Zn(NO_3)_2 \cdot 6H_2O$), stannous(II) chloride dihydrate ($SnCl_2 \cdot 2H_2O$), and manganese (II) chloride dihydrate ($MnCl_2 \cdot 2H_2O$, 99%) were purchased from Aladdin Chemical Reagent Co., Ltd. Cerium(III) Nitrate Hexahydrate ($Ce(NO_3)_3 \cdot 6H_2O$, 99%) was purchased from Shanghai Titan Technology Co., Ltd. The commercial Pt/C catalyst is 20% by wt. of ~3 nm platinum nanoparticles on XC-72 carbon and nafion D-521 dispersion (5% w/w in water and 1-propanol) were obtained from Alfa Aesar. All other chemicals were purchased from Shanghai Macklin Biochemical Co., Ltd., China, including hex chloroplatinic (IV) hexahydrate ($H_2PtCl_6 \cdot 6H_2O$, 99%), iridium (IV) chloride hydrate ($IrCl_4 \cdot nH_2O$, 99.9%), Palladium tetraammine dichloride (II) ($Pd(NH_3)_4Cl_2$, 98%), ruthenium(III) triacetate ($C_6H_9O_6Ru$, 98%), tetra chloroauric (III) trihydrate ($HAuCl_4 \cdot 3H_2O$, 98%), ammonium hydrogen difluoride ($NH_4HF_2$, 98.5%), and dopamine ($C_8H_{11}NO_2 \cdot HCl$, 98%). Doubly distilled deionized water (18.2 MΩ) was used for all experiments.

**Preparation of $SiO_2$ template.** The preparation of silica spheres involves the ammonia-catalyzed hydrolysis and condensation of TEOS in an aqueous ethanol solution by the classical stöber method according to a modified previous report [1]. Typically, 15 mL of absolute ethanol, 5 mL of DI water, and 0.7 mL of 28% $NH_3 \cdot H_2O$ were mixed and stirred. A total of 0.6 mL of TEOS was added into the mixture quickly. After a reaction time of about 10 h, the silica spheres were isolated by centrifugation. Then the white precipitate was washed with ethanol three times and dried standby application.

**Preparation of $MO_x/SiO_2$ (M=Cu, Ni, Fe, Co).** Taking the synthesis of the $CuO_x/SiO_2$ as an example, 500 mg $SiO_2$ nanoparticles and 1.0 g $Cu(NO_3)_2 \cdot 6H_2O$ was dispersed in 40 mL DI water to form a clear blue solution, stir the reaction at 80 °C for 16 h. The obtained product was separated by centrifugation and dried at 60 °C for 2 h. The resultant $Cu^{2+}/SiO_2$ products was calcined at 400 °C for 30 min in an air atmosphere to obtain $CuO_x/SiO_2$. The same procedure was utilized for synthesis of $NiO_x/SiO_2$, $FeO_x/SiO_2$ and $CoO_x/SiO_2$ by using $Ni(NO_3)_2 \cdot 6H_2O$, $Fe(NO_3)_3 \cdot 9H_2O$ and $Co(NO_3)_2 \cdot 6H_2O$ instead of $Cu(NO_3)_2 \cdot 3H_2O$.

**Preparation of PtM/HCN (M = Cu, Ni, Fe, Co) catalysts.** The as-prepared 500 mg $CuO_x/SiO_2$ was dispersed in 40 mL of water-ethanol solution. Then the 10 mL water-ethanol solution including 400 μL (50 mg $mL^{-1}$) $H_2PtCl_6 \cdot 6H_2O$ was injected into it under magnetic stirring for 6 h, and then the 30 mL water-ethanol (V: V=1:1) solution involving 227.57 mg dopamine was poured into the above solution and continuously stirred another 3 h. After that, 10 mL of water-ethanol (V: V=1:1) solution containing 2 mL (28 wt%) $NH_3 \cdot H_2O$ was added to the above mixture solution and reacted for 12 h. Finally, the precursor was collected by centrifugation, washed twice with ethanol, and dried at 60°C overnight. The obtained precursor was subjected to pyrolysis treatment at 900 °C under Ar atmosphere for 2 h with a

heating rate of 5 °C·min$^{-1}$. Then obtained samples were etched by excessive 2.0 M $NH_4HF_2$ aqueous solution at 80 °C for 2 h to remove the $SiO_2$ template, washed with water and ethanol, and dried under vacuum at 60 °C overnight to obtained the PtCu/HCN. (Water-ethanol solution: containing ethanol and DI water). The same procedure was utilized for synthesis of PtNi/HCN, PtFe/HCN and PtCo/HCN by using $NiO_x/SiO_2$, $FeO_x/SiO_2$ or $CoO_x/SiO_2$ instead of $CuO_x/SiO_2$.

The syntheses of equivalent binary (RuSn, PdNi, PdCd, AuNi, IrCo), ternary (PtNiCo, PtNiFe, PtFeCu), quaternary (PtRuNiFe), quinary (PtRuNiFeCo), senary (PtRuNiCoFeCd), septenary (PtRuNiCoFeCdMn) Octonary (PtRuNiFeCoCdCuCe), Novenary (PtRuNiFeCoCdMnAlCu), Denary (PtRuNiFeCoMnCrCuZnCe), 11-element PtRuNiFeCoCdMnCrAlCuZn and 12-element (PtRuNiFeCoCdMnCrAlCuZnCe) HAEs distributed on hollow nitrogen-doped carbon were performed using the similar method except for changing the amounts of the salts and annealing temperature from 600 to 900 °C.

**Characterization.** The microstructure and morphology of the prepared materials was observed by a scanning electron microscope (SEM; Gemini SEM 300 at 0.02~30 kV), transmission electron microscope (TEM; HT7800) and high-resolution transmission electron microscopy (HRTEM; FEI-Themis Z TEM/STEM operated at 300 kV and equipped with double spherical aberration (Cs) correctors, Themis Z TEM/STEM equipped with high angle annular dark field (HAADF) and annular bright field (ABF) detectors). High angle annular dark field (HAADF) images were acquired using the Themis Z with a 59~100 mrad inner-detector angle. The attainable resolution of the probe defined by the objective pre-field is 60 picometers. A Super X Windowless X-ray detector was used to collect the energy dispersive spectra (EDX). XPS was performed on a KULVAC PHI Quantera X-ray photoelectron spectrometer and analyzed by the Avantage software. X-ray diffraction (XRD) was performed using a D8 Advanced (Bruker AXS, WI, USA) with a scan rate of 2° min$^{-1}$. The metal contents of the CN supported the nanoalloys was detected by an Optima 7300 DV inductively coupled plasma mass spectrometer (ICP-MS). The X-ray absorption find structure spectra (Pt $L_3$-edge, Cu K-edge) were collected at 1W1B station in Beijing Synchrotron Radiation Facility (BSRF). The storage rings of BSRF were operated at 2.5 GeV with an average current of 250 mA. Using Si (111) double-crystal monochromator, the data collections were carried out in S4 transmission/fluorescence mode using ionization chamber. All spectra were collected in ambient conditions. The temperature-programed desorption of CO (CO-TPD) was performed by the VDSorb-91x chemisorption analyzer. Raman spectra were conducted by using an Invia reflex Raman microscopy system (RENISHAW). Fourier transform Infrared (FT-IR) spectrum was performed by using a Bruker spectrometer (Horiba, Germany) according to the attenuated total reflectance method (ATR).

**In situ attenuated total reflection surface-enhanced IR absorption spectroscopy (ATR-SEIRAS) measurements.** *In situ* ATR-SEIRAS spectrum was gathered by a FT-IR spectrometer (Thermo Scientific Nicolet iS50) equipped with MCT-A detector. The catalysts inks are prepared by mixing 2.5 mg electrocatalysts, 0.4 mL ethanol, and 25 μL of nafion. 4 μL of ink solution is dropped onto the basal plane of a hemicylindrical Si prism. The Si prism was assembled in a spectro-electrochemical cell with Pt wire as a counter electrode, Ag/AgCl

electrode as reference electrode, and 0.1 M $HClO_4$+1 M $CH_3OH$ solution as electrolytes. All spectra are collected at a resolution of 4 $cm^{-1}$ and each single-beam spectrum is an average of 200 scans.

**Electrochemical measurements.** In the three-electrode system, we used glassy carbon electrode (5 mm in diameter) as the working electrode, Ag/AgCl electrode and graphite rod as the reference electrode and counter electrode, respectively. The working electrode was prepared as follows: 2.5 mg of catalyst was dispersed in the mixture of ethanol (0.4 mL) and nafion (5 wt%, 25 μL) under ultrasonication for 2 h. Then, the above suspension (8 μL) was dropped on glassy carbon electrode and dried at room temperature. The mass loading of catalysts was 0.24 mg $cm^{-2}$. Electrochemical activation of the catalysts on RDE was performed at an electrochemical workstation (CHI760E) by potential-cycling between -0.15 V and 1.05 V at a sweeping rate of 50 $mV \cdot s^{-1}$ in $N_2$-saturated 0.1 M $HClO_4$ for 50 cycles. For the MOR stability tests, chronoamperometric tests were performed at a potential of 0.7 V vs. Ag/AgCl, and 2000th CVs were also performed to evaluate the stability of catalysts. The electrochemical active surface areas (ECSAs) were obtained by integrating the hydrogen adsorption charge between −0.25 V and 0.10 V vs. Ag/AgCl after the double-layer correction. The ECSA of Pt can be calculated based on the following equation:

$$ECSA = \frac{Q_H}{m \times q_H} \#(1)$$

where $Q_H$ is the charge for $H_{upd}$ adsorption, is $m$ the loading amount of metal, and $q_H$ is the charge required for monolayer adsorption of hydrogen on a Pt surface and is assumed to be 210 μC $cm_{Pt}^{-2}$. For each catalyst, MOR measurements were carried out in $N_2$-saturated 0.1 $HClO_4$ and 1 M methanol solution at room temperature at a sweep rate of 50 $mV \cdot s^{-1}$.

**Hydrogen evolution tests.** The 2.5 mg catalysts were dispersed in a mixture of 400 μL ethanol and 20 μL nafion solution, after sonication for 1 h, catalyst with the concentration of 5.95 mg $mL^{-1}$ was obtained. Electrochemical measurements were conducted on a CHI 760E Electrochemical Workstation (Shanghai Chenhua Instrument Corporation, China) in a conventional three-electrode cell. The graphite rod electrode as the counter electrode and an Ag/AgCl (saturated KCl solution) as the reference electrode. The working electrode was a glassy carbon electrode (GCE, diameter: 5 mm, area: 0.19625 $cm^2$). 10 μL of the catalyst were dropped onto the GCE surface for further electrochemical tests. All the potentials reported in this work were converted to the reversible hydrogen electrode (RHE). Cyclic voltammograms (CVs) were performed in $N_2$-saturated 0.5 M $H_2SO_4$ solution from 0 to 1.2 V vs. RHE at a scan rate of 50 $mV \cdot s^{-1}$. Electrochemical impedance spectroscopy (EIS) measurements were measured at −50 mV vs. RHE in the frequency range from 10 kHz to 0.01 Hz in $N_2$-saturated 0.5 M $H_2SO_4$ solution. The chronopotentiometry tests were conducted in 0.5 M $H_2SO_4$ and the catalysts were loaded on the carbon cloth (0.595 mg $cm^{-2}$). The electrochemical water splitting tests in 0.5 M $H_2SO_4$ were carried out in a two-electrode system with the PtCu/HCN ‖ $RuO_2$ and Pt/C ‖ $RuO_2$ coupled catalysts. The catalysts were loaded on carbon cloth (0.5 M $H_2SO_4$) at a loading of 0.595 mg $cm^{-2}$. To assess the long-term water splitting stability of PtCu/HCN ‖ $RuO_2$, the Continuous potentiometric V–t

measurement of PtCu/HCN ∥ $RuO_2$ was employed under a constant current density of 10 mA $cm^{-2}$ for 72 h.

**TOF calculation.** the TOF is calculated by followed equation (2)-(5):

$$TOF = \frac{N_{H_2\ per\ unit\ area}}{N_{active\ \ site\ per\ unit\ area}} \#(2)$$

$$N_{H_2\ per\ unit\ area} = \left(|J|\frac{mA}{cm^{-2}}\right)\left(\frac{1\ C\ s^{-1}}{1000\ mA}\right)\left(\frac{1\ mol\ e^{-1}}{96485,3\ C}\right)\left(\frac{1\ mol}{2\ mol\ e^{-1}}\right)\left(\frac{6.022\times10^{23}\ molecules\ H_2}{1\ mol\ H_2}\right) = 3.12\times10^{15}\frac{s^{-1}}{cm^2}|J| \#(3)$$

$$N_{active\ site\ per\ unit\ area} = N_{Pt\ atom} = \left(\frac{catalyst\ loading\ per\ unit\ area\ (x\ g\ cm^{-1})\times Pt\ wt.\%}{Pt\ M_w(g\ mol^{-1})}\right)\left(\frac{6.022\times10^{23}\ Pt\ atoms}{1\ mol\ Pt}\right) \#(4)$$

Where $J$ is the measured current density. $M_w$ is the atomic mass of platinum.

For example, the $N_{active\ site\ per\ unit\ area}$ for PtCu/HCN was calculated from:

$$N_{active\ site\ per\ unit\ area} = \left(\frac{0.303\times10^{-3}\ g\ cm^{-1}\times 9.59\ wt.\%}{195.084\ g\ mol^{-1}}\right)\left(\frac{6.022\times10^{23}\ Pt\ atoms}{1\ mol\ Pt}\right) = 8.98\times10^{16}\ cm^{-2}$$

Hence, the TOF for PtCu/HCN can be calculated from:

$$TOF = \frac{3.12\times10^{15}\frac{s^{-1}}{cm^2}}{8.98\times10^{16}\ cm^{-2}}\times|J| = 0.0347\times|J|\ s^{-1} \#(5)$$

**Computation methods.** Spin-polarized DFT calculations were conducted using the Vienna *ab* initio simulation package (VASP) [34-36]. We adapted the generalized gradient approximation in the Perdew−Burke−Ernzerhof functional (GGA-PBE) to describe the electron exchange and correlation energy [5], and the interaction between core electrons and valence electrons is described by the frozen-core projector-augmented wave (PAW) method with a cut-off energy of 500 eV [38, 39]. The long-range vdW interactions between atoms is finely described by the DFT-D3 correction method in Grimme's scheme [8]. the criteria of energy and force convergence are set to $1.0\times10^{-5}$ eV per atom and 0.02 eV·$Å^{-1}$, respectively, for geometry optimization. And a Γ-centred Monkhorst–Pack *k*-point mesh grid of 3 × 3 × 1 is employed for all structural optimizations [9]. For the calculations of $Pt/Cu_2O$, due to the strong correlation effect among the partially filled Cu 3d states, we used the Hubbard parameter, U, for the Cu 3d electrons to illustrate the on-site Coulomb interaction, which is the well-known

DFT+U method [10]. According to previous work [11], the value of U-J of $Cu_2O$ was set as 6.0 eV.

For each element step of MOR, the Gibbs free energy $\Delta G_i$ (i = 1~6) can be calculated using the following equation.

$$\Delta G = \Delta E + \Delta E_{ZPE} - T\Delta S + neU \#(6)$$

where $\Delta E$ is the total energy of reactions obtained from DFT calculations. $\Delta E_{ZPE}$ and $\Delta S$ represent the zero-point energy and entropic changes, respectively, which are obtained *via* vibrational frequencies computations with harmonic approximation and neglecting contributions from the slab. According to the computational hydrogen electrode (CHE) model proposed by Nørskov *et al* [12]. The free-energy change of $1/2H_2 \rightarrow H^+ + e^-$ reaction is treated to be zero at the potential of 0 and the free energy of proton and electron is set as the $1/2G_{(H2)}$ and $\Delta G_{*H} = \Delta E_H + 0.24$. U is the electrode applied potential relative to RHE, $e^-$ is the elementary charge transferred and n is the number of proton-electron pairs transferred. The vacuum space was 15 Å to avoid artificial interactions between periodic images in *z* direction.

**HER kinetic models.** HER kinetic models proposed by Nørskov and co-workers to describe the correlation of experimental exchange current density and free energy change of adsorbed atomic hydrogen ($\Delta G_{*H}$) [12]. A volcano-shaped relation exhibited Catalysts with a negative $\Delta G_{*H}$ can be limited in the desorption of $H_2$ molecules, therefore poisoning the active sites and proceed next HER cycle. Catalysts with a positive $\Delta G_{*H}$ have a difficulty in desorbing *H to convert $H_2$. Therefore, strong and weak adsorption of *H will block the kinetics of HER process, lowering the exchange current density. A feasible $\Delta G_{*H}$ close to 0 eV can balance the *H adsorption and $H_2$ desorption, showing a highest exchange current density to reach the volcano peak. The blue point in Figure originated from Nørskov's works [12]. The $\Delta G_{*H}$ of Pt(111) is obtained from previous works [13]. The exchange current density (*j*) is obtained from the experimental TOF according the equation listed below [14].

$$j = -n\rho e TOF_{H_2} \#(7)$$

Where $n$ denotes the number of transferring electrons in certain reaction. Here is 2 for HER. $\rho$ is the density of active sites, which is assumed as $1.5 \times 10^{15}$ site $cm^{-2}$ [15]. The TOF is obtained from our experiments.

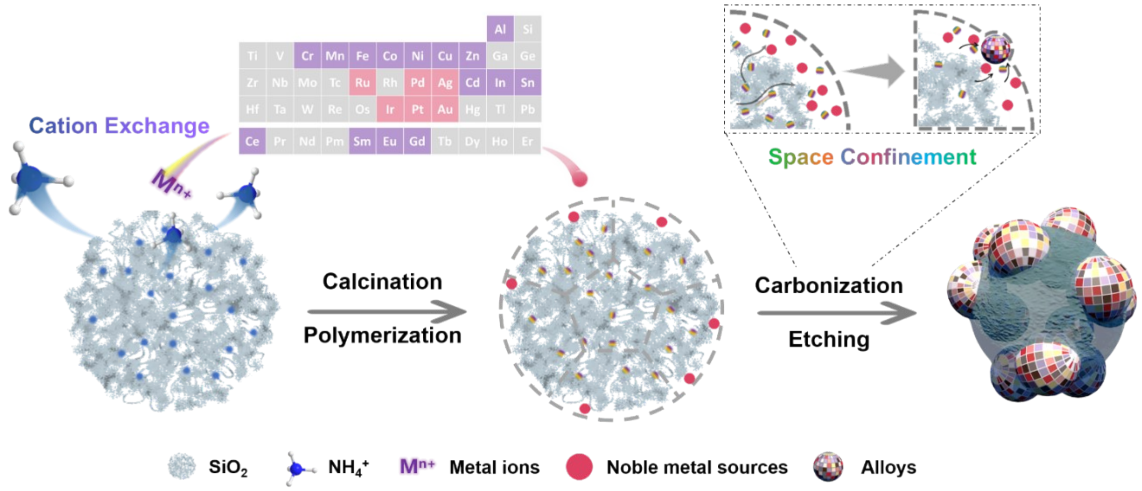


**Fig. S1** Schematic illustration of preparation for NAs/HCN material library.

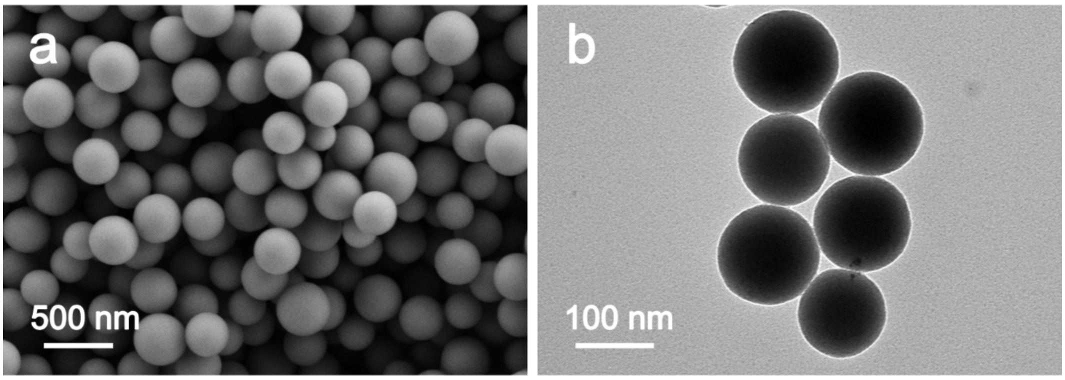


**Fig. S2** (a) SEM image and (b) TEM image of prepared $SiO_2$ microspheres containing $NH_4^+$.

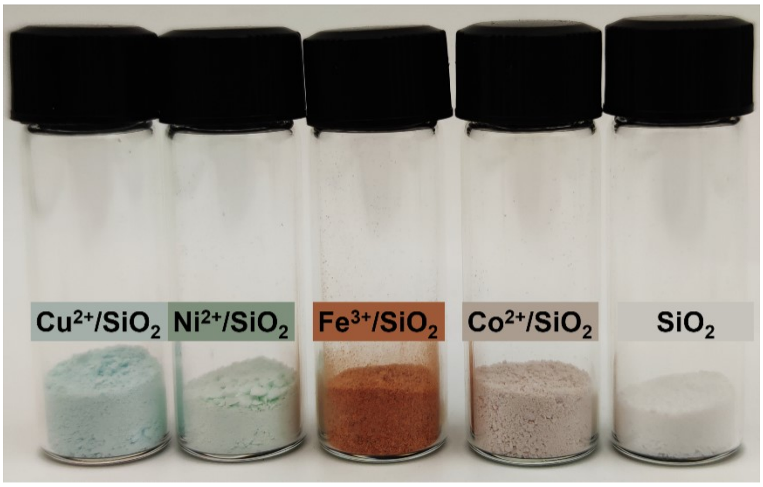


**Fig. S3** Optical image comparison of initial $SiO_2$ particles and Mn+/$SiO_2$ (M=Cu, Ni, Fe, Co) obtained by cation exchange progress.

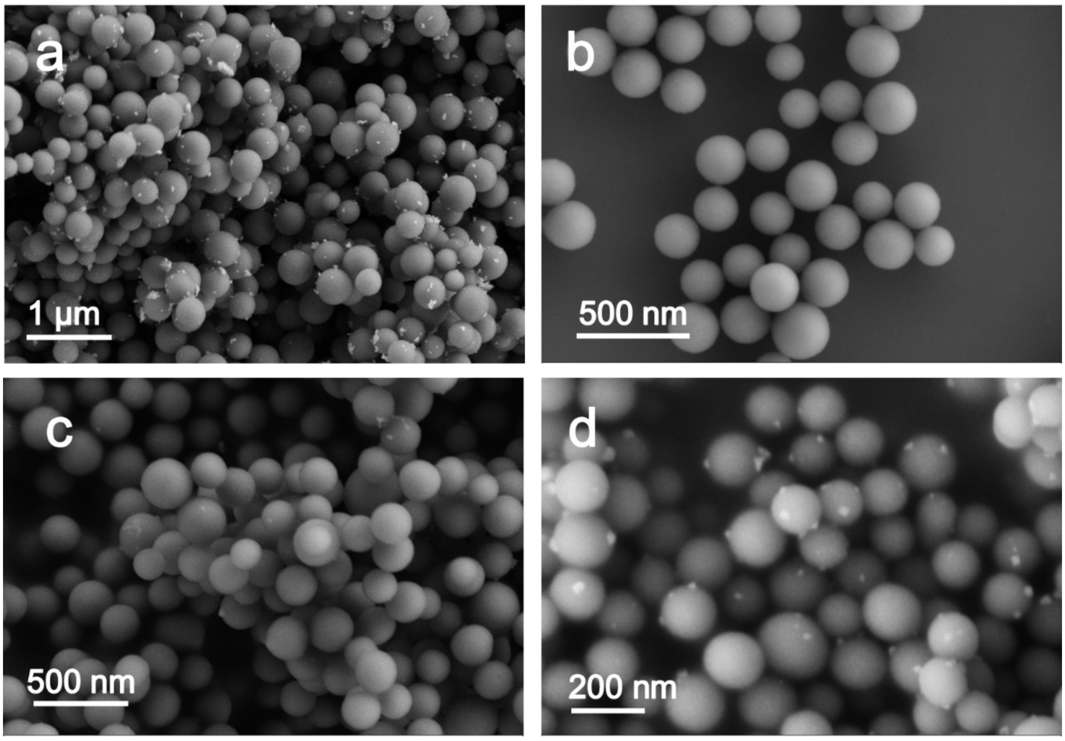


**Fig. S4** SEM images of the $MO_x/SiO_2$ (M= (a) Cu, (b) Ni, (c) Fe, (d) Co) prepared by cation-exchange reaction and further calcination in air.

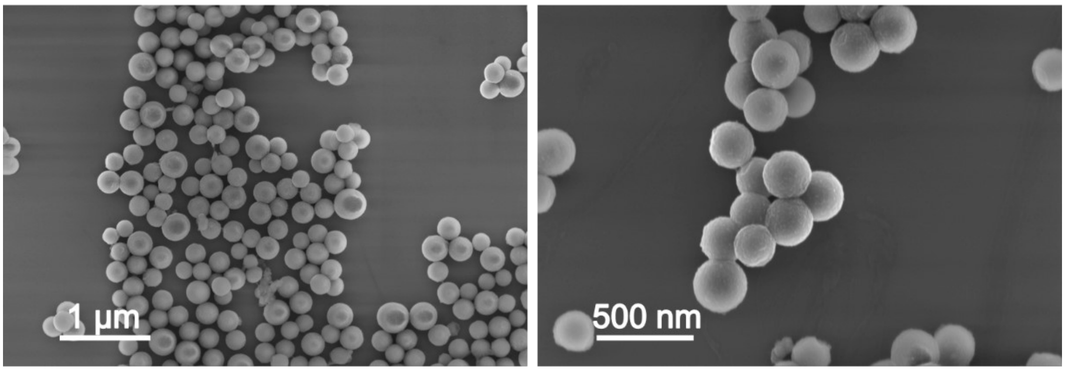


**Fig. S5** SEM images of $PtCl_6^{2-}/MO_x/SiO_2$ coated with PDA layer.

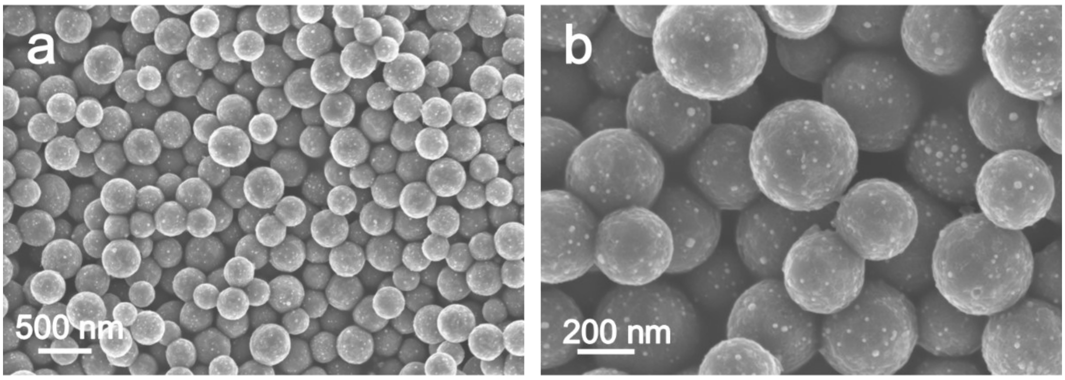


**Fig. S6** SEM images of the obtained $SiO_2$@PtM/CN before the template removal, which confirms the uniform distribution of surface nanoparticles as well as the maintenance of spherical structure.

21 HCN Supported Noble-metal-based NAs

a

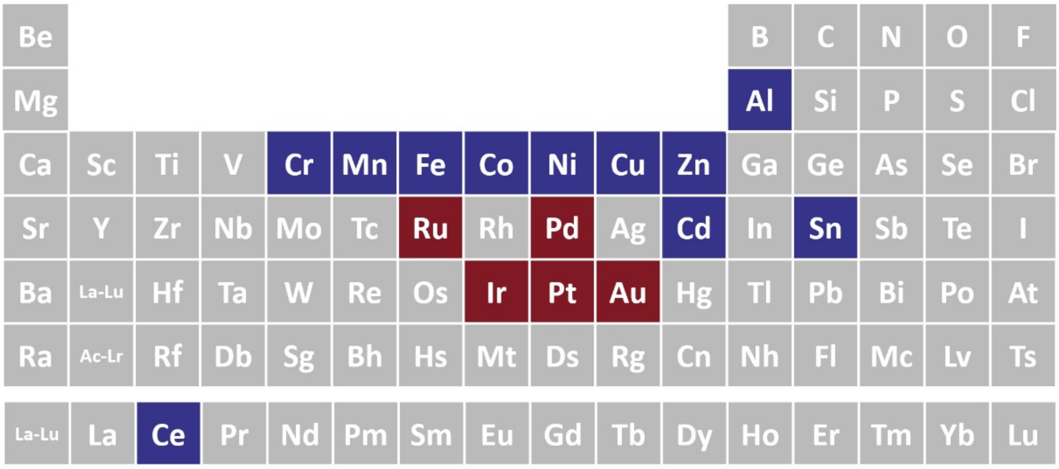


b

| | | |
|---|---|---|
| LEAs (9) | 9 Binary | PtFe, PtCo, PtNi, PtCu, RuSn, PdNi, PdCd, AuNi, IrCo, |
| MEAS (4) | 3 Ternary | PtNiFe, PtNiCo, PtFeCu, |
| | 1 Quaternary | PtRuNiFe |
| HEAs (8) | 1 Quinary | PtRuNiCoFe, |
| | 1 Senary | PtRuNiCoFeCd, |
| | 1 Septenary | PtRuNiCoFeCdMn |
| | 1 Octonary | PtRuNiFeCoCdCuCe |
| | 1 Novenary | PtRuNiFeCoCdMnAlCu |
| | 1 Denary | IrAuNiFeCoMnCrCuZnCd, |
| | 1 11- element | PtRuNiCoFeCdMnCrAlCuZn |
| | 1 12- element | PtRuNiCoFeCdMnCrAlCuZnCe |

**Fig. S7** (a) Periodic table in which Pt, Ru, Pd, Au, or Ir, (highlighted in crimson) and other metal elements (highlighted in light blue) that can form small-sized alloys on HCN. (b) Overview of the synthesized 21 supported nanoalloys.

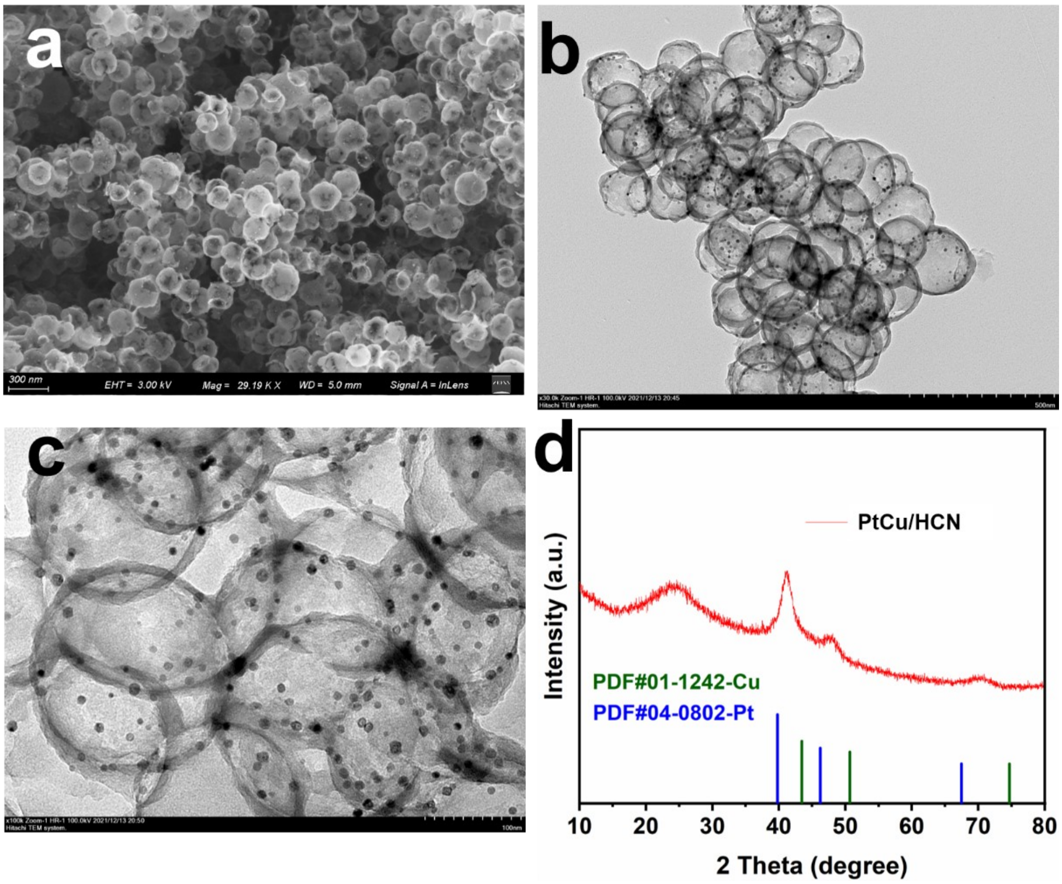


**Fig. S8** (a) SEM image, (b, c) TEM images and (d) XRD patterns of the prepared PtCu/HCN. (d) shows the diffraction peak positions of PtCu/HCN sample are lied between standard Pt (blue line) and standard Cu PDF card (dark green line), suggesting the formation of PtCu nanoalloy.

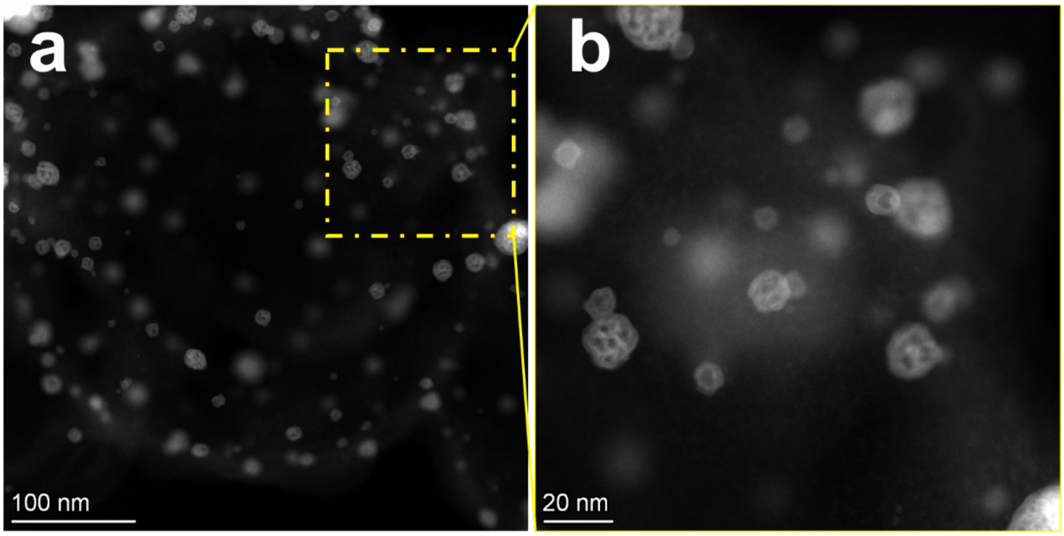


**Fig. S9** (a) HAADF-STEM image of PtCu/ HCN catalyst, and (b) the corresponding enlarged image of area in (a). It clearly shows the frame-work structure of PtCu nanoalloy, and distributed on the carrier surface uniformly.

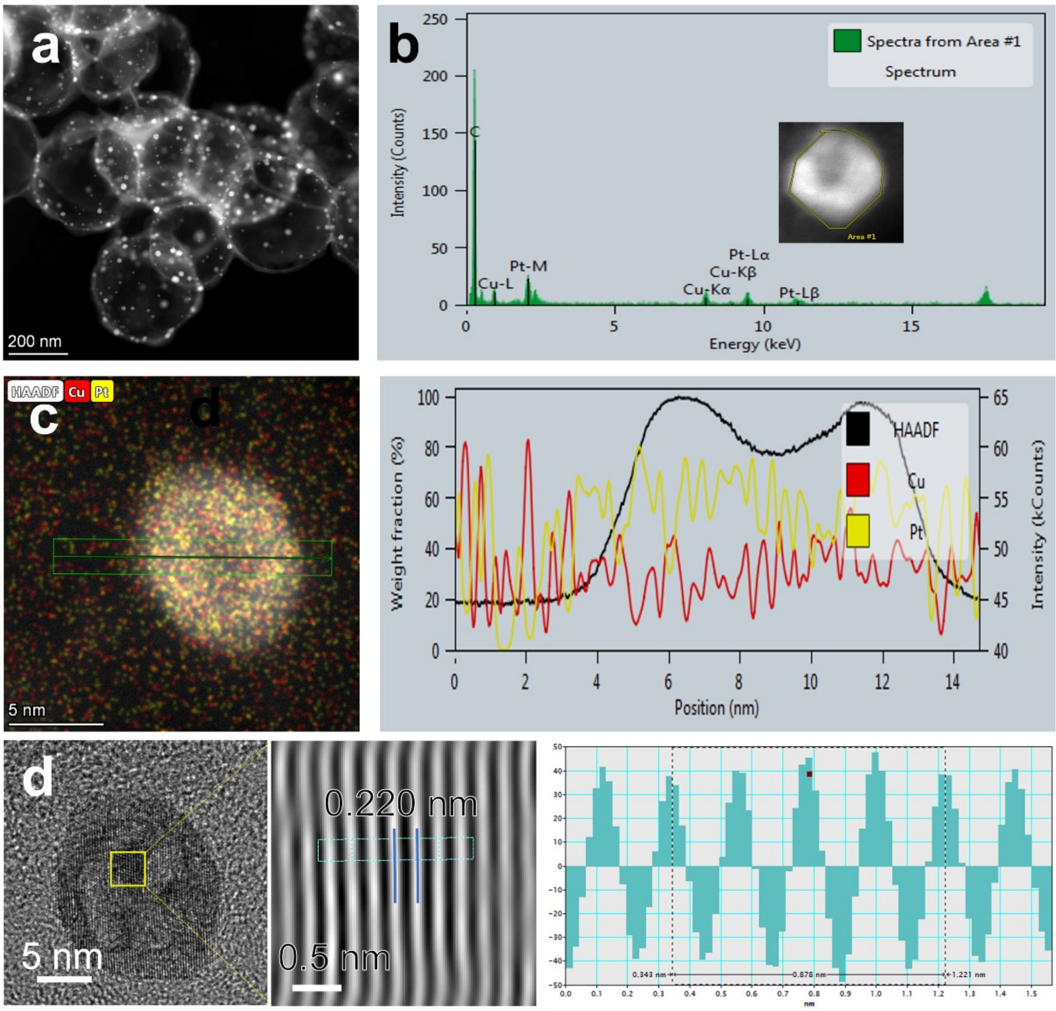


**Fig. S10** (a) HAADF-STEM image, (b) EDX spectrum, (c) EDX line scanning, (d) HRTEM image and inverse fast Fourier transformation (IFFT) image of area in (d) of PtCu/HCN samples. They demonstrate the uniform distribution of each elemental (Pt and Cu) as well as the formation of alloyed structures.

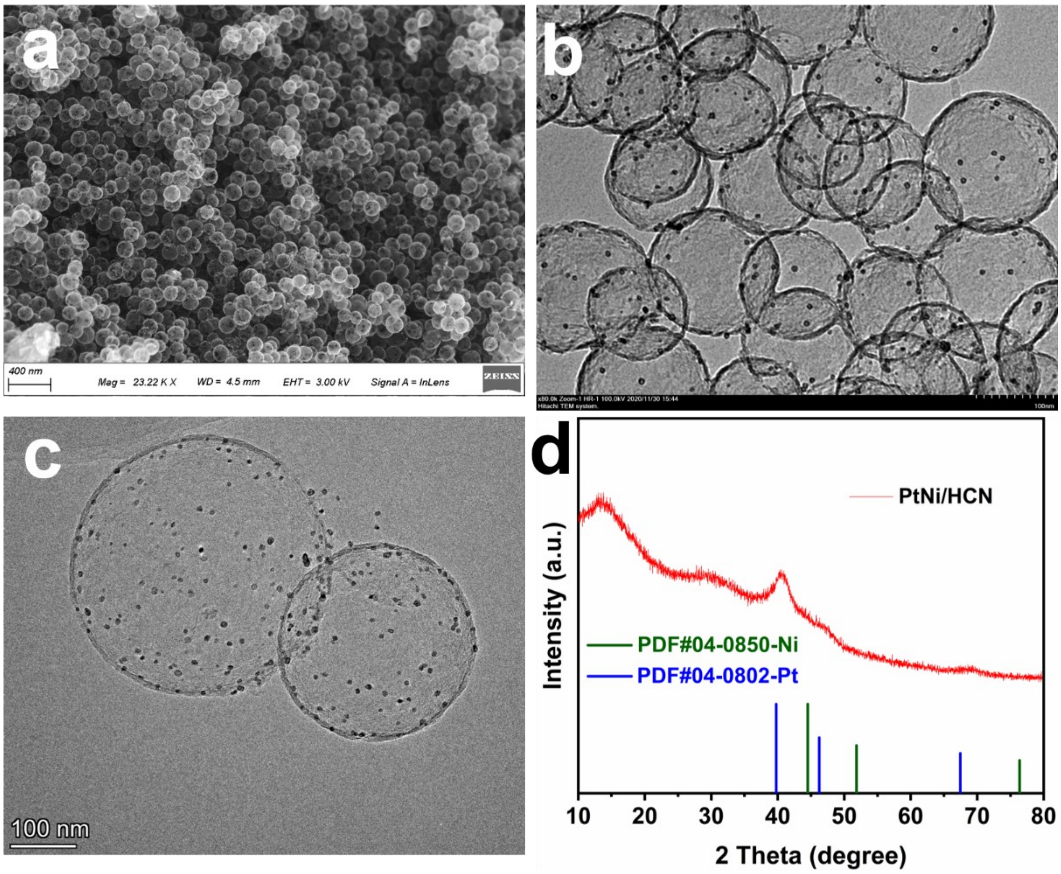


**Fig. S11** (a) SEM image, (b, c) TEM images and (d) XRD patterns of the prepared PtNi/HCN. (d) shows the diffraction peak positions of PtNi/HCN sample are lied between standard Pt (blue line) and standard Ni PDF card (dark green line), which confirms the PtNi alloyed formation.

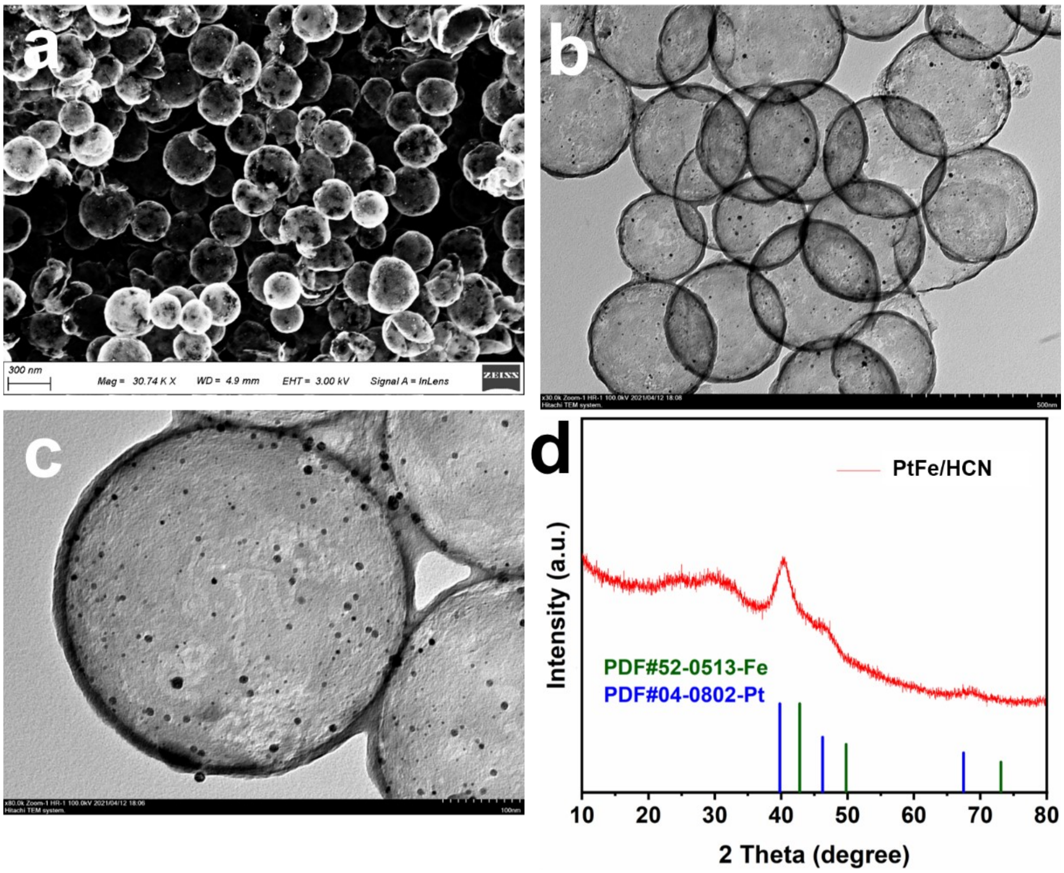


**Fig. S12** (a) SEM image, (b, c) TEM images and (d) XRD patterns of the prepared PtFe/HCN. (d) shows the diffraction peak positions of PtFe/HCN sample are lied between standard Pt (blue line) and standard Fe PDF card (dark green line), suggesting the formation of PtFe nanoalloy.

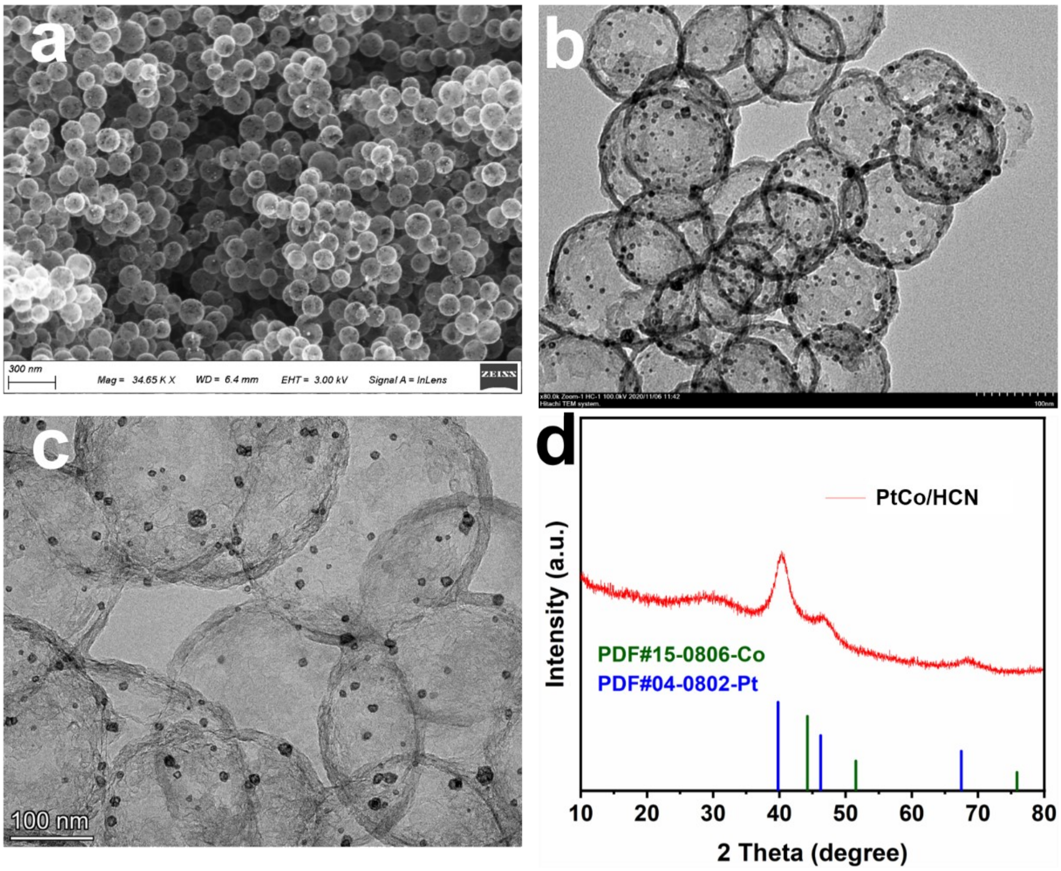


**Fig. S13** (a) SEM image, (b, c) TEM images and (d) XRD patterns of the prepared PtCo/HCN. (d) shows the diffraction peak positions of PtCo/HCN sample are lied between standard Pt (blue line) and standard Co PDF card (dark green line) and thus, PtCo nanoalloy is successfully achieved.

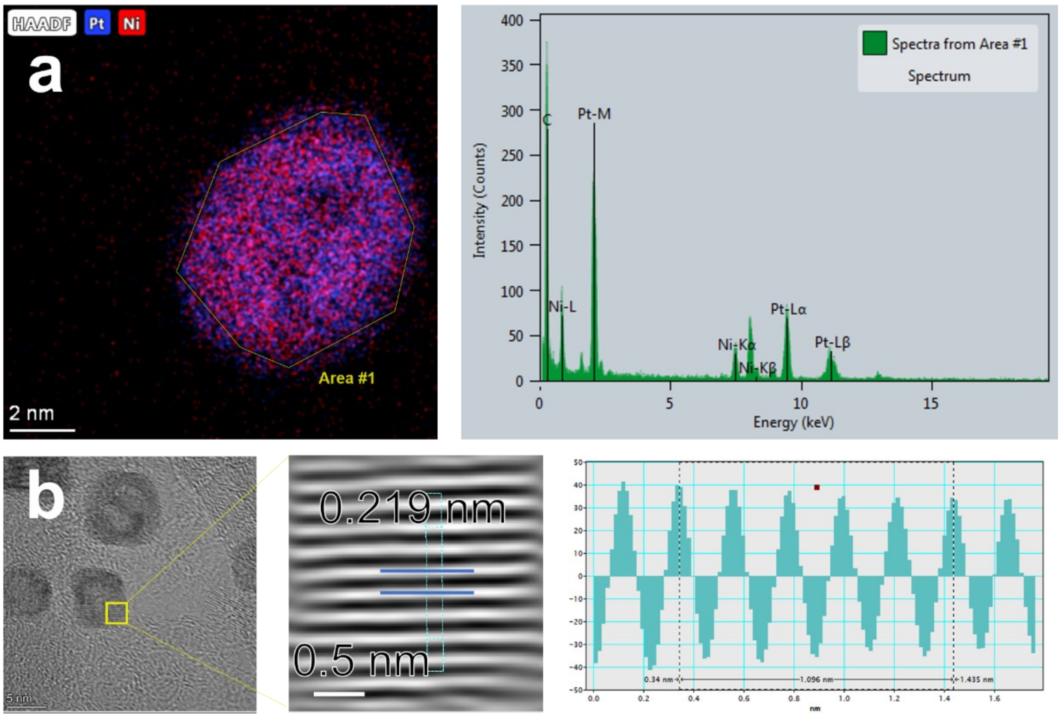


**Fig. S14** (a) EDX elemental mapping and corresponding EDX spectrum, (b) HRTEM image and IFFT image of area in (b) of PtNi/HCN samples. The nanoparticles show frame-work structure, and there is a uniform distribution of elements throughout the nanoparticles, which confirms PtNi alloyed formation.

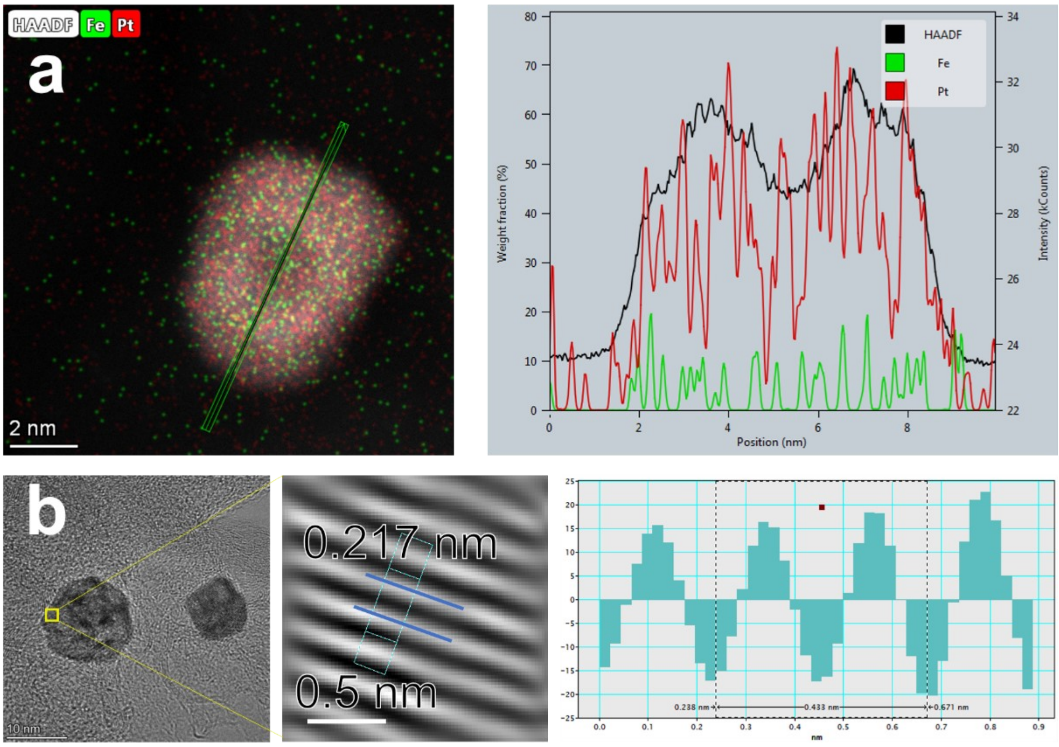


**Fig. S15** (a) EDX elemental mapping and corresponding EDX line scanning, (b) HRTEM image and IFFT image of area in (b) of PtFe/HCN samples. The frame-work structure can be clearly observed and nanoparticles show an even elemental (Pt and Fe) distribution as well as the formation of PtFe nanoalloy.

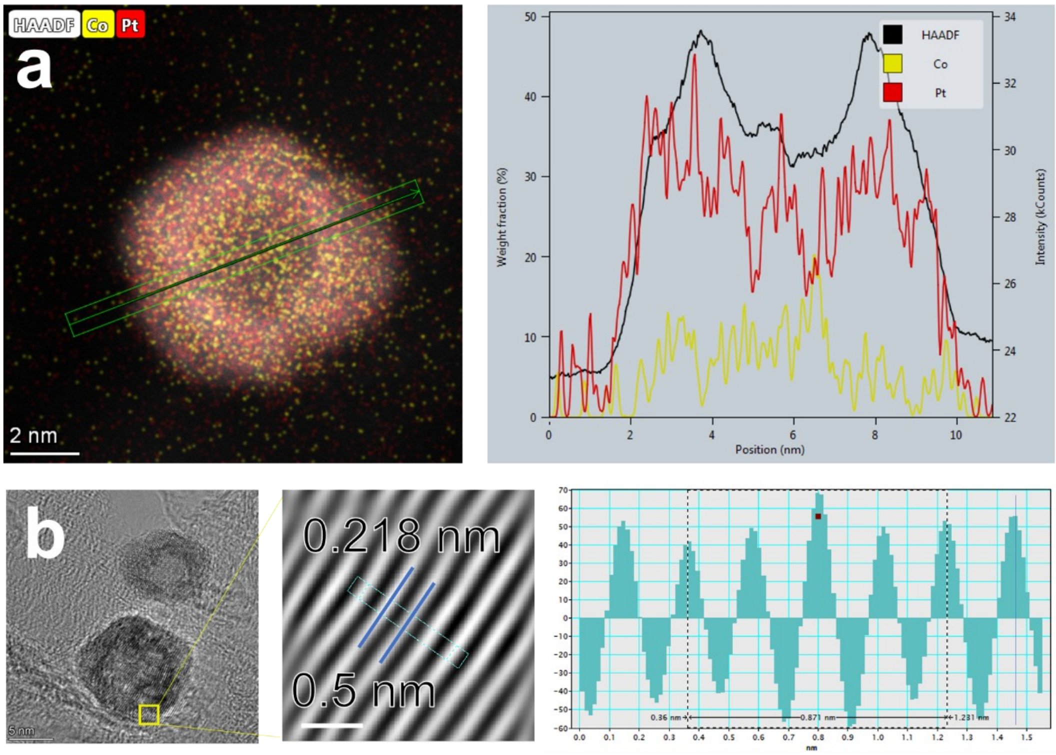


**Fig. S16** (a) EDX elemental mapping and corresponding EDX line scanning, (b) HRTEM image and IFFT image of area in (b) of PtCo/HCN samples. The nano-frame structure of nanoparticles at a glance, and PtCo alloyed formation as well as its compositional uniformity can be observed.

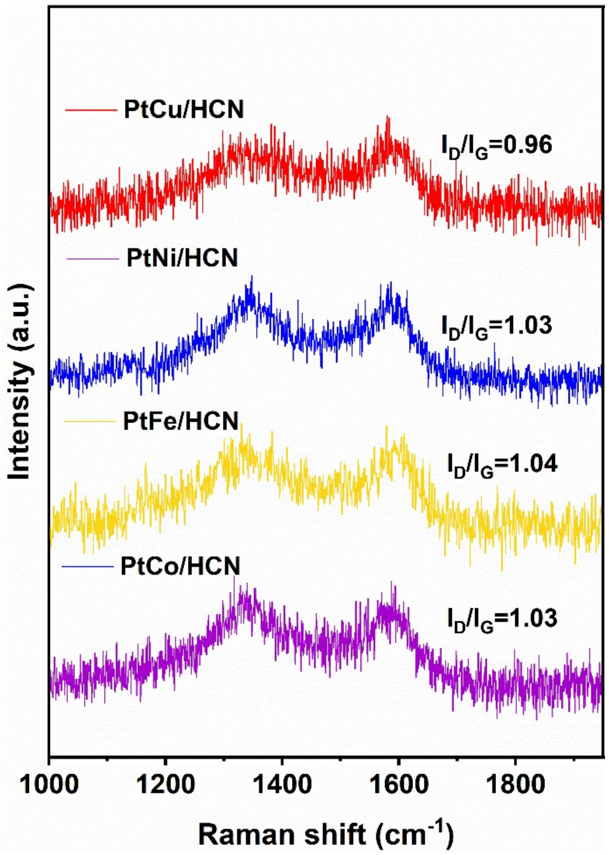


**Fig. S17** Raman spectra of the prepared PtM/HCN (M= Cu, Ni, Fe, Co). Raman spectra of PtCu/HCN and other three references all show two distinct peaks at 1333 $cm^{-1}$ (D band) and 1587 $cm^{-1}$ (G band) associated with disordered carbon and graphic carbon, respectively

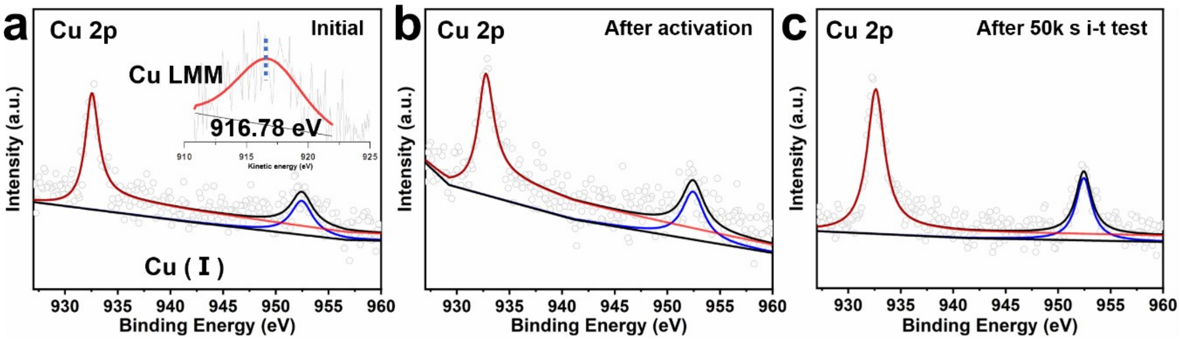


**Fig. S18** Cu 2p XPS spectra of (a) initial PtCu/HCN (b) PtCu/HCN after activation 50 cycles between 0 and 1.2 V vs. Ag/AgCl in the solution of 0.1 M $HClO_4$ + 1 M $CH_3OH$, and (c) PtCu/HCN after long-term durability test for 50k s of MOR at 0.7 V (vs. Ag/AgCl). And corresponding (inset) Cu LMM Auger spectrum.

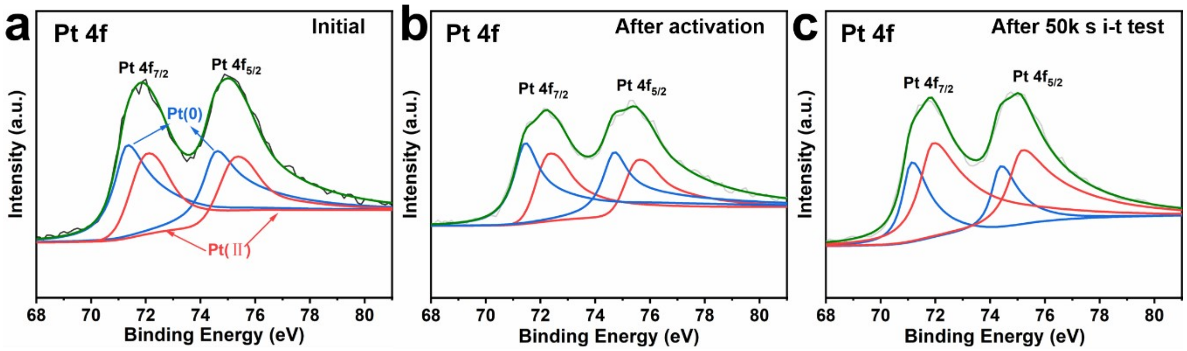


**Fig. S19** Pt 4f XPS spectra of (a) initial PtCu/HCN, (b) PtCu/HCN after activation 50 cycles between 0 and 1.2 V (vs. Ag/AgCl) in the solution of 0.1 M $HClO_4$ + 1 M $CH_3OH$, and (c) PtCu/HCN after long-term durability test for 50k s of MOR at 0.7 V (vs. Ag/AgCl). They demonstrate that the content of high valence Pt (II) increases compared to the PtCu/HCN after long-term stability test for 50k s, while the low valence Pt (0) is opposite.

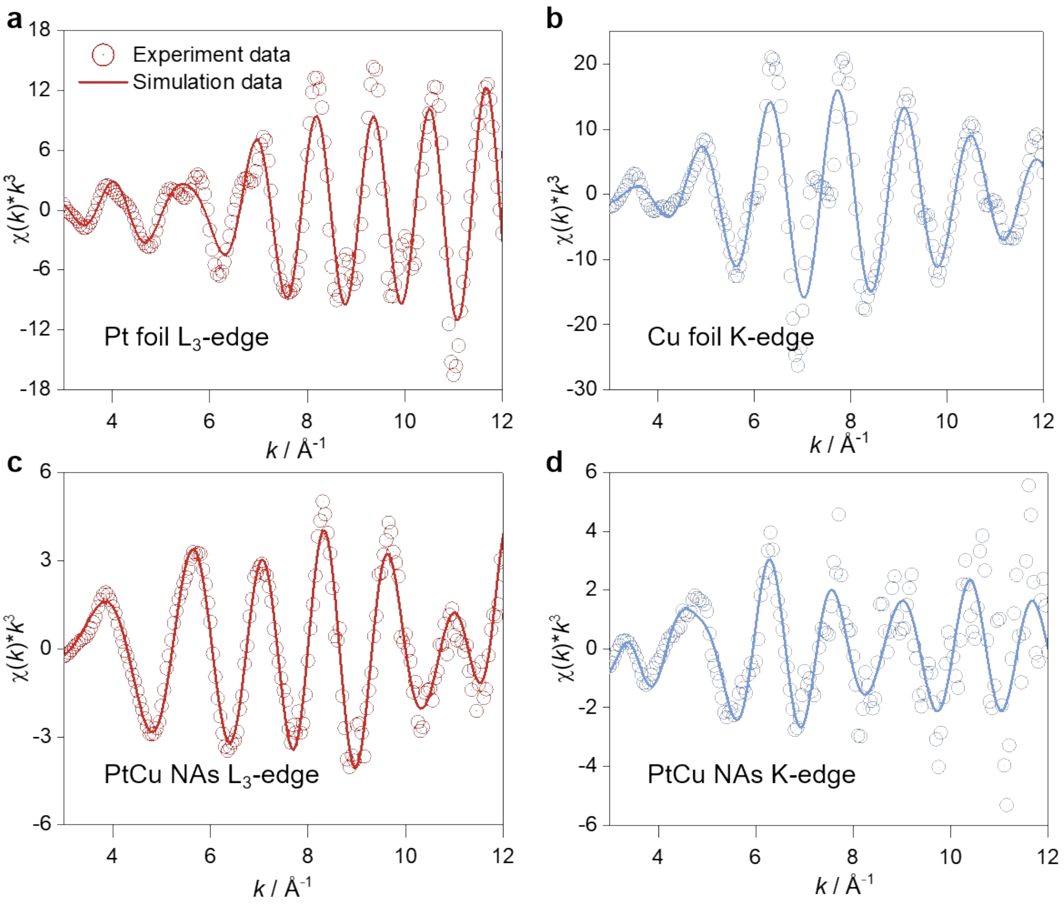


**Fig. S20** Fitting results of Pt foil, Cu-foil and PtCu/HCN at Pt $L_3$-edge and Cu K-edge. (a) Pt foil, (b) Cu-foil, (c, d) PtCu/HCN about the $k^3$-weighted $k$ space fitting curves.

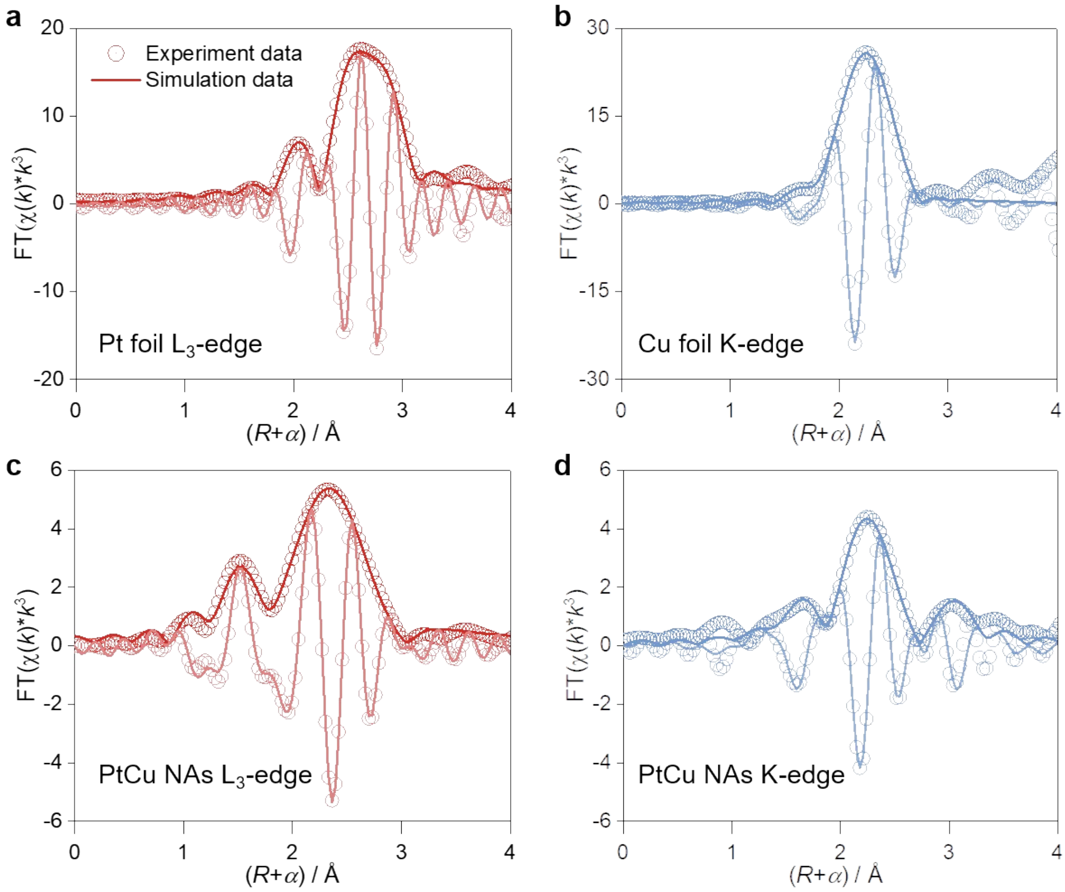


**Fig. S21** Fitting results of Pt foil, Cu-foil and PtCu/HCN at Pt $L_3$-edge and Cu K-edge. (a) Pt foil, (b) Cu-foil, (c, d) PtCu/HCN: the R-space (FT magnitude and imaginary component). The data are $k^3$-weighted and not phase-corrected.

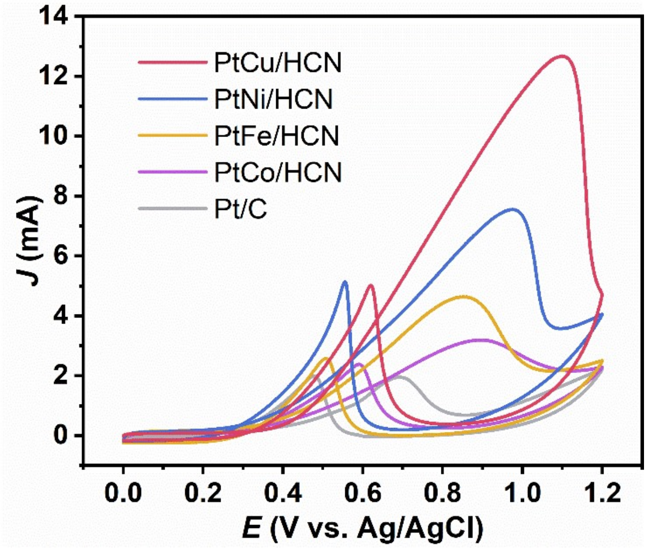


**Fig. S22** Electrocatalytic activity of PtM/HCN (M=Cu, Ni, Fe, Co) and Pt/C catalysts in 0.1 M $HClO_4$ +1 M $CH_3OH$ at a scanning rate of 50 mV $s^{-1}$. Catalyst loadings on the electrode are 0.24 $mg_{cat}$ $cm^{-2}$.

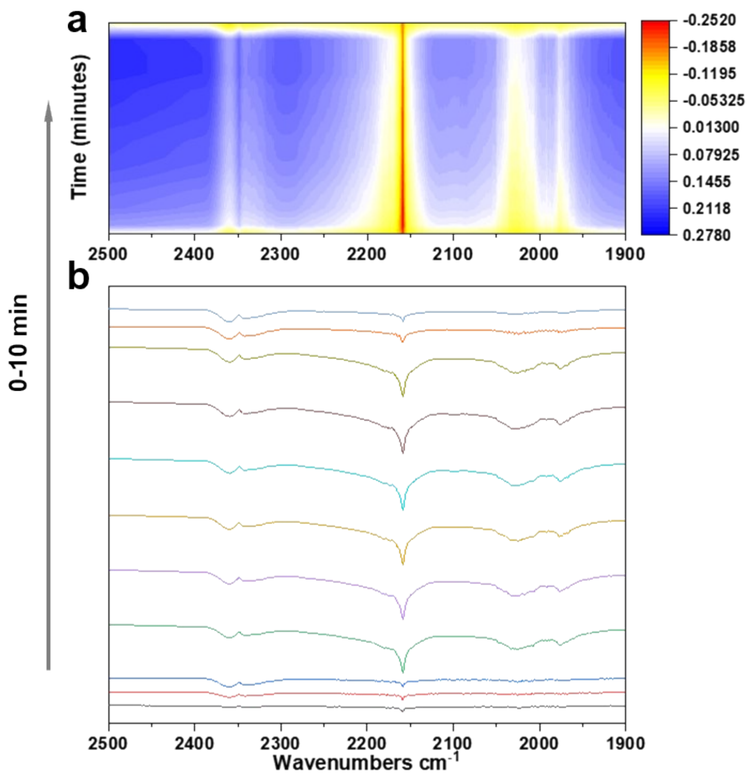


**Fig. S23** (a) counter plot and (b) spectra for in-situ IR recorded during electrocatalytic MOR of PtCu/HCN from 0-10 minutes.

In situ electrochemical infrared spectroscopy was used to study electrocatalytic oxidation behavior of methanol on PtCu/HCN catalyst, Fig. S23 shows the in-situ IR spectra of MOR process during 0-10 min from potential range of 0-1.2 V (RHE), we can see a negative spectral peak gradually appears at 2028 $cm^{-1}$ as the reaction time increases, which is attribute to the infrared absorption peak of linear CO ($CO_L$) as a toxic intermediate product of $CH_3OH$. In the early stage of the reaction, methanol molecules were oxidized to intermediate CO, but with the increase of reaction time, the intensity of the CO spectral peak gradually decreased after 3 min, indicating that the number of CO intermediates gradually decreased, and the fast desorption of CO intermediates on catalysts, manifesting the strong CO tolerance. In addition, the positive spectral peak observed at 2350 $cm^{-1}$ is ascribed to the asymmetric stretching vibration for $CO_2$, and a tendency of gradually increased represents the $CO_2$ molecules increasing existed in the electrolyte, indicating an enhancement of direct conversions for methanol molecules, which is in agreement with the decrease of CO.

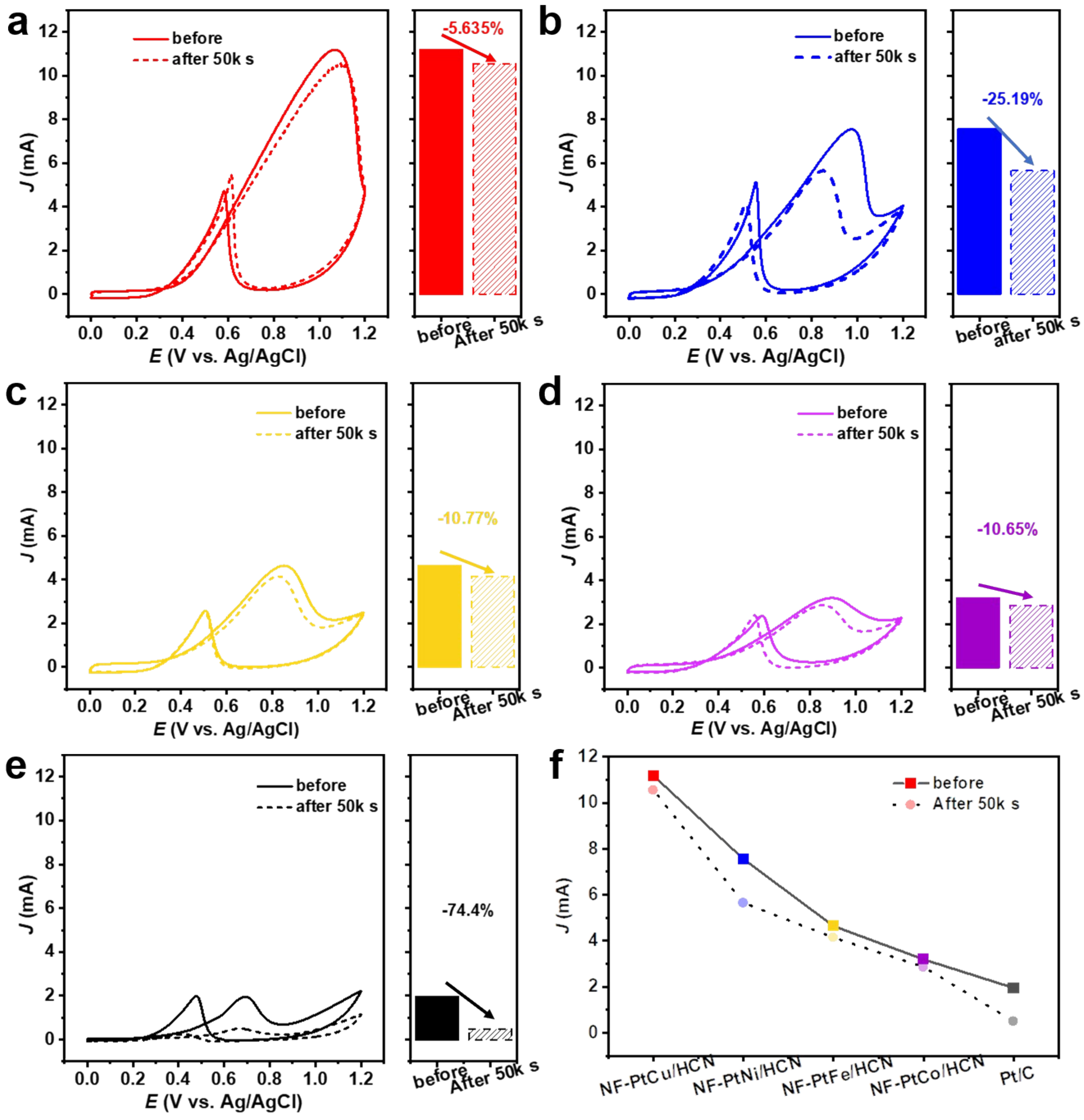


**Fig. S24** Electrochemical durability of PtCu/HCN sample and its counterpart catalysts. LSV curves and bar graphs for the peak value of prepared (a) PtCu/HCN, (b) PtNi/HCN, (c) PtFe/HCN, (d) PtCo/HCN, and (e) commercial Pt/C catalysts before (solid lines) and after (dash lines) long-term durability test for 50k s of MOR at 0.7 V (vs. Ag/AgCl). The LSV test was operated in 0.1 M $HClO_4$ +1 M $CH_3OH$ at a scanning rate of 50 mV $s^{-1}$. The peak changes and contrasts are summarized in Fig. S24f.

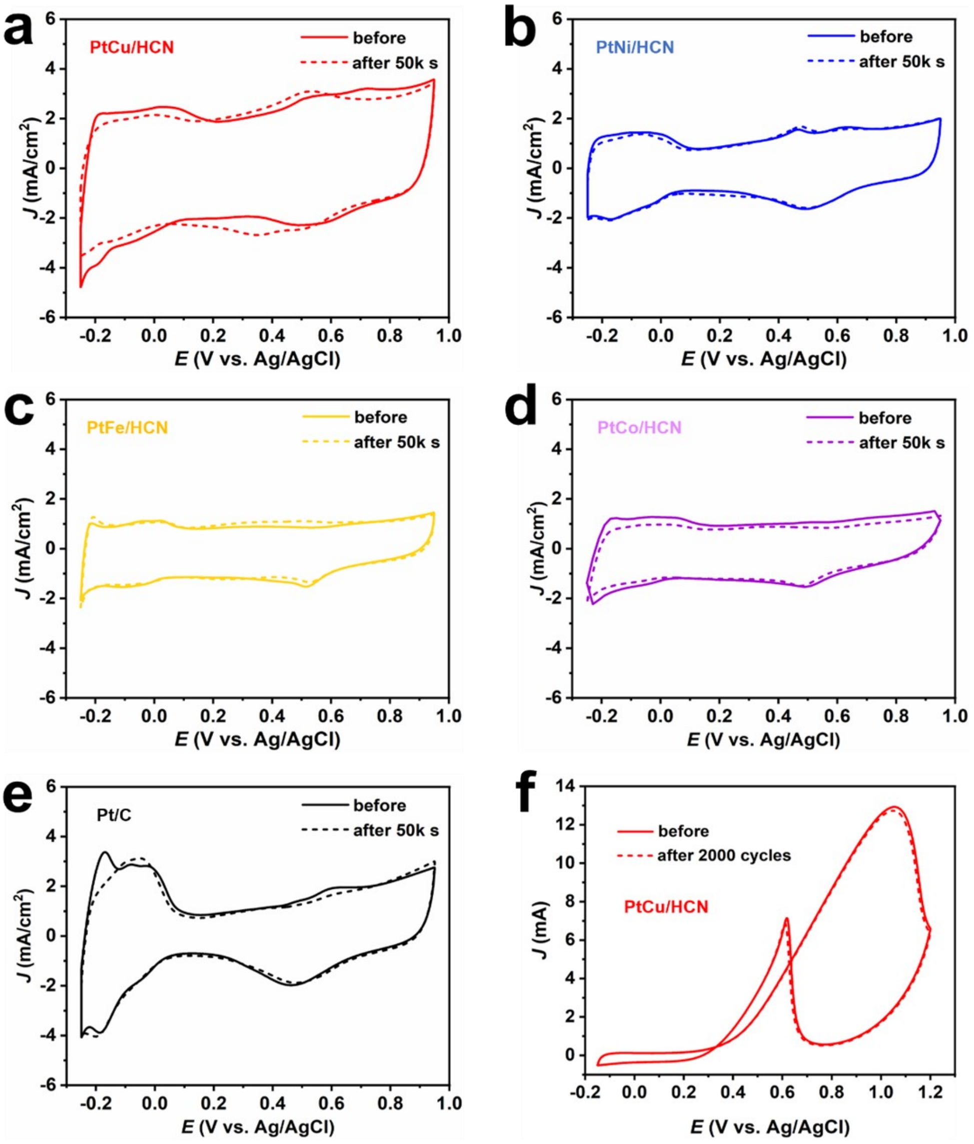


**Fig. S25** CV curves of (a) PtCu/HCN, (b) PtNi/HCN, (c) PtFe/HCN, (d) PtCo/HCN and (e) commercial Pt/C catalysts before (solid lines) and after (dash lines) long-term durability test for 50k s of MOR at 0.7 V (vs. Ag/AgCl). The measurement of CV curves was carried out in a $N_2$-saturated 0.1 M $HClO_4$ solution at a sweep rate of 50 mV $s^{-1}$. (f) CV curves before and after 2000 potential cycles between -0.15 and 0.65 V vs Ag/Ag/Cl in 0.1 M $HClO_4$ + 1 M $CH_3OH$ at a sweep rate of 50 mV/s.

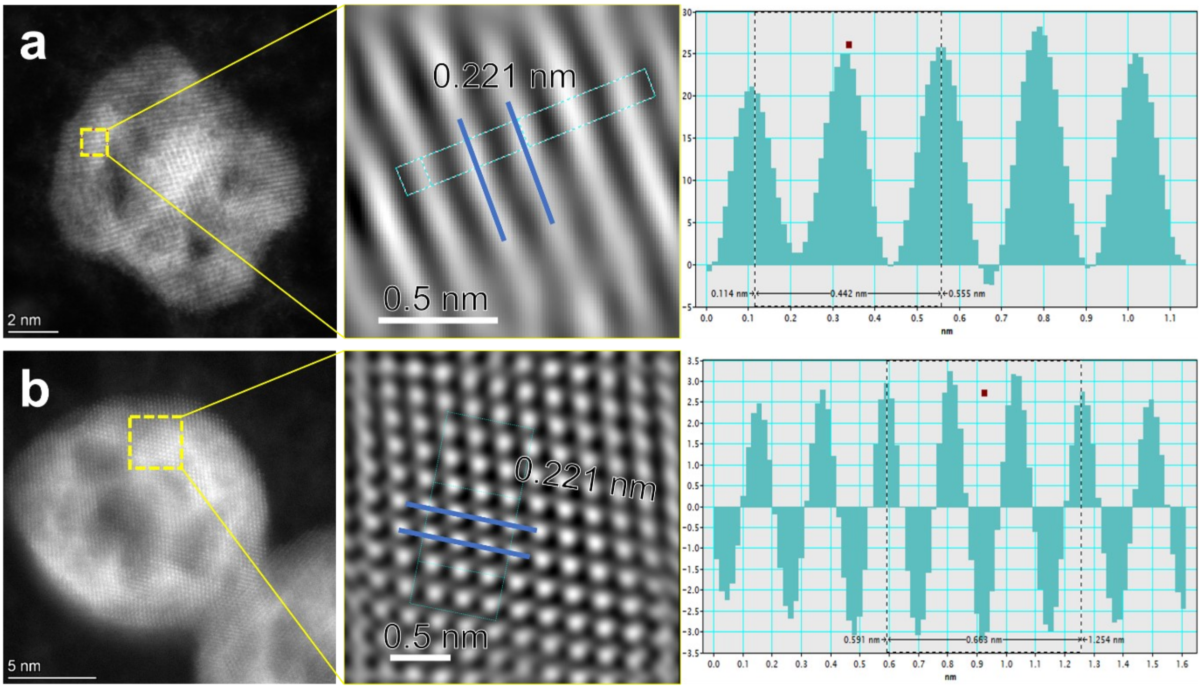


**Fig. S26** HAADF-STEM images, IFFT images of area in the yellow box as well as lattice spacing for PtCu/HCN catalyst (a) before and (b) after long-term durability test for 50k s of MOR at 0.7 V (vs. Ag/AgCl), respectively. The result shows PtCu nanoalloy retains original structure, and the lattice spacing of surface nanoparticles have no change, which demonstrates the structural and compositive stability of PtCu/HCN samples undergoing MOR catalytic reaction for 50k seconds.

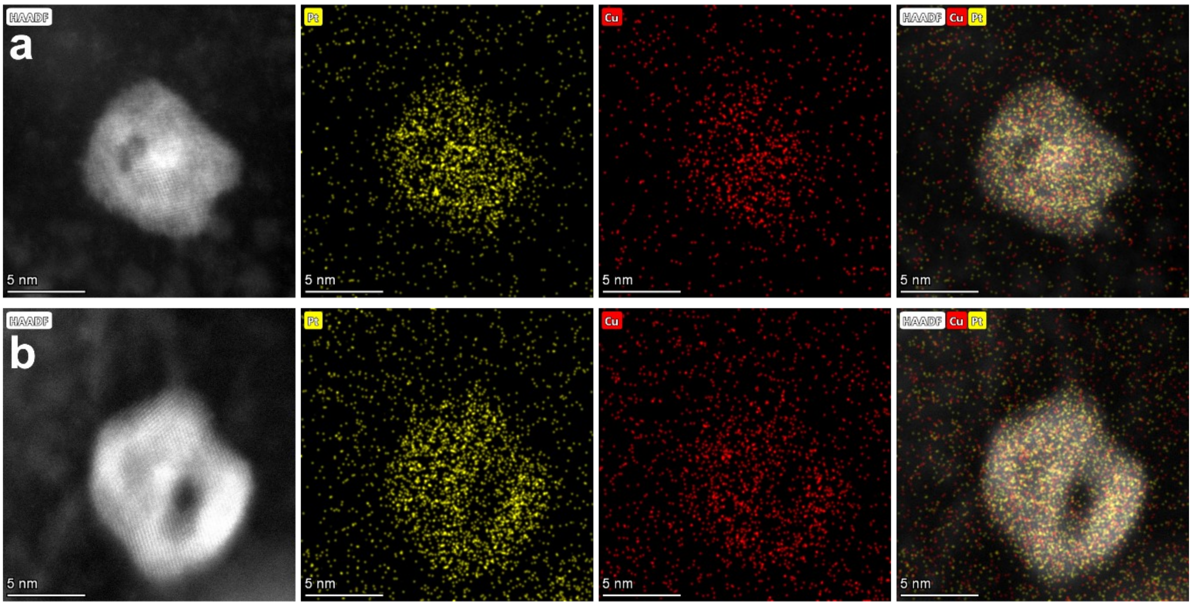


**Fig. S27** EDX elemental mappings of PtCu/HCN (a) before and (b) after long-term durability test for 50k s of MOR at 0.7 V (vs. Ag/AgCl). The frame-work structure and each elemental (Pt and Cu) distribution uniformity of PtCu nanoalloy remain stable undergoing the MOR for 50k s.

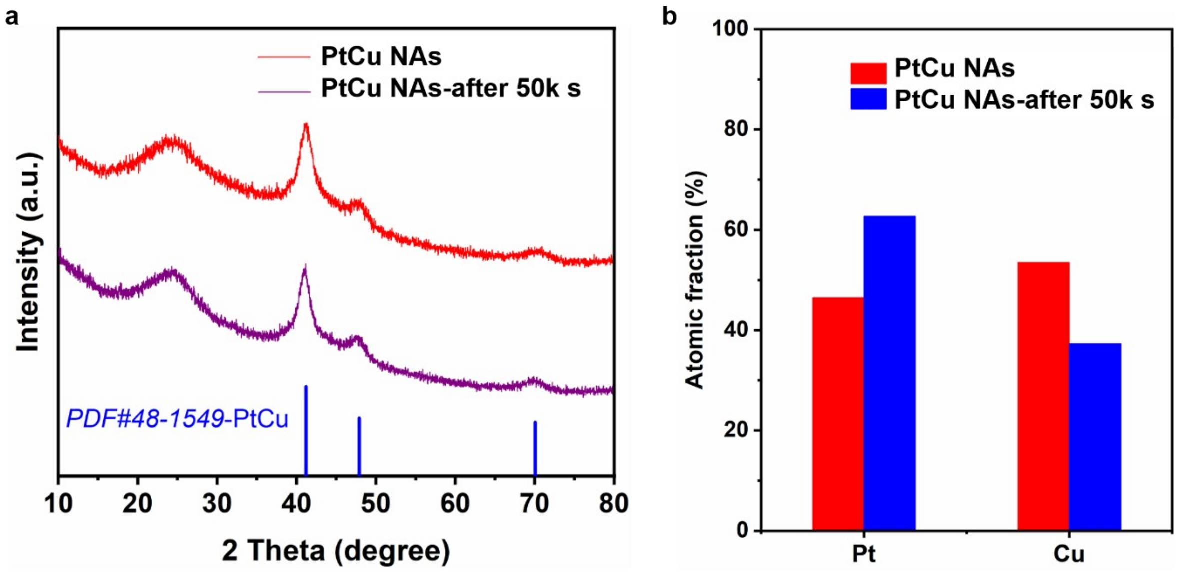


**Fig. S28** (a) XRD patterns and (b) ICP-MS analysis of PtCu/HCN before and after long-term durability test for 50k s of MOR at 0.7 V (vs. Ag/AgCl) in the solution of 0.1 M $HClO_4$ + 1 M $CH_3OH$. It manifests that the structure and composition have no change after the long-term durability test for 50k s, indicating the stability of prepared PtCu/HCN.

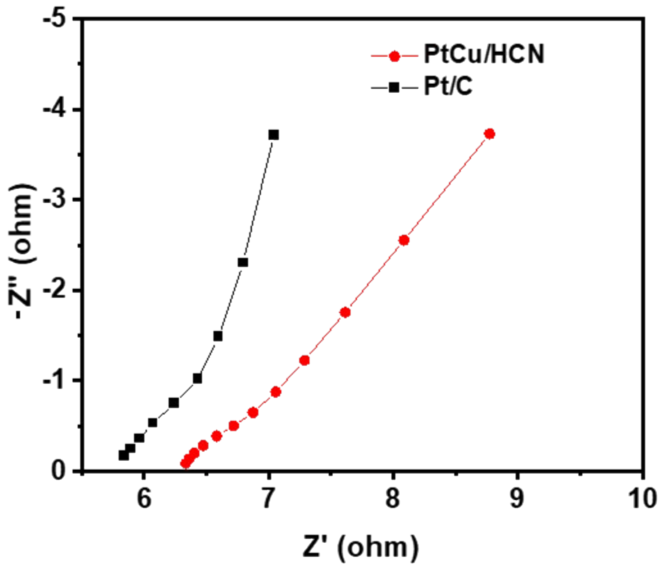


**Fig. S29** The Nyquist plots of PtCu/HCN and Pt/C in the frequency range of 0.1 to $10^5$ Hz. The EIS shows PtCu/HCN possesses a smaller diameter of semicircle, indicating the lower transfer resistance than Pt/C and manifesting the higher interfacial charge transfer, resulting in faster HER kinetics.

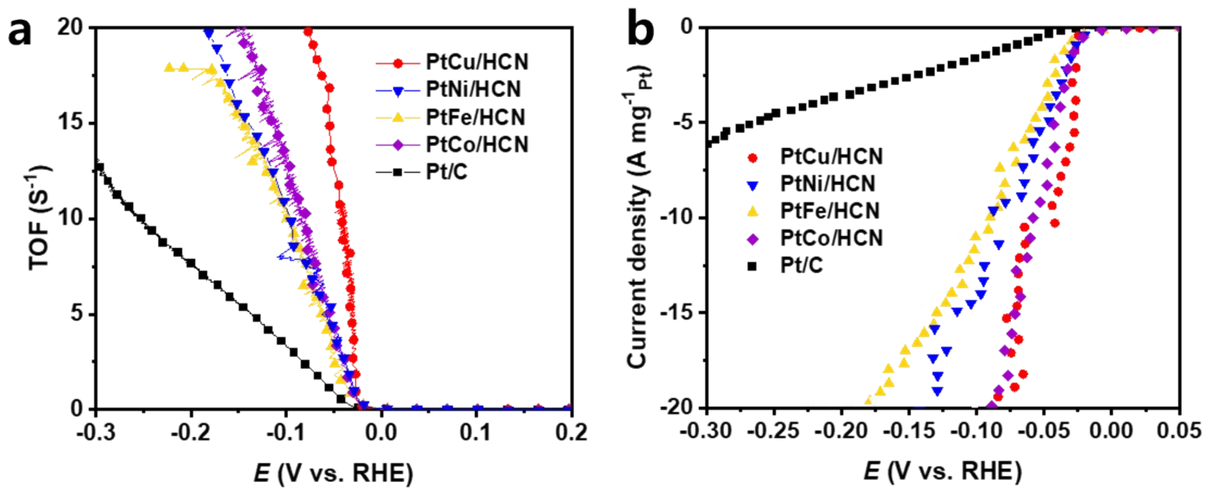


**Fig. S30** (a) Pt mass loading normalized (mass activity) LSV curves and (b) TOF of PtCu/HCN, PtNi/HCN, PtFe/HCN, PtCo/HCN, and commercial Pt/C in 0.5 M $H_2SO_4$ solution.

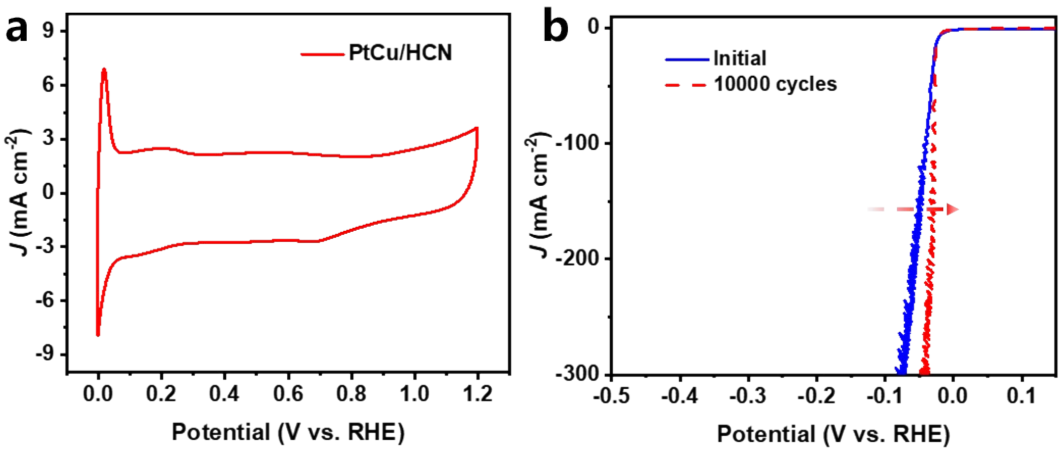


**Fig. S31** CV curves of PtCu/HCN catalysts in 0.5 M $H_2SO_4$, and HER polarization curves for PtCu/HCN before and after 10000 CV cycles.

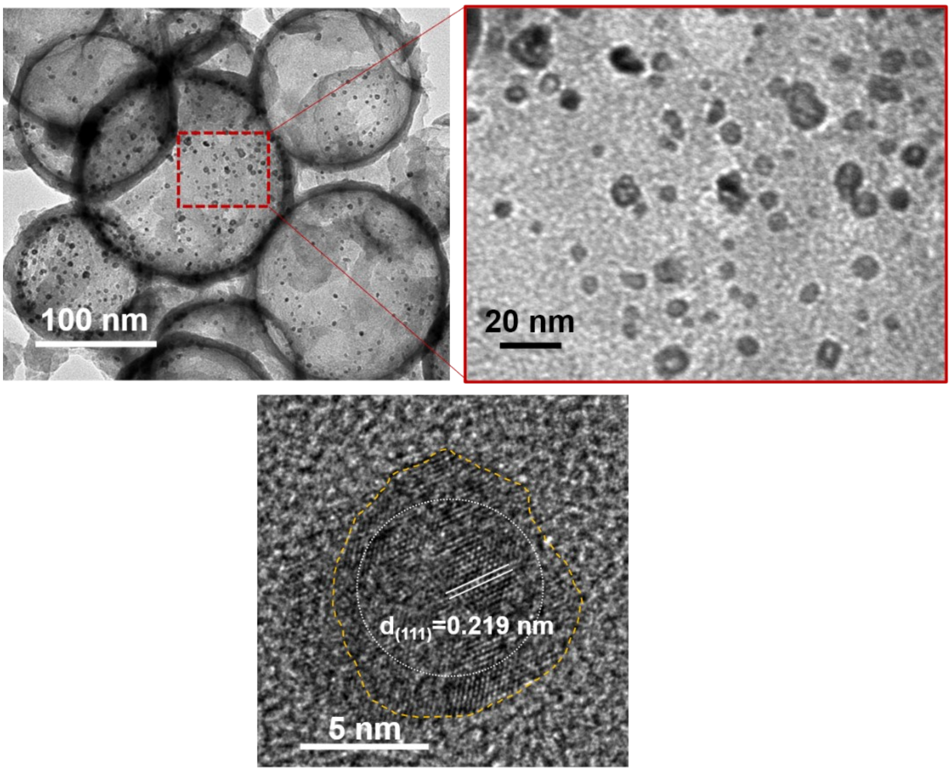


**Fig. S32** TEM images and STEM image of PtCu/HCN after long-term durability test for 150 h.

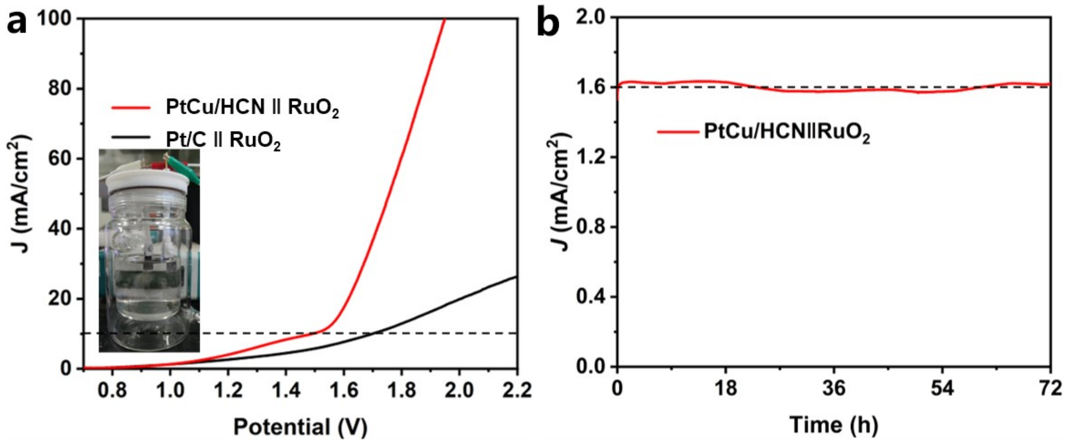


**Fig. S33** (a) LSV curves of electrochemical water splitting and (b) the Continuous potentiometric V–t measurement of PtCu/HCN ǁ $RuO_2$. The electrochemical water splitting tests in 0.5 M $H_2SO_4$ were carried out in a two-electrode system with the PtCu/HCN ǁ $RuO_2$ and Pt/C ǁ $RuO_2$ coupled catalysts. The catalysts were loaded on carbon cloth (0.5 M $H_2SO_4$) at a loading of 0.595 mg $cm^{-2}$. To assess the long-term water splitting stability of PtCu/HCN ǁ $RuO_2$, the Continuous potentiometric V–t measurement of PtCu/HCN ǁ $RuO_2$ was employed under a constant current density of 10 mA $cm^{-2}$ for 72 h.

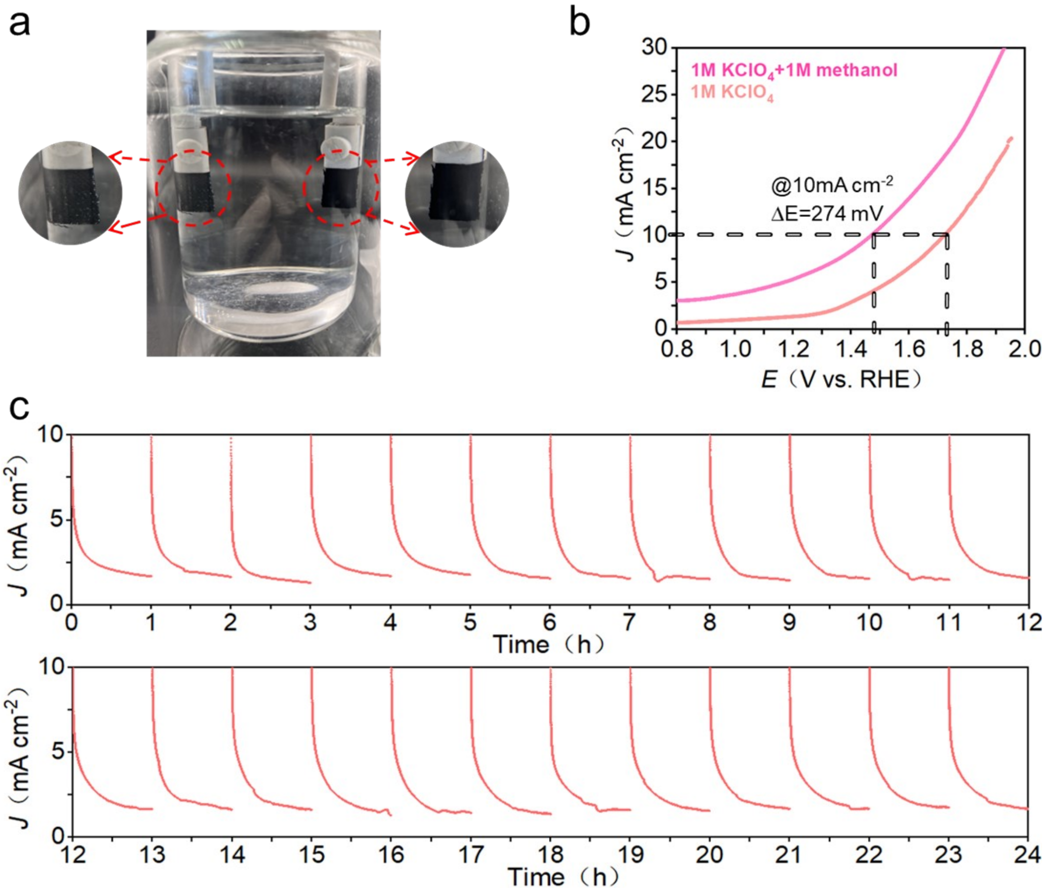


**Fig. S34** Schematic illustration of two-electrode system. (a) Photographs of the two-electrode configuration during the operation of the electrocatalytic system in the methanol-water electrolyzer (b) Comparison of the overall water splitting and methanol–water co-electrolysis by using PtCu/HCN, (c) the stability test of PtCu/HCN.

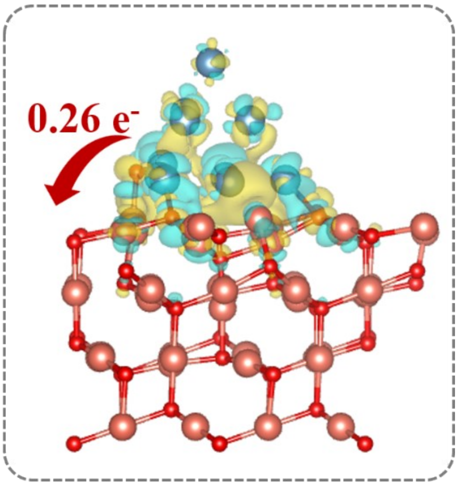


**Fig. S35** The electron density difference plot of $Cu_2O$/Pt. The arrow denotes the electronic transfer; The red, gold, and blue balls denote O, Cu, and Pt atoms, respectively. The yellow and blue regions denote the electronic accumulation and depletion, respectively.

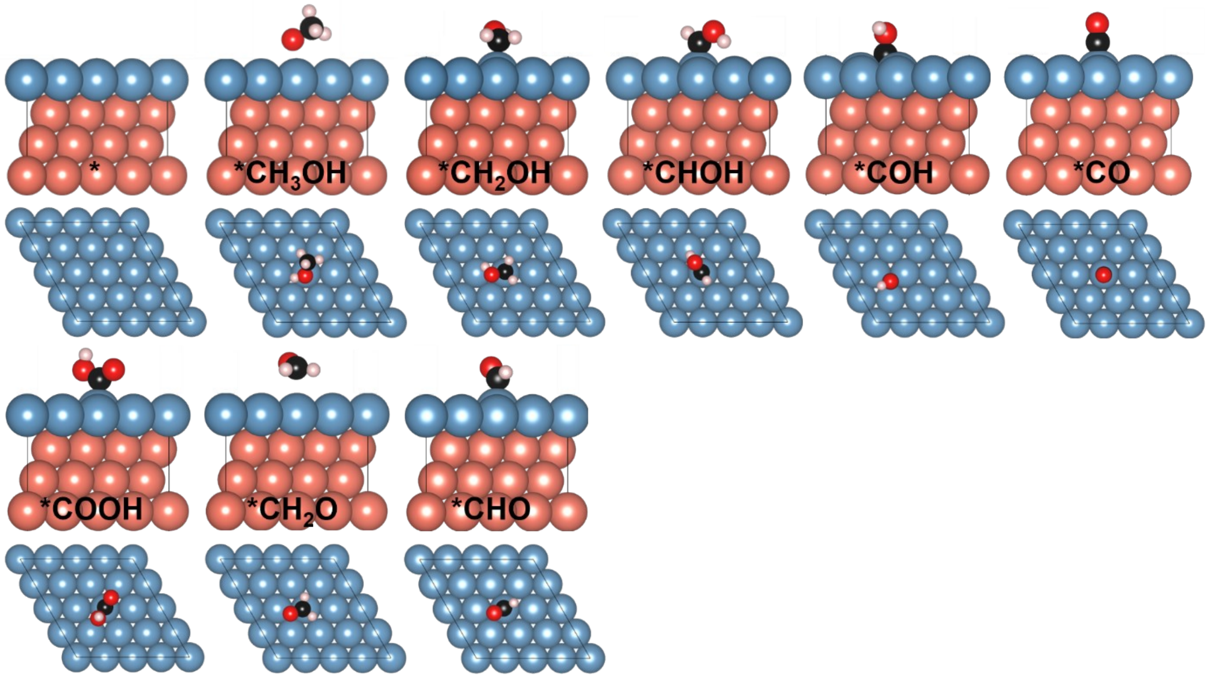


**Fig. S36** CuPt models and corresponding MOR intermediates, where the white, brown, red, indigo, and gray balls denote H, C, O, Cu, and Pt atoms, respectively.

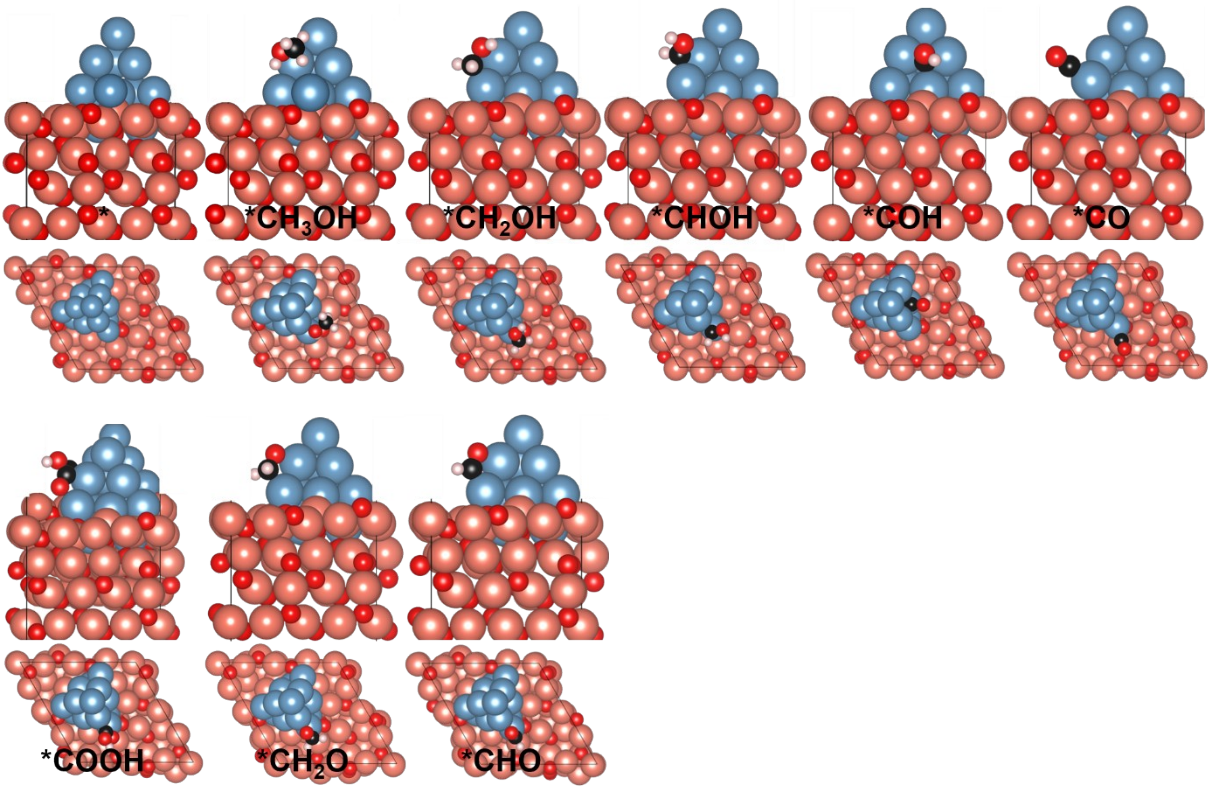


**Fig. S37** $Cu_2O$/Pt models and corresponding MOR intermediates, where the white, brown, red, indigo, and gray balls denote H, C, O, Cu, and Pt atoms, respectively.

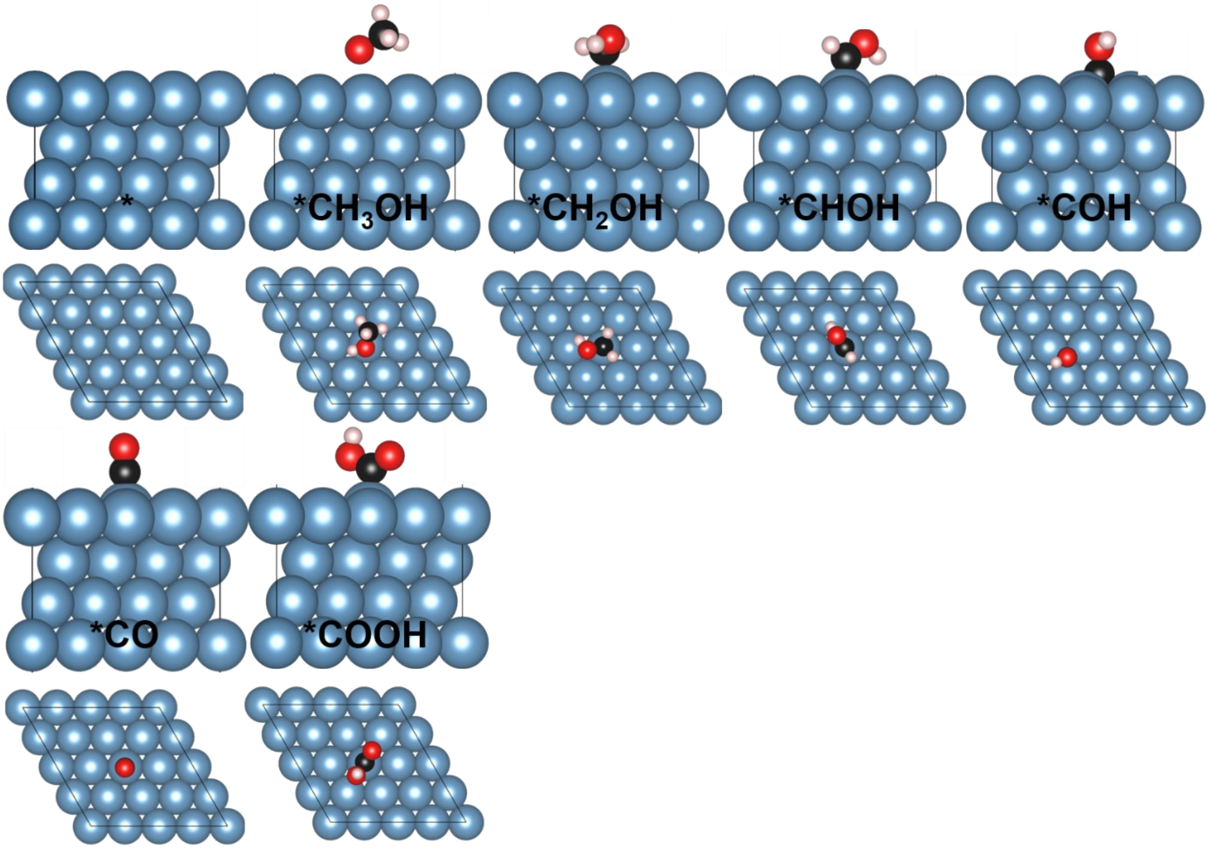


**Fig. S38** Pt (111) models and corresponding MOR intermediates where the white, brown, red, and gray balls denote H, C, O, and Pt atoms, respectively.

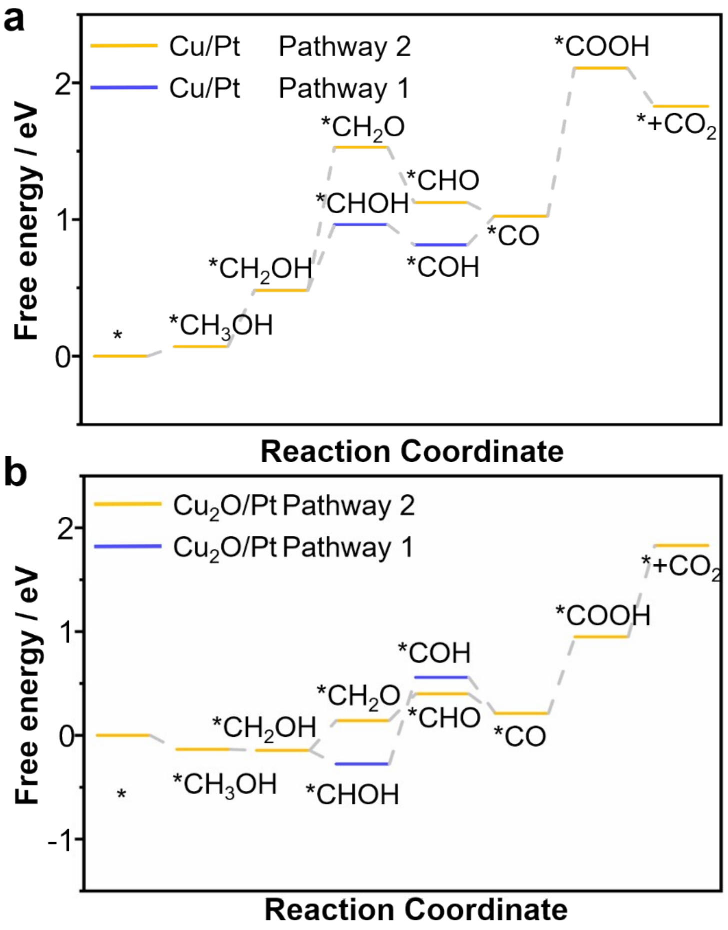


**Fig. S39** The free energy diagrams of methanol oxidation on (a) Cu/Pt and (b) $Cu_2O$/Pt.

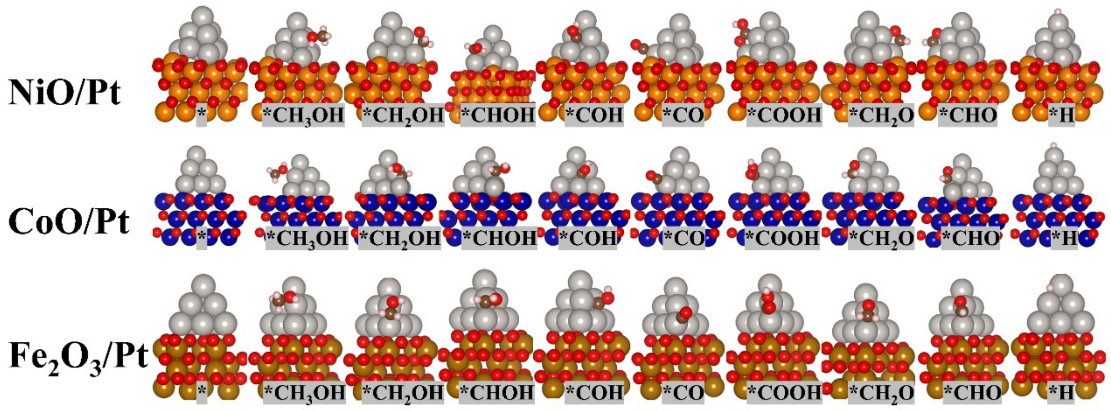


**Fig. S40** The adsorbed states of MOR and HER on NiO/Pt, CoO/Pt, and $Fe_2O_3$/Pt. The asterisk denotes the adsorbed sites. The white, brown, red, orange, blue, yellow, grey balls represent the H, C, O, Fe, Co, Ni, and Pt atoms, respectively.

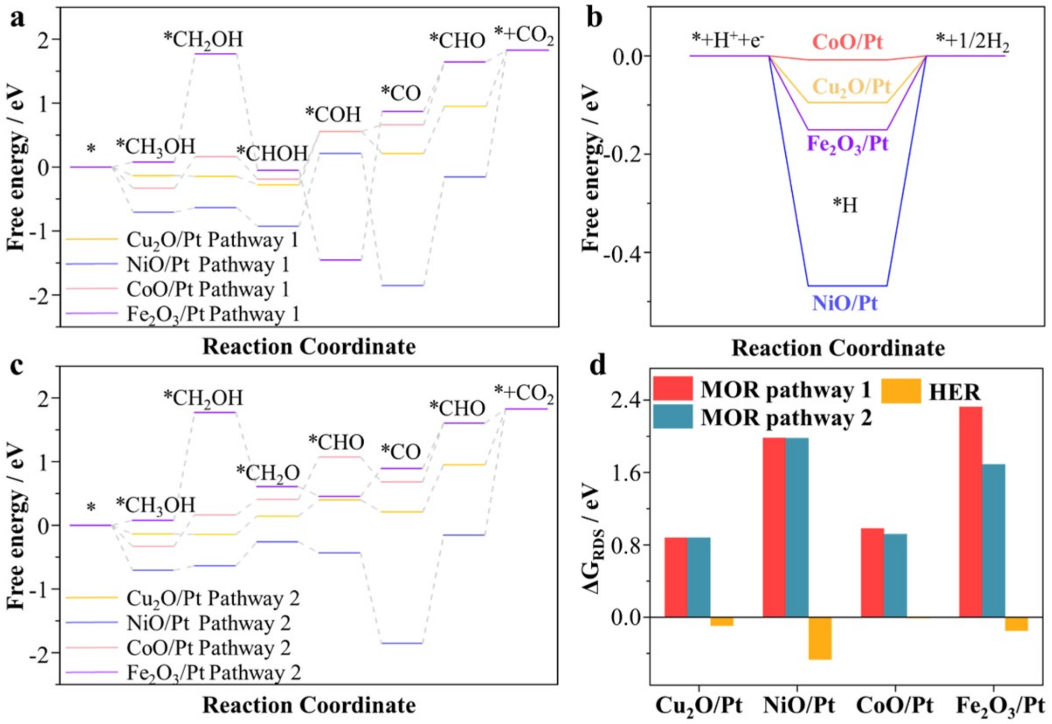


**Fig. S41** The free energy profiles of MOR along pathways 1 (a) and 2 (c) on $Cu_2O$/Pt, NiO/Pt, CoO/Pt, and $Fe_2O_3$/Pt. (b) The free energy profile of HER on $Cu_2O$/Pt, NiO/Pt, CoO/Pt, and $Fe_2O_3$/Pt. (d) The free energy changes of rate-determining step (RDS) of MOR and HER on $Cu_2O$/Pt, NiO/Pt, CoO/Pt, and $Fe_2O_3$/Pt.

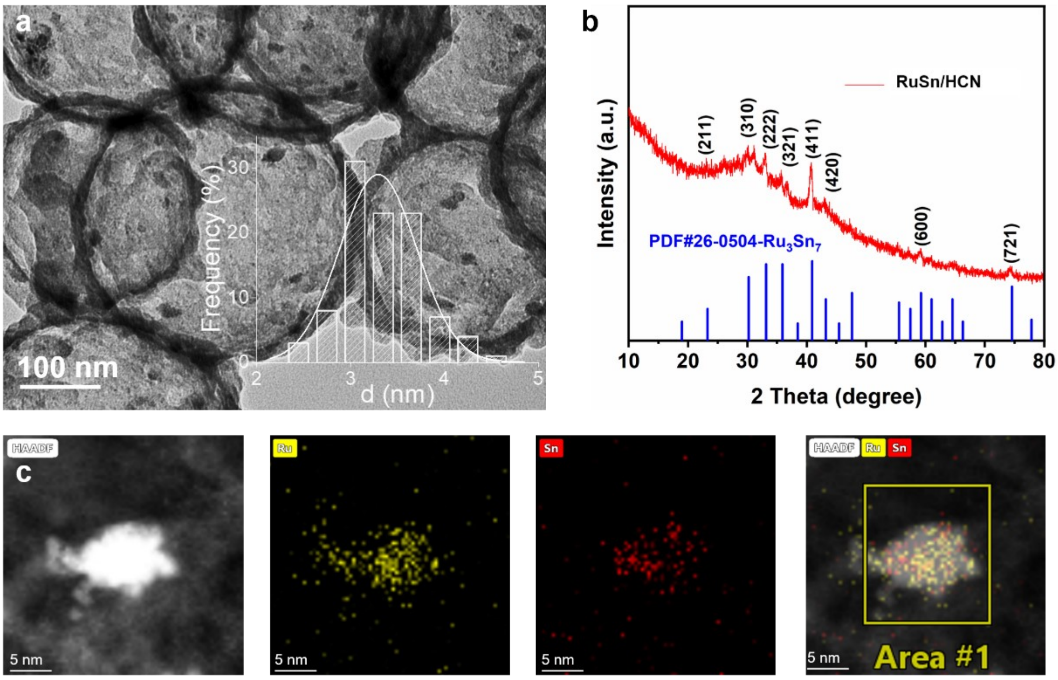


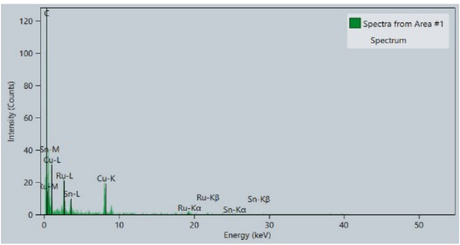


e

| Elemental | Atom% |
|---|---|
| Ru | 61.4 |
| Sn | 38.6 |

**Fig. S42** (a) TEM image and particle size distribution of RuSn/HCN. (b) XRD pattern of RuSn/HCN, which shows the single-phase and no impurity, has no significant diffraction peak is detected. (c) STEM-EDS mappings, (d) EDS spectrum and (e) atomic ratio of the selected RuSn particles. The reason why the absence of distinct alloy diffraction peaks maybe from the low content or ultrasmall size of the crystal structure, which agrees with a previous study on an ultrasmall high entropy alloy nanocrystal.

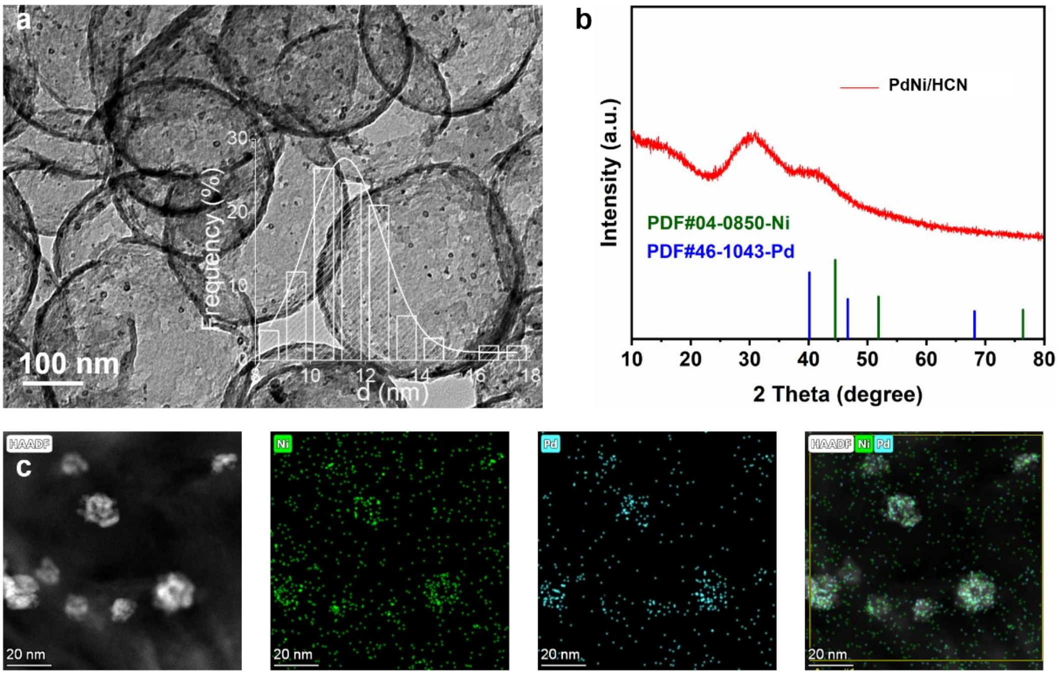


d

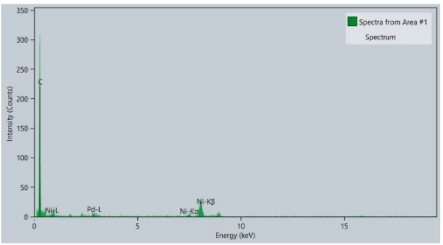

e

| Elemental | Atom% |
|---|---|
| Pd | 59.9 |
| Ni | 40.1 |

**Fig. S43** (a) TEM image and particle size distribution of PdNi/HCN. (b) XRD pattern of PdNi/HCN, which shows the single-phase and no impurity, has no significant diffraction peak is detected. (c) STEM-EDS mappings, (d) EDS spectrum and (e) atomic ratio of the selected PdNi particles. The reason why the absence of distinct alloy diffraction peaks maybe from the low content or ultrasmall size of the crystal structure, which agrees with a previous study on an ultrasmall high entropy alloy nanocrystal [16].

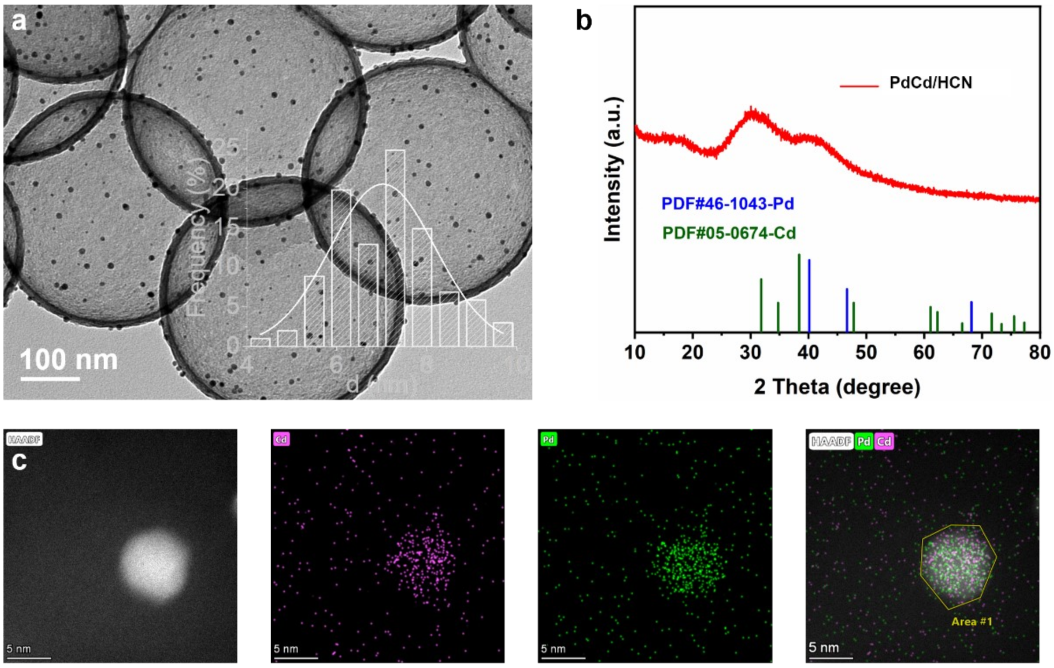


d

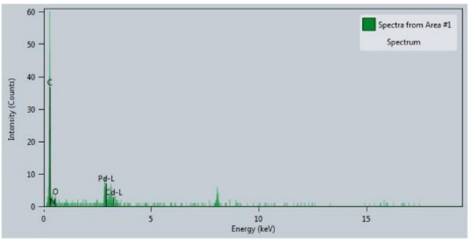


e

| Elemental | Atom% |
|---|---|
| Pd | 72.2 |
| Cd | 27.8 |

**Fig. S44** (a) TEM image and particle size distribution of PdCd/HCN. (b) XRD pattern of PdCd/HCN, which shows the single-phase and no impurity, has no significant diffraction peak is detected. (c) STEM-EDS mappings, (d) EDS spectrum and (e) atomic ratio of the selected PdCd particles. The reason why the absence of distinct alloy diffraction peaks maybe from the low content or ultrasmall size of the crystal structure, which agrees with a previous study on an ultrasmall high entropy alloy nanocrystal.

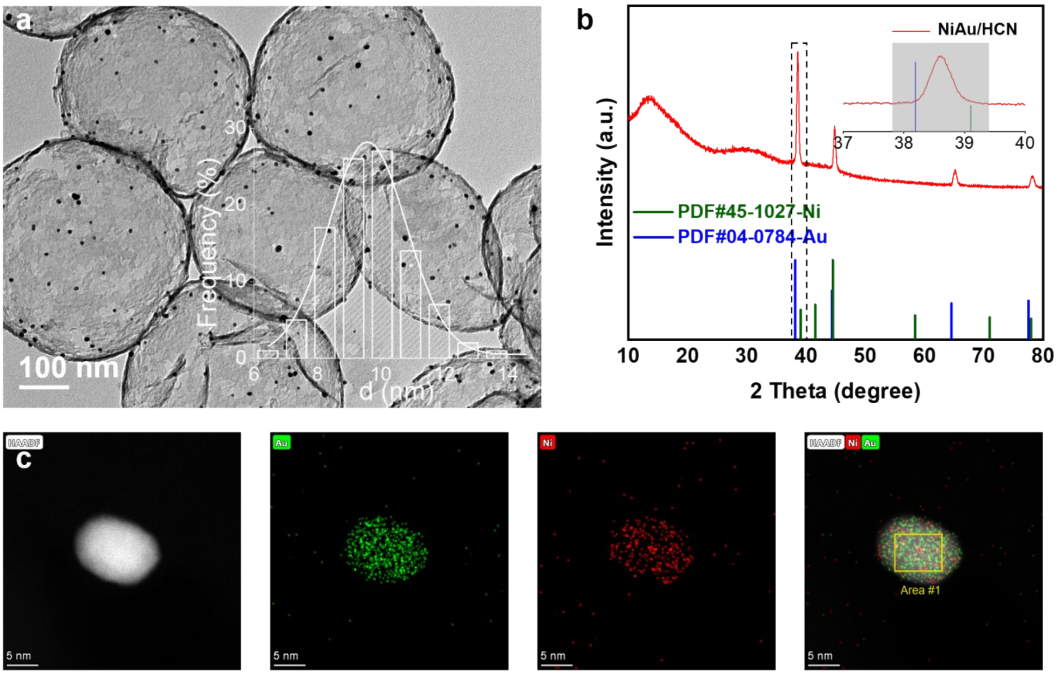


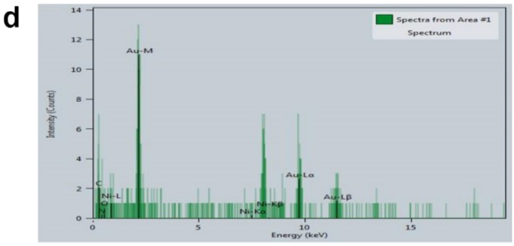


e

| Elemental | Atom% |
|---|---|
| Au | 83.9 |
| Ni | 16.1 |

**Fig. S45** (a) TEM image and particle size distribution of AuNi/HCN. (b) XRD pattern of AuNi/HCN. The main diffraction peak position is lied between Ni and Au standards, which reveals the formation of AuNi nanoalloy. (c) STEM-EDS mappings, (d) EDS spectrum and (e) atomic ratio of the selected AuNi particle.

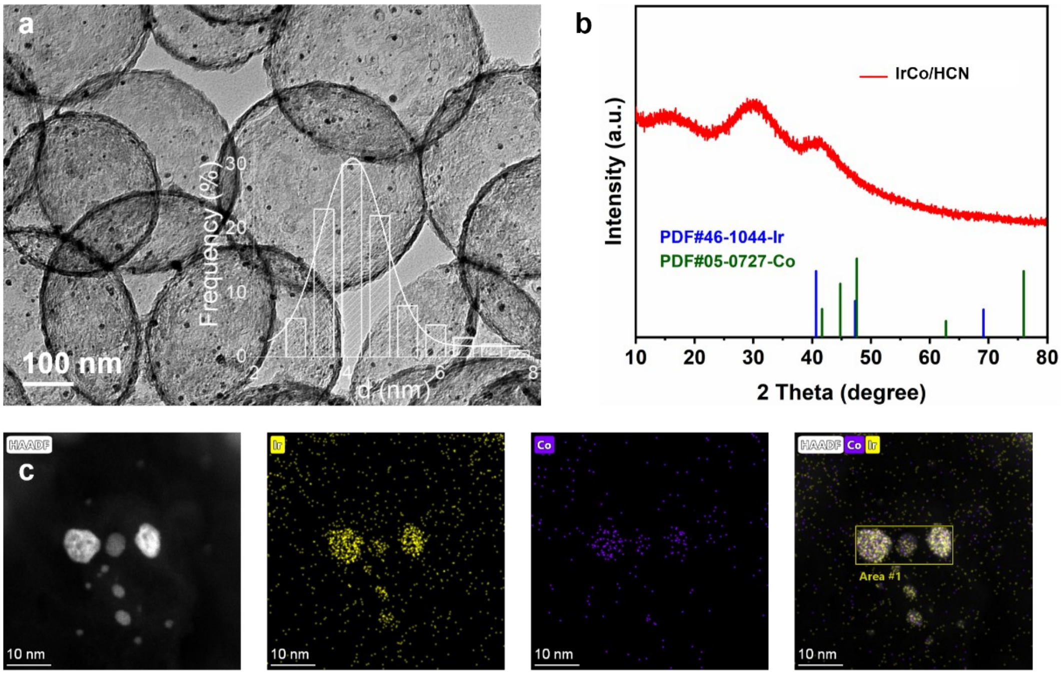


d

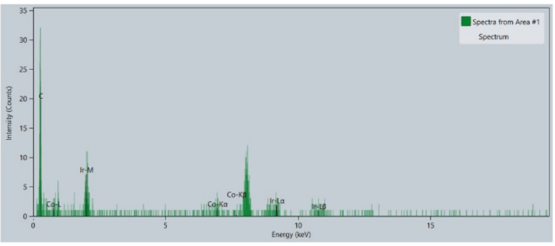


e

| Elemental | Atom% |
|---|---|
| Ir | 55.1 |
| Co | 44.9 |

**Fig. S46** (a) TEM image and particle size distribution of IrCo/HCN. (b) XRD pattern of IrCo/HCN, which shows the single-phase and no impurity, has no significant diffraction peak is detected. (c) STEM-EDS mappings, (d) EDS spectrum and (e) atomic ratio of the selected IrCo particles. The reason why the absence of distinct alloy diffraction peaks maybe from the low content or ultrasmall size of the crystal structure, which agrees with a previous study on an ultrasmall high entropy alloy nanocrystal.

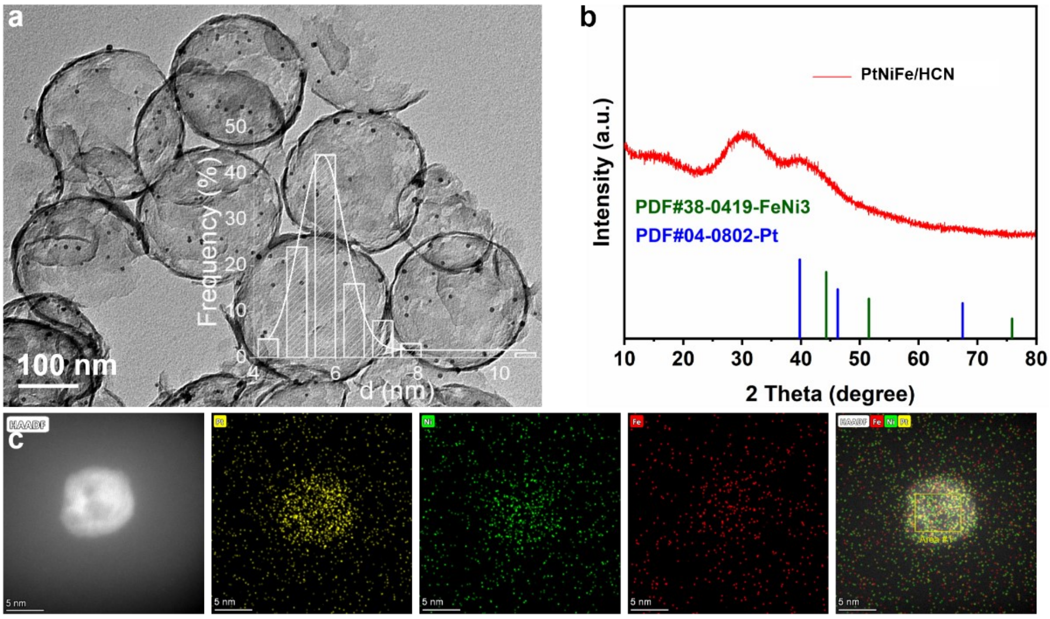


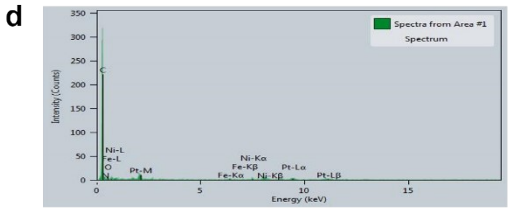


e

| Elemental | Atom% |
|---|---|
| Pt | 47.0 |
| Ni | 28.8 |
| Fe | 18.2 |

**Fig. S47** (a) TEM image and particle size distribution of PtNiFe/HCN. (b) XRD pattern of PtNiFe/HCN, which shows the single-phase and no impurity, has no significant diffraction peak is detected. (c) STEM-EDS mappings, (d) EDS spectrum and (e) atomic ratio of the selected PtNiFe particles. The reason why the absence of distinct alloy diffraction peaks maybe from the low content or ultrasmall size of the crystal structure, which agrees with a previous study on an ultrasmall high entropy alloy nanocrystal.

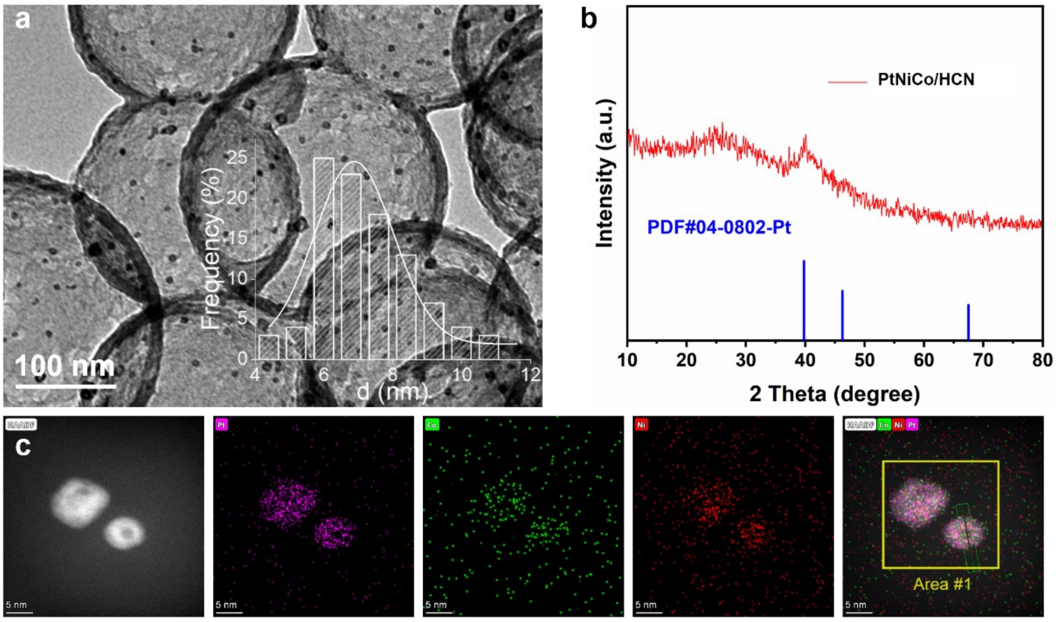


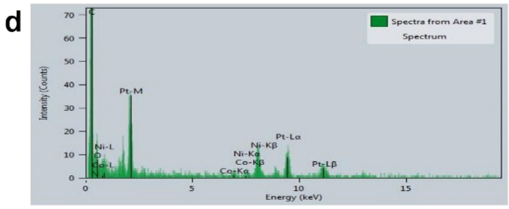


e

| Elemental | Atom% |
|---|---|
| Pt | 78.7 |
| Ni | 8.9 |
| Co | 12.4 |

**Fig. S48** (a) TEM image and particle size distribution of PtNiCo/HCN. (b) XRD pattern of the prepared PtNiCo/HCN. The main diffraction peak position moves to higher angle than the Pt standard, which reveals the formation of PtNiCo nanoalloy. (c) STEM-EDS mappings, (d) EDS spectrum and (e) atomic ratio of the selected PtNiCo particles.

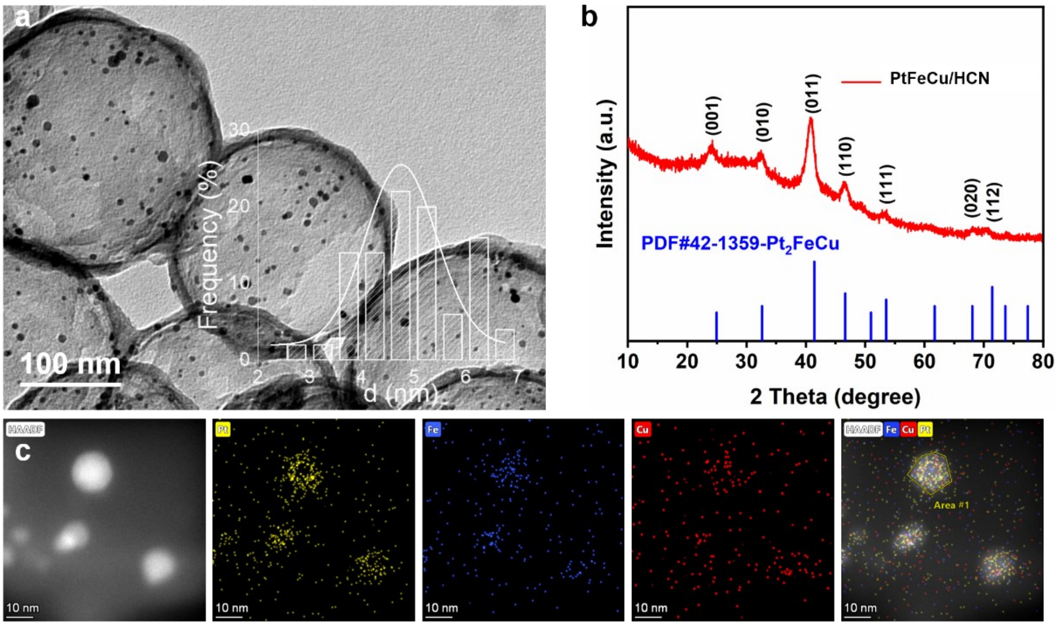


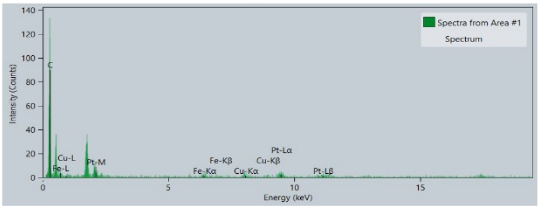


e

| Elemental | Atom% |
|---|---|
| Pt | 42.8 |
| Fe | 36.7 |
| Cu | 20.5 |

**Fig. S49** (a) TEM image and particle size distribution of PtFeCu/HCN. (b) XRD pattern of PtFeCu/HCN. The diffraction peaks of PtFeCu/HCN agree with the ordered $Pt_2FeCu$ standard, confirming the $Pt_2FeCu$ intermetallic formation. (c) STEM-EDS mappings, (d) EDS spectrum and (e) atomic ratio of the selected PtFeCu particles.

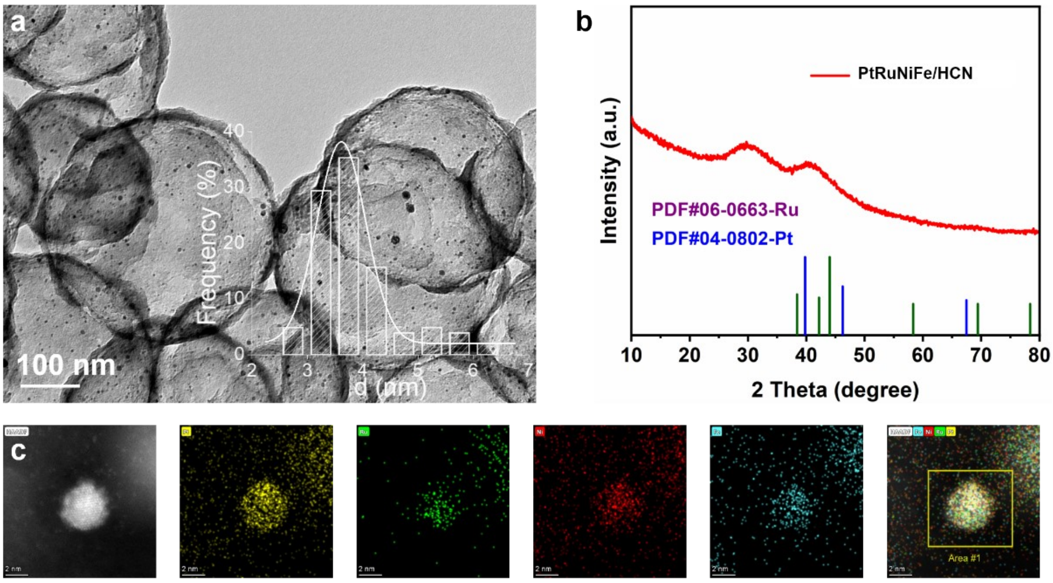


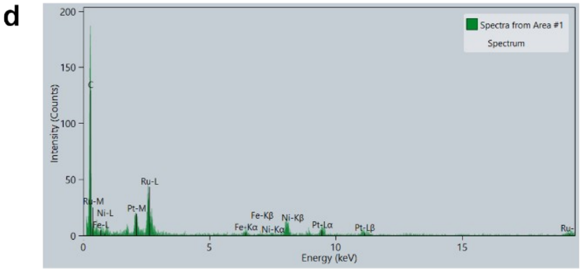


e

| Elemental | Atom% |
|---|---|
| Pt | 24.2 |
| Ru | 53.0 |
| Ni | 9.1 |
| Fe | 13.7 |

**Fig. S50** (a) TEM image and particle size distribution of PtRuNiFe/HCN. (b) XRD pattern of PtRuNiFe/HCN, which shows the single-phase and no impurity, has no significant diffraction peak is detected. (c) STEM-EDS mappings, (d) EDS spectrum and (e) atomic ratio of the selected PtRuNiFe particle. The reason why the absence of distinct alloy diffraction peaks maybe from the low content or ultrasmall size of the crystal structure, which agrees with a previous study on an ultrasmall high entropy alloy nanocrystal.

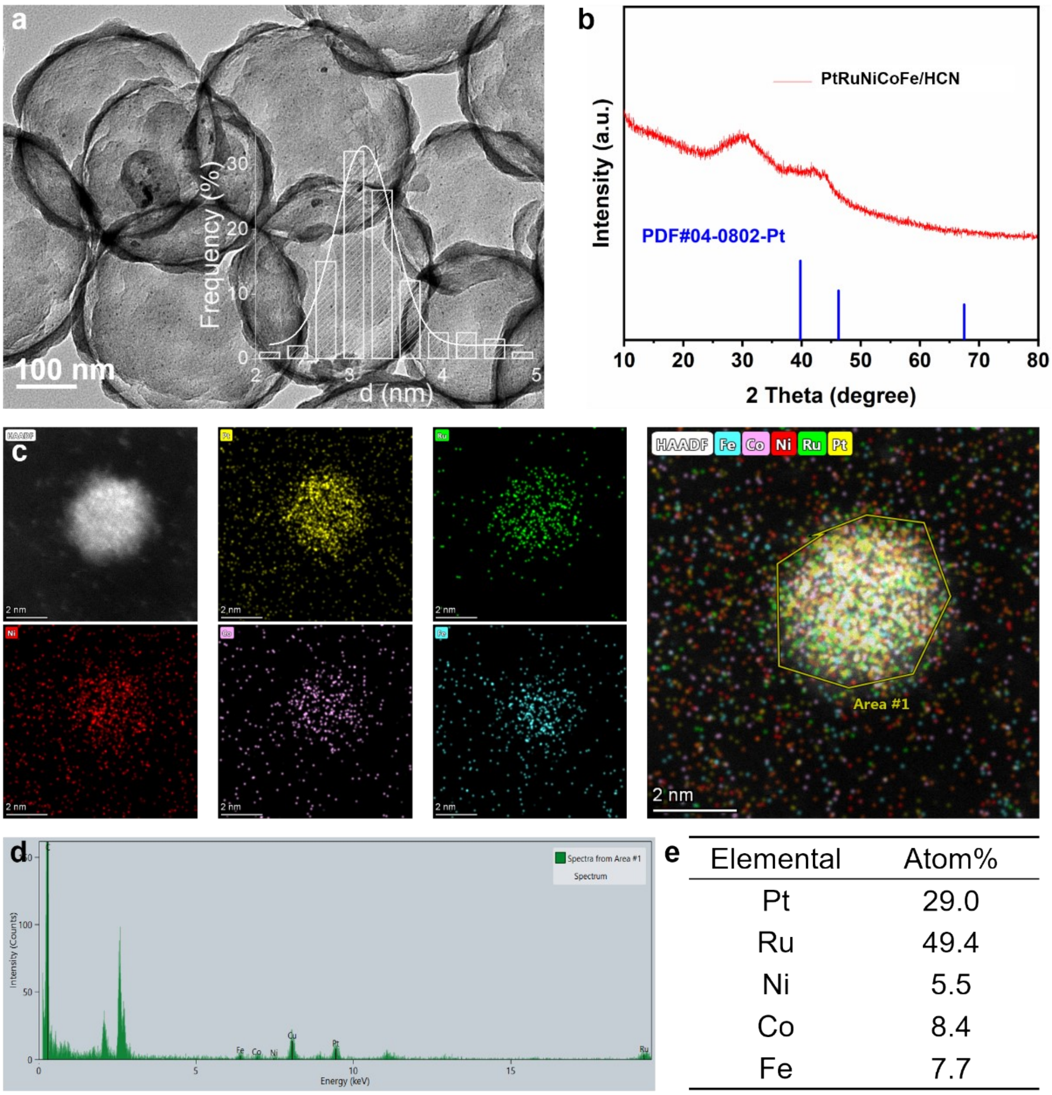


| Elemental | Atom% |
|---|---|
| Pt | 29.0 |
| Ru | 49.4 |
| Ni | 5.5 |
| Co | 8.4 |
| Fe | 7.7 |

**Fig. S51** (a) TEM image and particle size distribution of PtRuNiCoFe/HCN. (b) XRD pattern of PtRuNiCoFe/HCN, which shows the single-phase and no impurity, has no significant diffraction peak is detected. (c) STEM-EDS mappings, (d) EDS spectrum and (e) atomic ratio of the selected PtRuNiCoFe particle. The reason why the absence of distinct alloy diffraction peaks maybe from the low content or ultrasmall size of the crystal structure, which agrees with a previous study on an ultrasmall high entropy alloy nanocrystal.

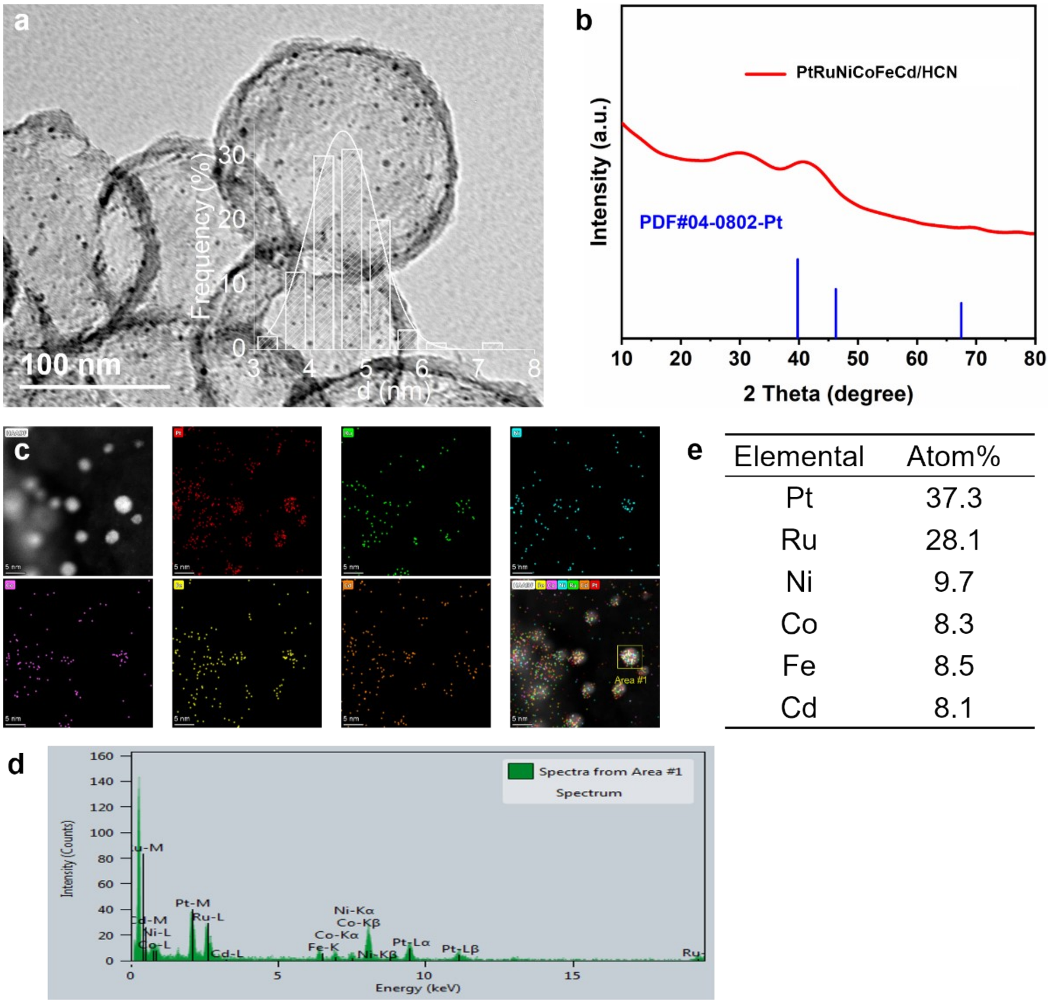


| Elemental | Atom% |
|---|---|
| Pt | 37.3 |
| Ru | 28.1 |
| Ni | 9.7 |
| Co | 8.3 |
| Fe | 8.5 |
| Cd | 8.1 |

**Fig. S52** (a) TEM image and particle size distribution of PtRuNiCoFeCd/HCN. (b) XRD pattern of PtRuNiCoFeCd/HCN, which shows the single-phase and no impurity, has no significant diffraction peak is detected. (c) STEM-EDS mappings, (d) EDS spectrum and (e) atomic ratio of the selected PtRuNiCoFeCd particle. The reason why the absence of distinct alloy diffraction peaks maybe from the low content or ultrasmall size of the crystal structure, which agrees with a previous study on an ultrasmall high entropy alloy nanocrystal.

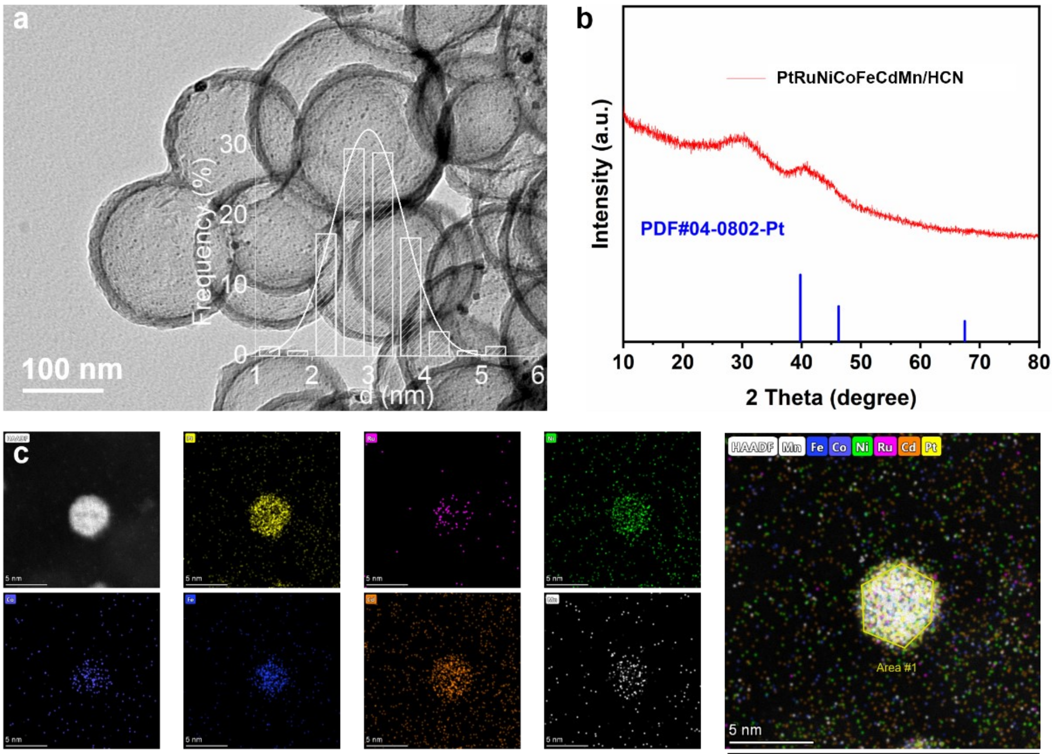


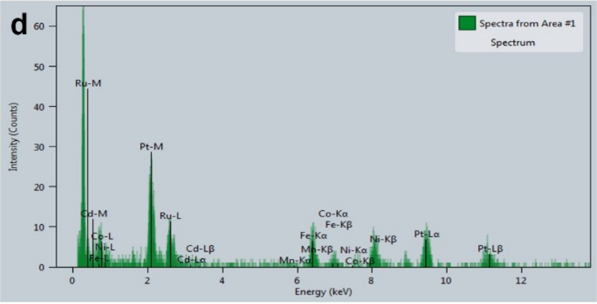


e

| Elemental | Atom% |
|---|---|
| Pt | 34.8 |
| Ru | 27.6 |
| Ni | 9.1 |
| Co | 6.7 |
| Fe | 7.4 |
| Cd | 6.1 |
| Mn | 8.3 |

**Fig. S53** (a) TEM image and particle size distribution of PtRuNiCoFeCdMn/HCN. (b) XRD pattern of PtRuNiCoFeCdMn/HCN, which shows the single-phase and no impurity, has no significant diffraction peak is detected. (c) STEM-EDS mappings, (d) EDS spectrum and (e) atomic ratio of the selected PtRuNiCoFeCdMn particle. The reason why the absence of distinct alloy diffraction peaks maybe from the low content or ultrasmall size of the crystal structure, which agrees with a previous study on an ultrasmall high entropy alloy nanocrystal.

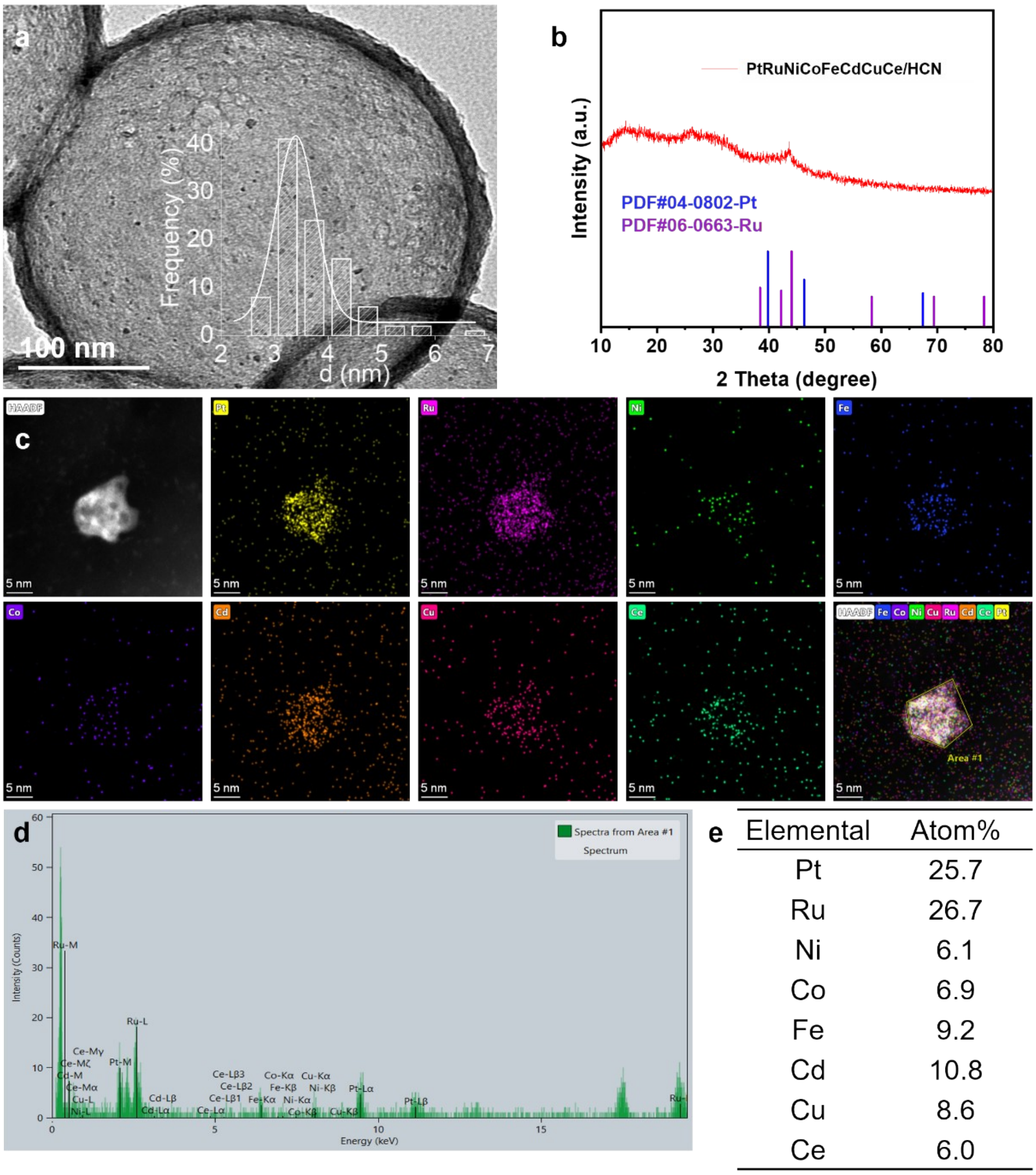


| Elemental | Atom% |
|---|---|
| Pt | 25.7 |
| Ru | 26.7 |
| Ni | 6.1 |
| Co | 6.9 |
| Fe | 9.2 |
| Cd | 10.8 |
| Cu | 8.6 |
| Ce | 6.0 |

**Fig. 54** (a) TEM image and particle size distribution of PtRuNiFeCoCdCuCe/HCN. (b) XRD pattern of the prepared PtRuNiFeCoCdCuCe/HCN, which shows distinct alloy diffraction peaks, confirming the single-phase and no impurity. (c) STEM-EDS mappings, (d) EDS spectrum and (e) atomic ratio of the selected PtRuNiFeCoCdCuCe particles.

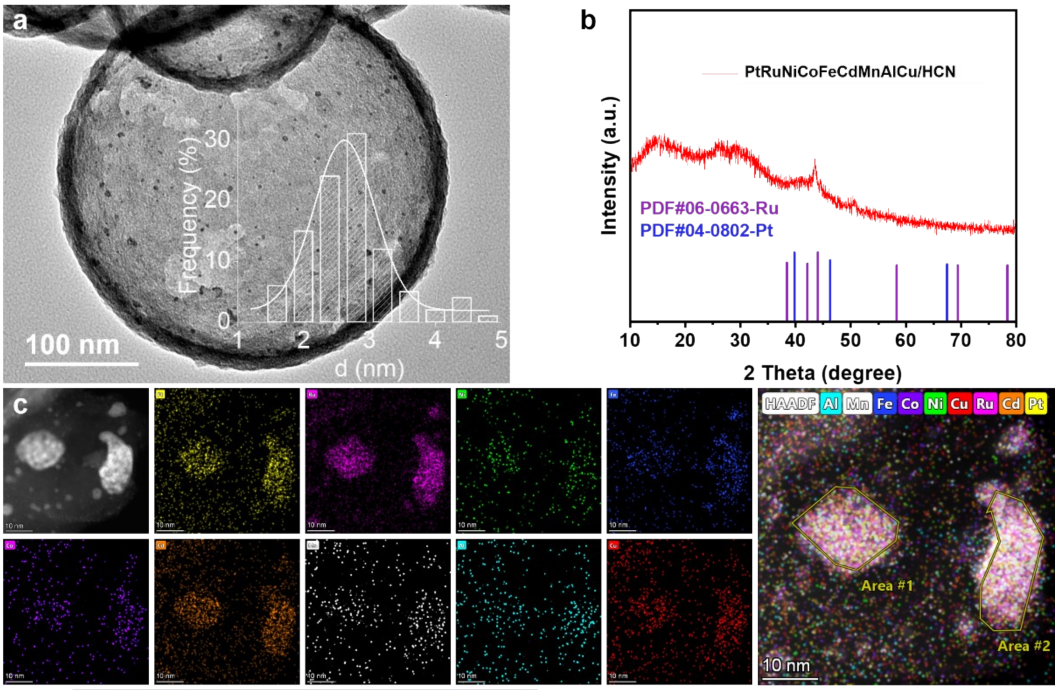


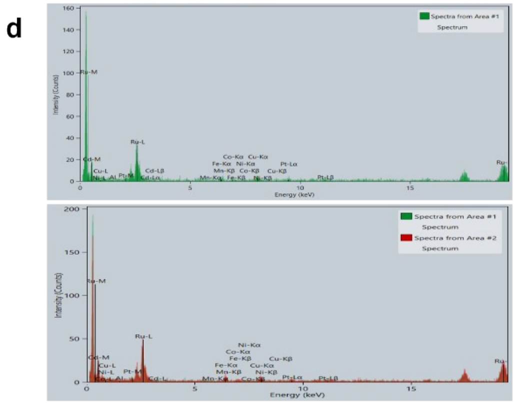


e

| Elemental | Atom% |
|---|---|
| Pt | 19.9 |
| Ru | 23.5 |
| Ni | 5.9 |
| Co | 5.1 |
| Fe | 15.4 |
| Cd | 12.0 |
| Mn | 5.6 |
| Al | 5.8 |
| Cu | 6.8 |

**Fig. S55** (a) TEM image and particle size distribution of PtRuNiFeCoCdMnAlCu/HCN. (b) XRD pattern of the prepared PtRuNiFeCoCdMnAlCu/HCN, which shows distinct alloy diffraction peaks, confirming the single-phase and no impurity. (c) STEM-EDS mappings, (d) EDS spectra and (e) atomic ratio of the selected PtRuNiFeCoCdMnAlCu particles.

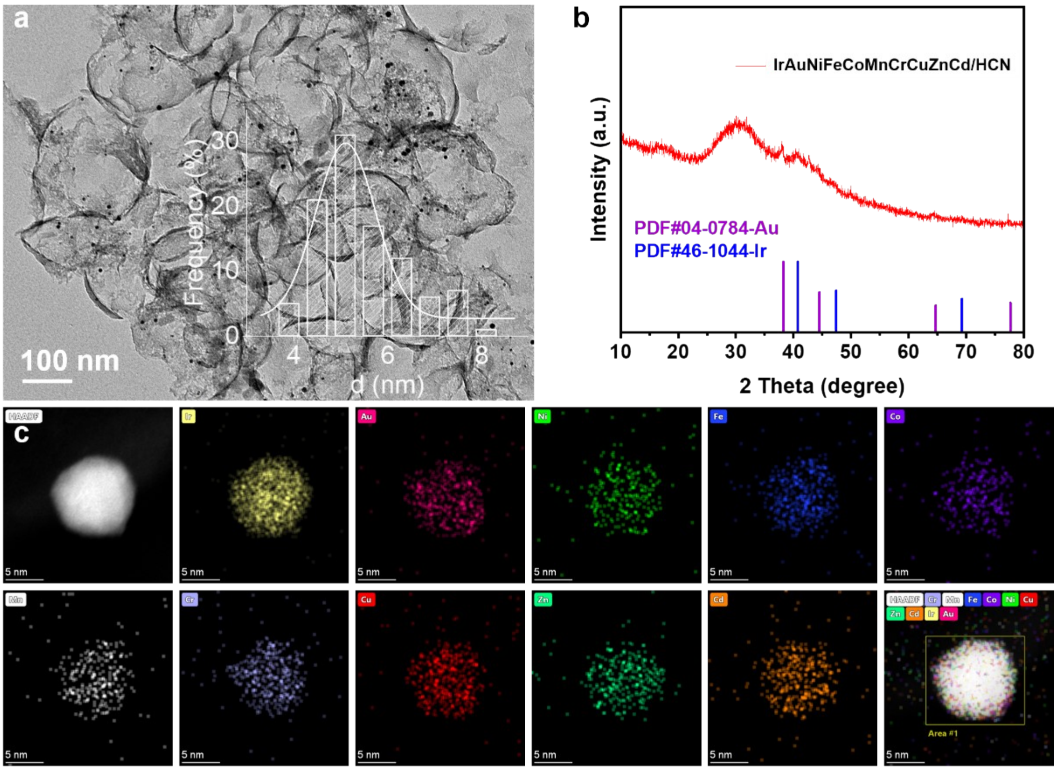


d

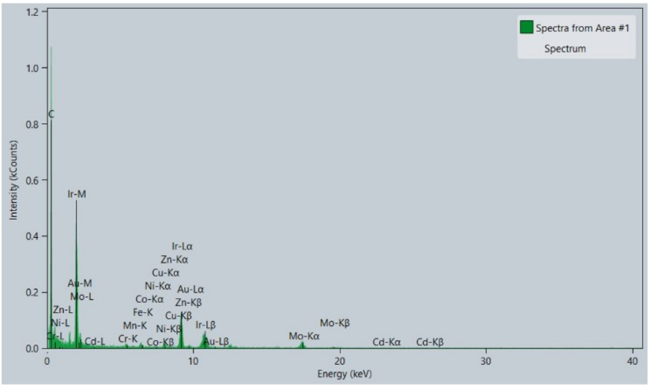


e

| Elemental | Atom% |
|---|---|
| Ir | 27.7 |
| Au | 9.3 |
| Ni | 7.0 |
| Fe | 11.7 |
| Co | 5.3 |
| Mn | 5.2 |
| Cr | 14.1 |
| Cu | 8.1 |
| Zn | 5.7 |
| Cd | 5.9 |

**Fig. S56** (a) TEM image and particle size distribution of IrAuNiFeCoMnCrCuZnCd/HCN. (b) XRD pattern of the prepared IrAuNiFeCoMnCrCuZnCd/HCN confirms the slight shift of weak diffraction peaks position by comparing to Ir and Au XRD standards. (c) STEM-EDS mappings, (d) EDS spectrum and (e) atomic ratio of the selected IrAuNiFeCoMnCrCuZnCd particles.

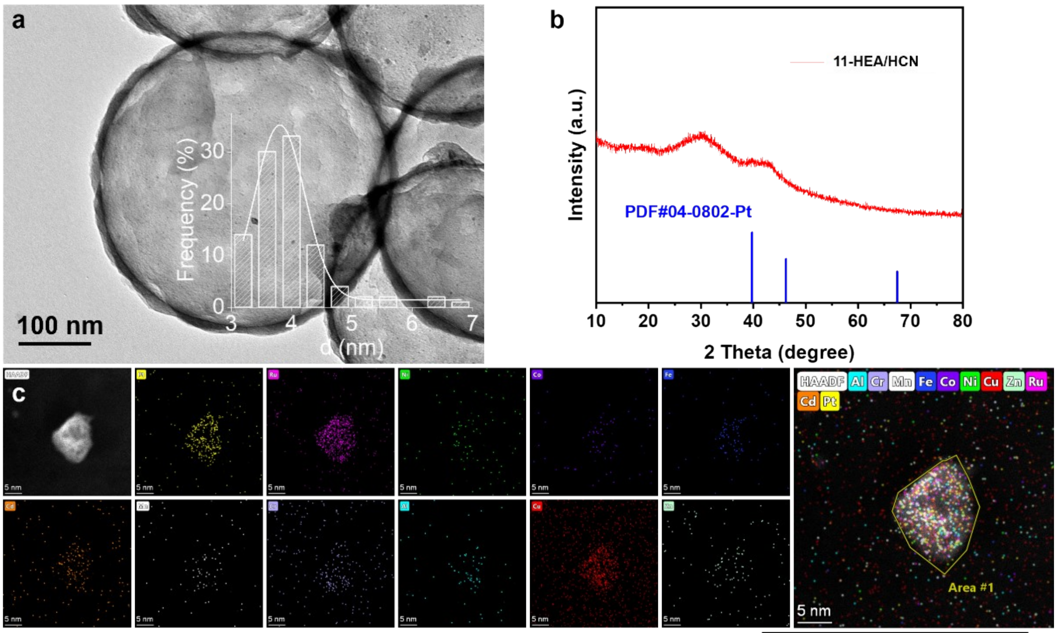


d

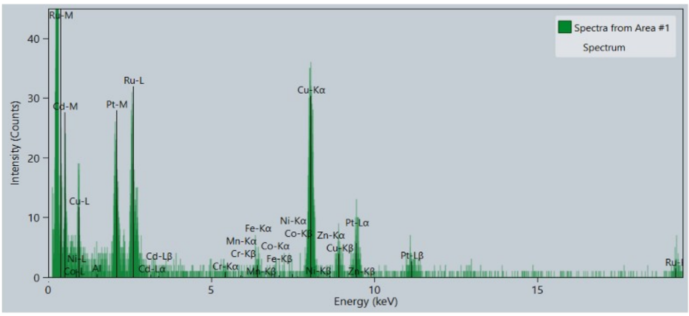


e

| Elemental | Atom% |
|---|---|
| Pt | 17.3 |
| Ru | 14.0 |
| Ni | 5.3 |
| Co | 6.7 |
| Fe | 8.7 |
| Cd | 8.0 |
| Mn | 5.5 |
| Cr | 7.8 |
| Al | 7.3 |
| Cu | 11.7 |
| Zn | 7.7 |

**Fig. S57** (a) TEM image and particle size distribution of 11-HEA (PtRuNiCoFeCdMnCrAlCuZn)/HCN. (b) XRD pattern of 11-HEA/HCN, which shows the single-phase and no impurity, has no significant diffraction peak is detected. (c) STEM-EDS mappings, (d) EDS spectrum and (e) atomic ratio of the selected 11-HEA particle. The reason why the absence of distinct alloy diffraction peaks maybe from the low content or ultrasmall size of the crystal structure, which agrees with a previous study on an ultrasmall high entropy alloy nanocrystal.

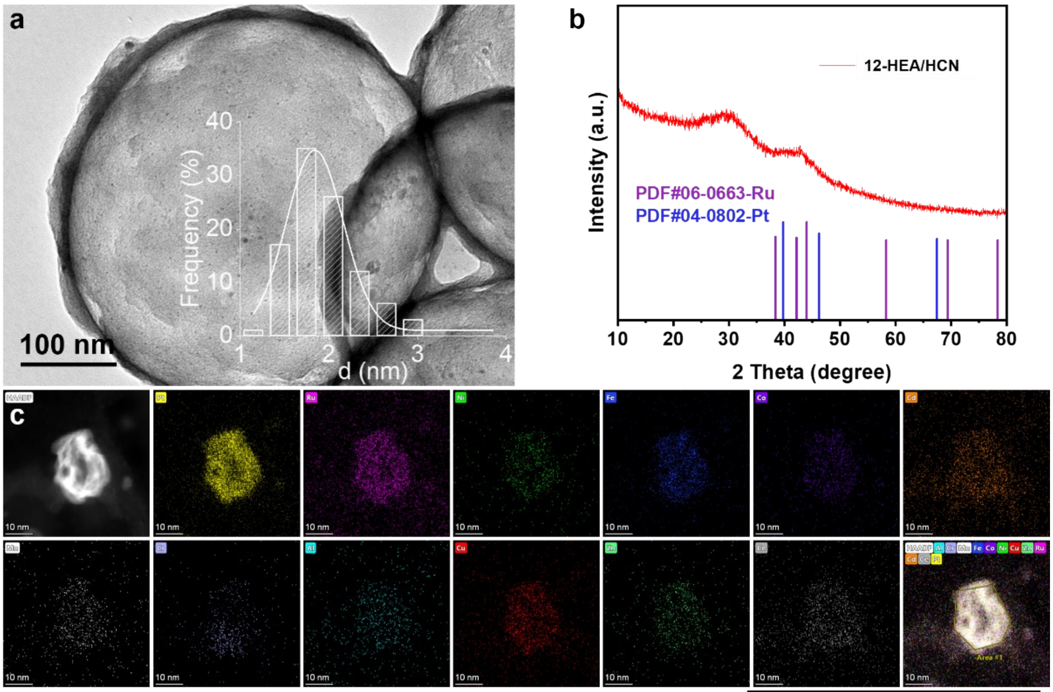


d

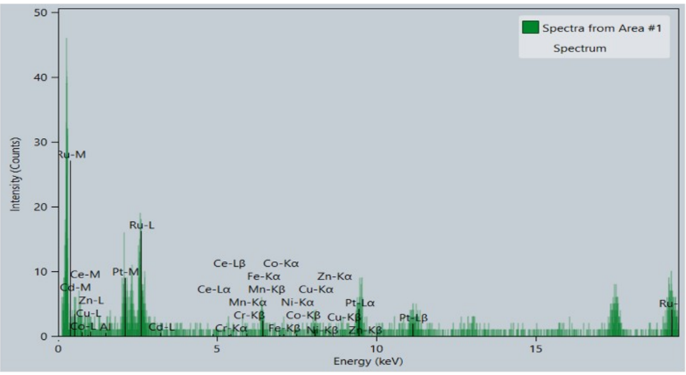


e

| Elemental | Atom% |
|---|---|
| Pt | 14.0 |
| Ru | 9.6 |
| Ni | 5.7 |
| Co | 9.6 |
| Fe | 12.7 |
| Cd | 5.9 |
| Mn | 5.3 |
| Cr | 10.0 |
| Al | 5.9 |
| Cu | 9.2 |
| Zn | 6.2 |
| Ce | 5.9 |

**Fig. S58** (a) TEM image and particle size distribution of 12-HEA (PtRuNiFeCoCdMnCrAlCuZnCe)/HCN. (b) XRD pattern of 12-HEA/HCN, which shows the single-phase and no impurity, has no significant diffraction peak is detected. (c) STEM-EDS mappings, (d) EDS spectrum and (e) atomic ratio of the selected 12-HEA particle. The reason why the absence of distinct alloy diffraction peaks maybe from the low content or ultrasmall size of the crystal structure, which agrees with a previous study on an ultrasmall high entropy alloy nanocrystal.

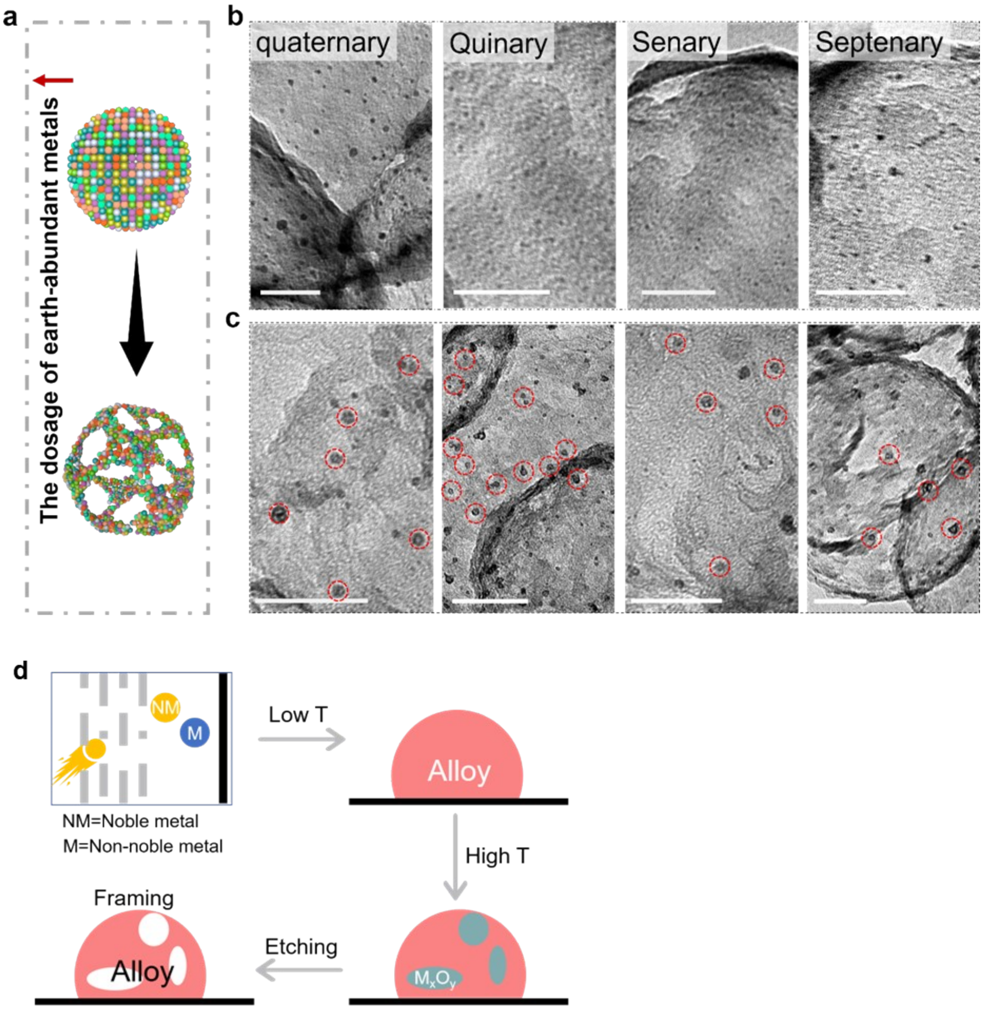


**Fig. S59** (a) Schematic illustration of the tunable structure from (b) solid to (c) frame-work for prepared multielement nanoalloys. Scale bar, 50 nm. (d) An illustration of the mechanism to form a frame structural nanoalloys.

As shown in Fig. 59d, for the formation of the framework structure, we speculate that when the precursor material is annealed at a lower temperature, noble metal and non-precious metal in the sample will migrate and encounter each other in the constructed narrow space, prompting the alloying of two types of metals. Since most non-precious metals are less susceptible to reduction and highly susceptible to oxidation compared to precious metals. Therefore, when an excess of non-precious metal is present, with the increase of annealing temperature, both the non-precious metal oxide phase and the precious metal-based alloy phase may be present in the annealed nanoalloy particles [32]. In the formed nanoparticles with the coexistence of both phases, noble metal and non-precious metal atoms are continuously deposited in the highly reactive regions of the particles, such as edges and corners, by Ostwald ripening and replacement reactions [17]. Finally, the non-precious metal oxide phase within the NAs is selectively etched under acidic conditions, forming a well-defined anisotropic mesoporous framework structure [18,19]. In addition, we have demonstrated that morphological control of framing NAs with varying entropies can be achieved by increasing the addition of non-precious metals. (Fig. S59). The CESC method provides a general and controllable strategy for the structure of nanoalloy frameworks with different entropies.

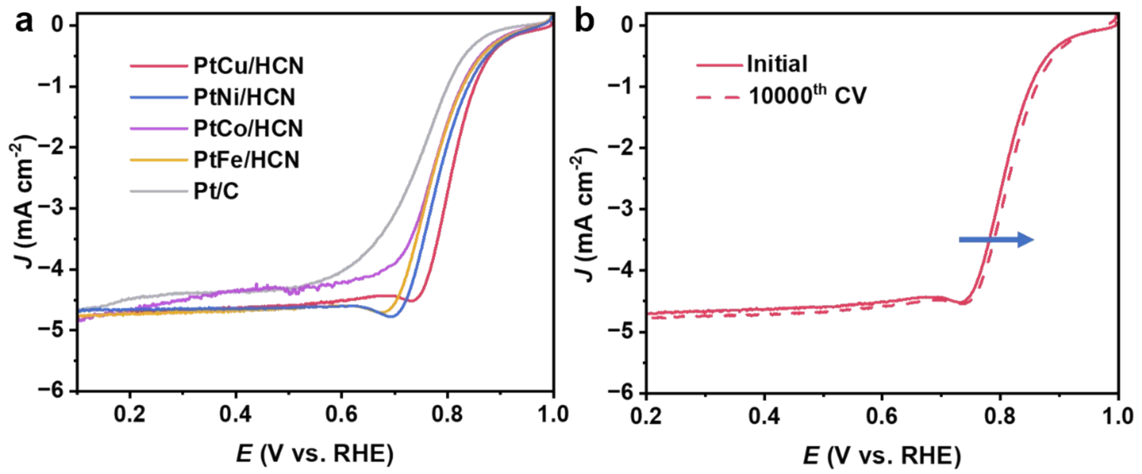


**Fig. S60** (a) ORR polarization curves of the PtM/HCN (M=Cu, Ni, Co, Fe) catalysts in 0.5 M $H_2SO_4$. Pt/C was also tested for comparison. (b) ORR polarization curves recorded before and after 10000$^{th}$ CV cycles for PtCu/HCN.

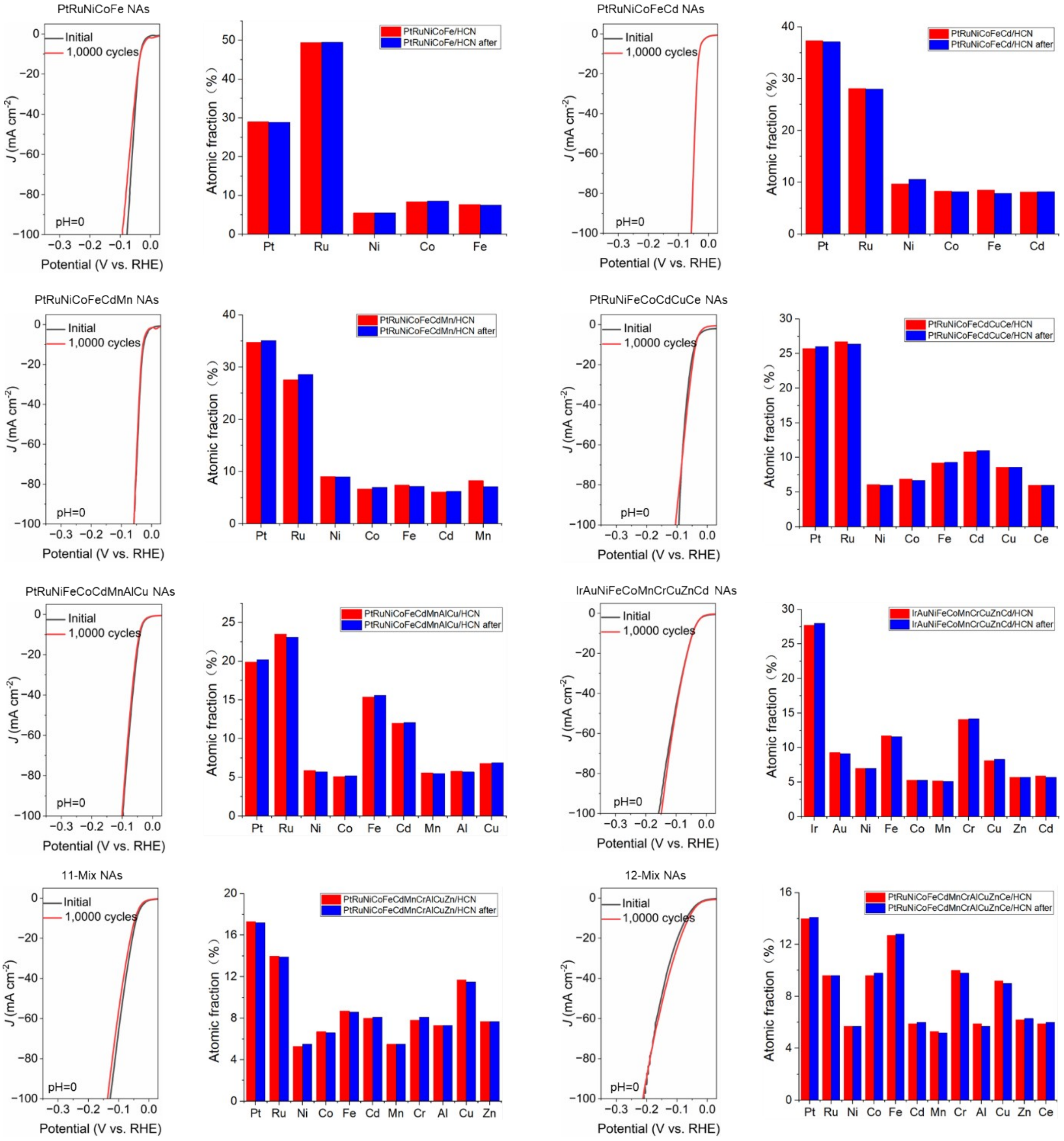


**Fig. S61** Activity and stability tests of the prepared high entropy alloy through potential cycling 10,000 cycles, normalized by geometric area. And the corresponding change of atomic fraction in the composition of them before and after catalytic test was shown.

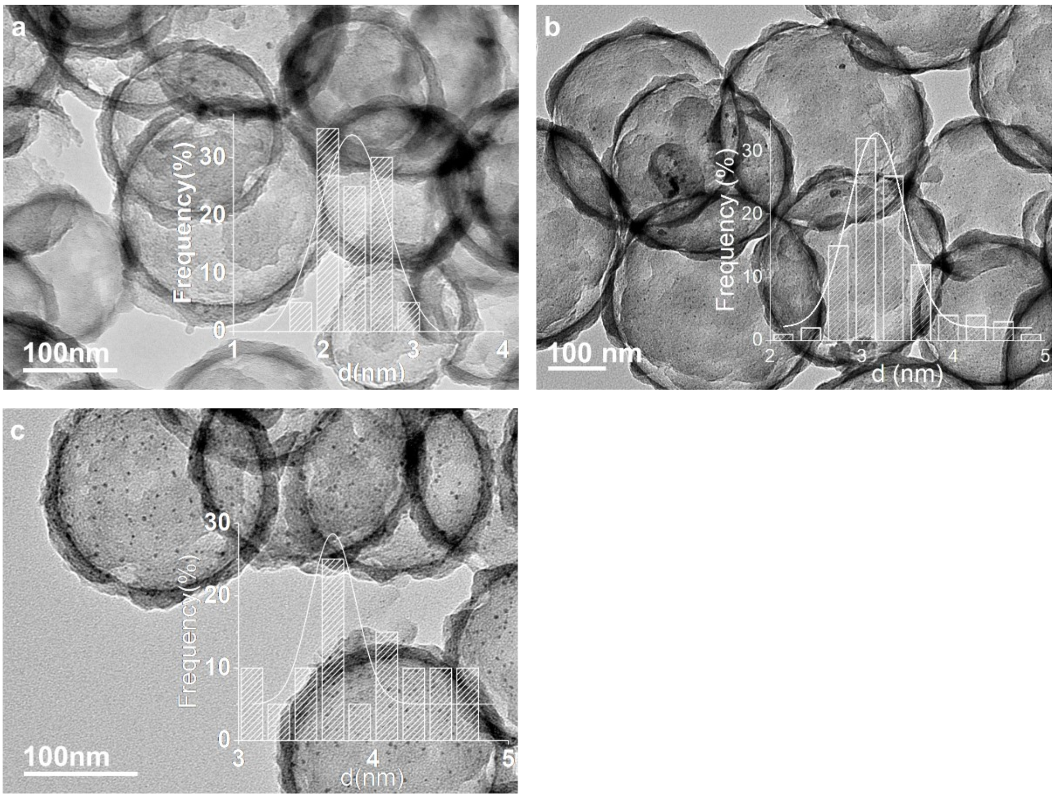


**Fig. S62** TEM images and particle size distribution of PtRuNiCoFe/HCN after sintering temperatures of (a)800℃, (b)900℃, (c)1000℃, respectively.

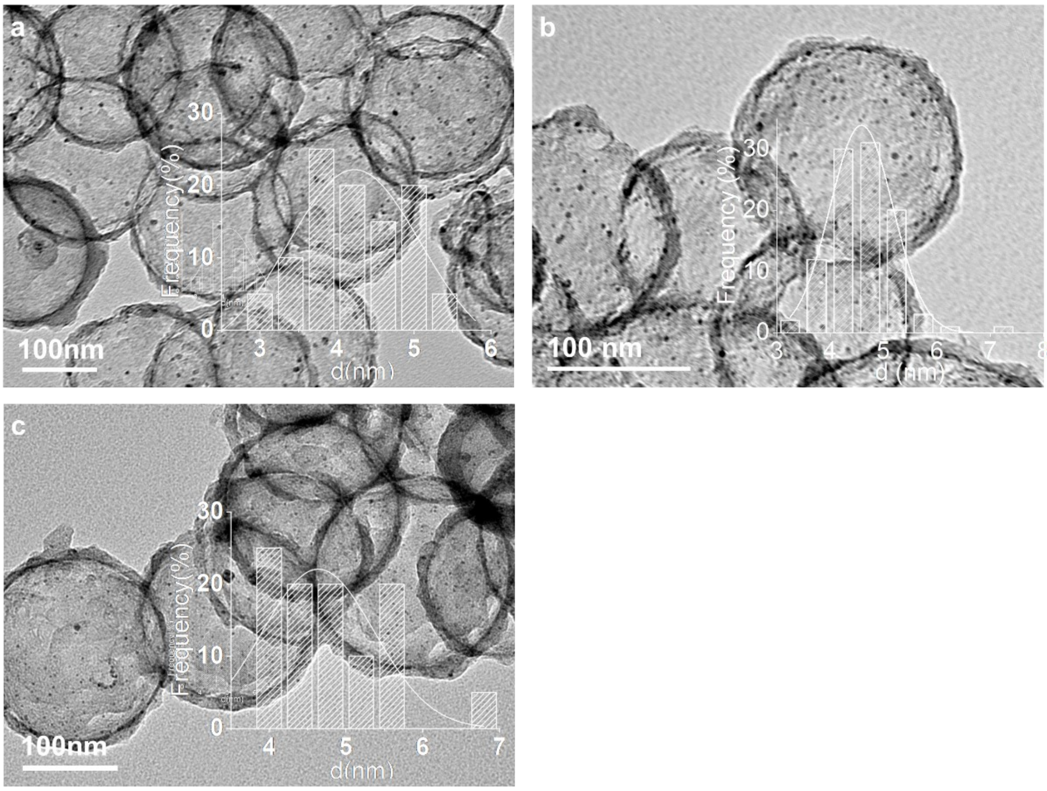


**Fig. S63** TEM images and particle size distribution of PtRuNiCoFeCd/HCN after sintering temperatures of (a)800℃, (b)900℃, (c)1000℃, respectively.

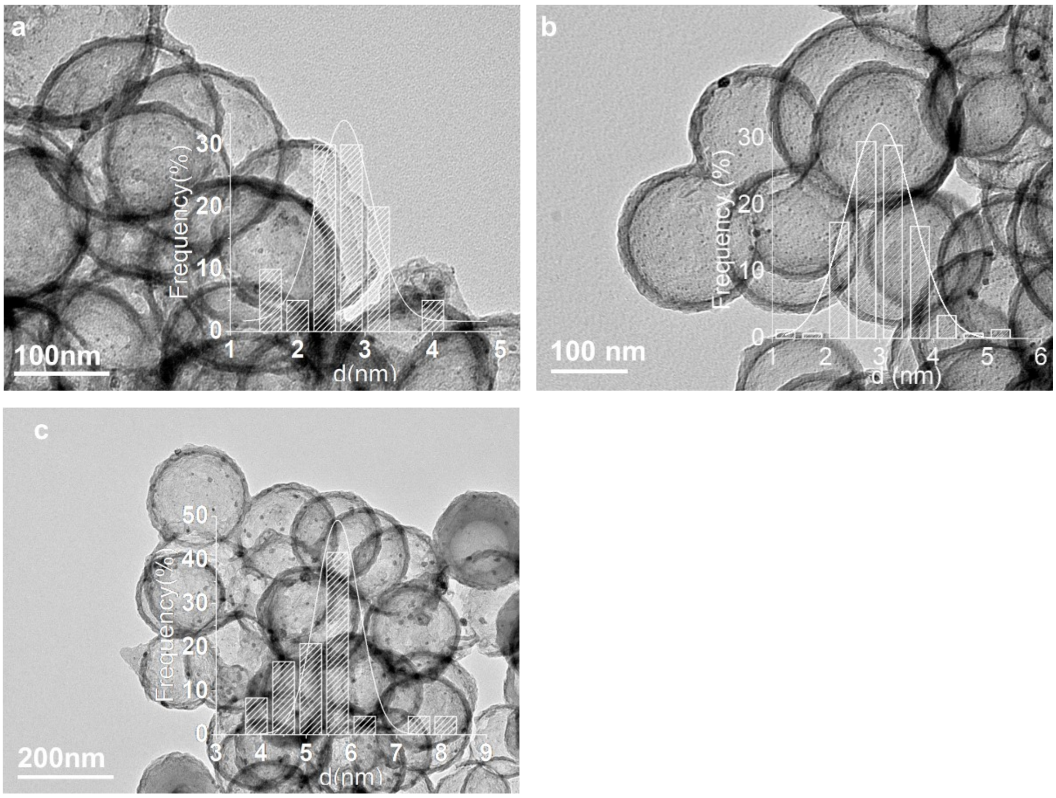


**Fig. S64** TEM images and particle size distribution of PtRuNiCoFeCdMn/HCN after sintering temperatures of (a) 800℃, (b) 900℃, (c) 1000℃, respectively.

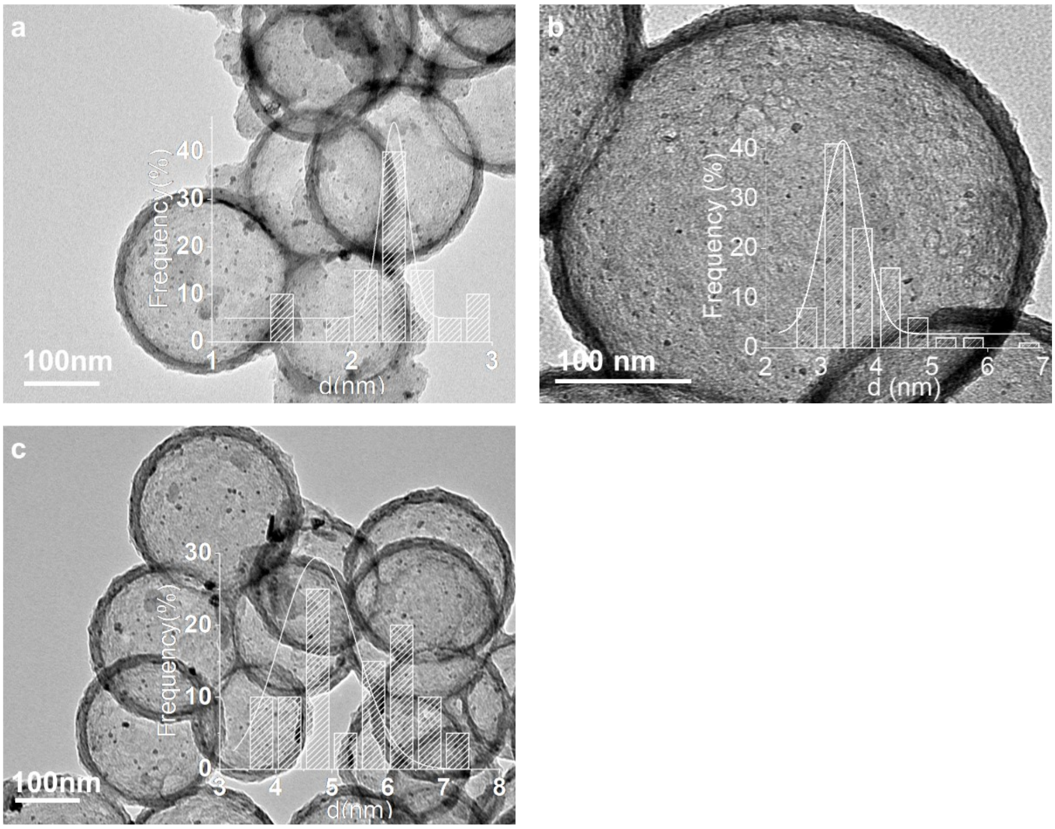


**Fig. S65.** TEM images and particle size distribution of PtRuNiCoFeCdCuCe/HCN after sintering temperatures of (a)800℃, (b)900℃, (c)1000℃, respectively.

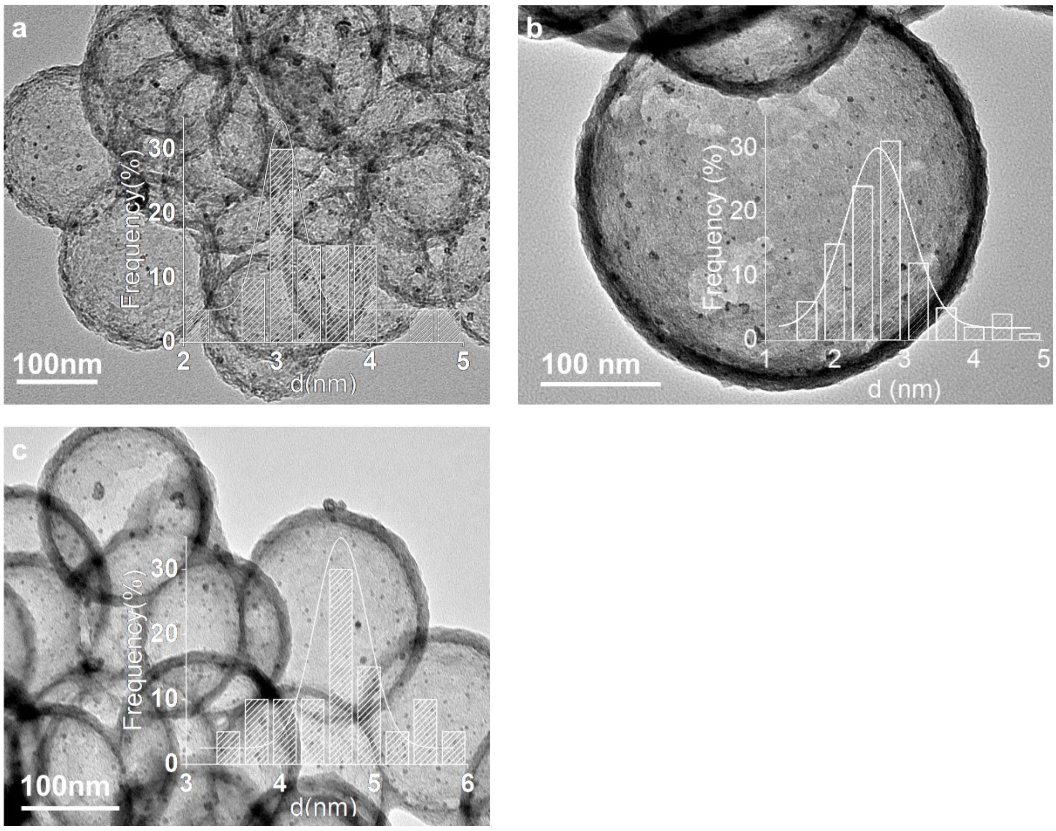


**Fig. S66** TEM images and particle size distribution of PtRuNiCoFeCdMnAlCu/HCN after sintering temperatures of (a)800℃, (b)900℃, (c)1000℃, respectively.

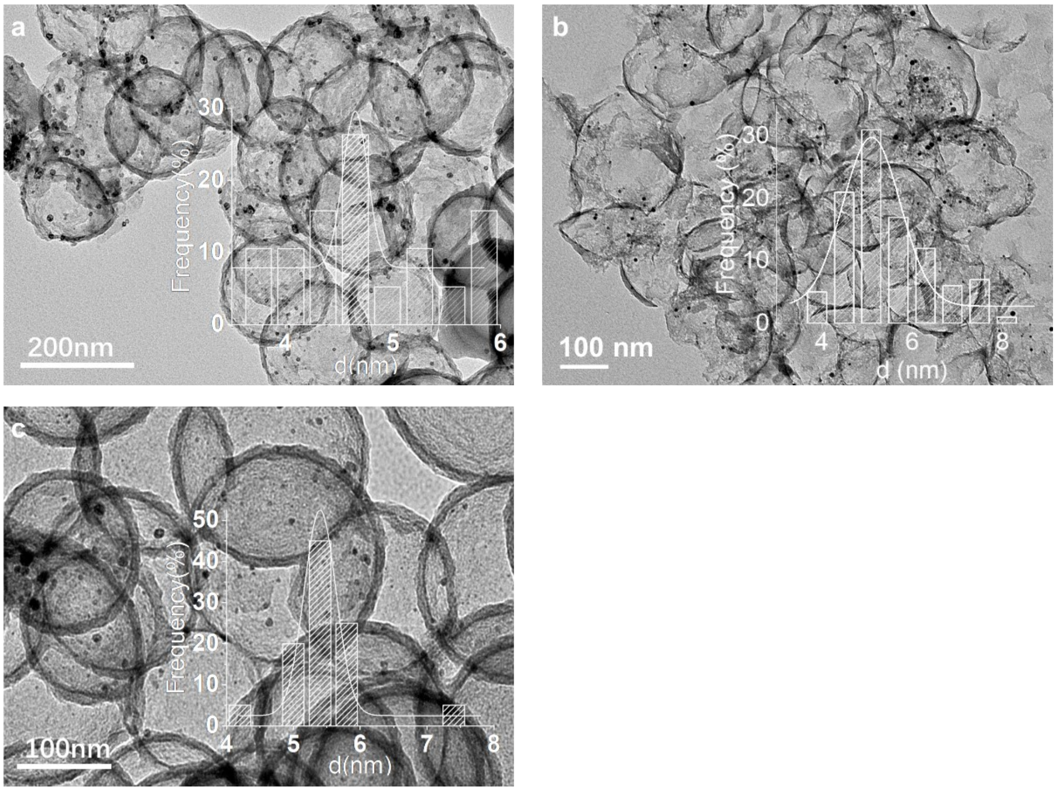


**Fig. S67** TEM images and particle size distribution of IrAuNiFeCoMnCrCuZnCd/HCN after sintering temperatures of (a)800℃, (b)900℃, (c)1000℃, respectively.

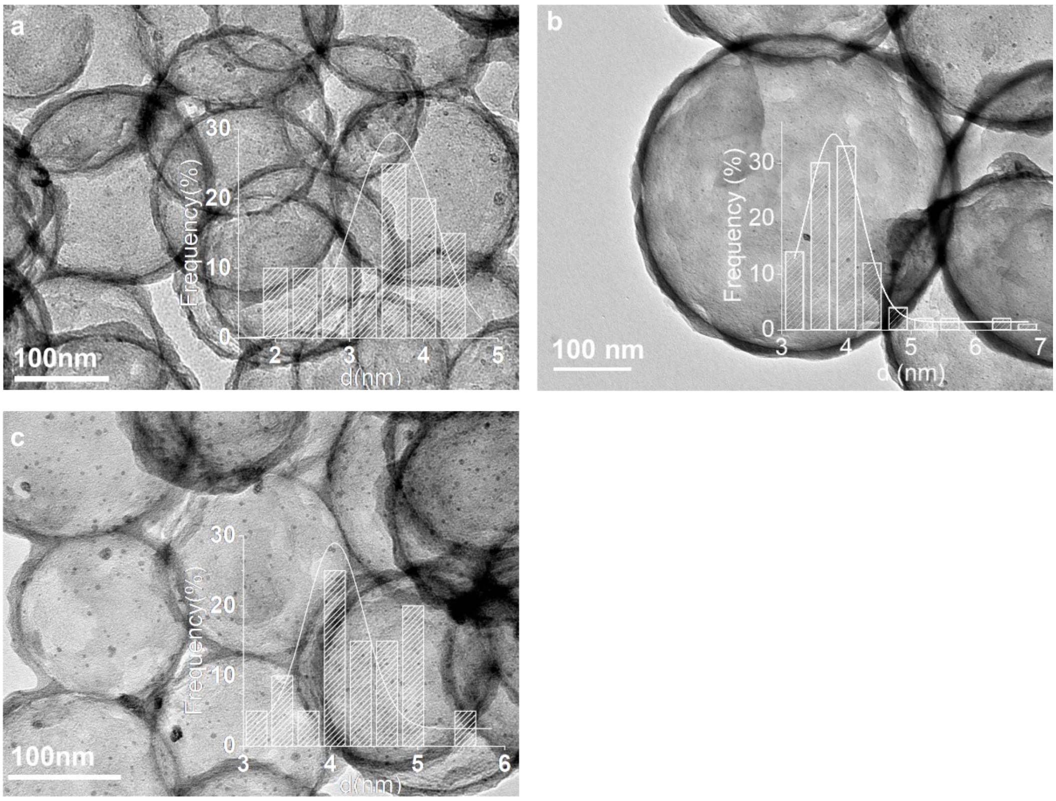


**Fig. S68** TEM images and particle size distribution of PtRuNiCoFeCdMnCrAlCuZn/HCN after sintering temperatures of (a)800°C, (b)900°C, (c)1000°C, respectively.

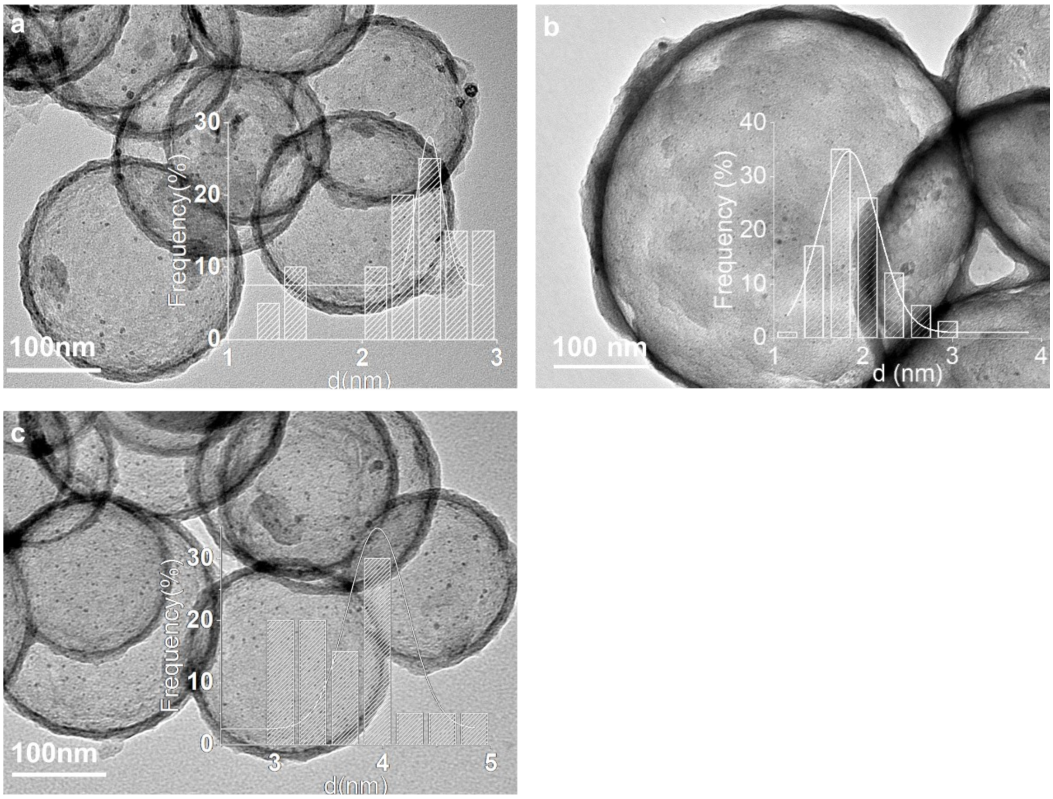


**Fig. S69** TEM images and particle size distribution of PtRuNiCoFeCdMnCrAlCuZnCe/HCN after sintering temperatures of (a)800°C, (b)900°C, (c)1000°C, respectively.

**Table S1.** Curve fit Parameters for Pt $L_3$-edge EXAFS for PtCu/HCN. ($S_0^2$=0.89)

| Sample | Scattering pair | N | *R* (Å) | $\sigma^2$ (Å$^2$) | $\Delta E_0$ (eV) | *R*-factor |
|---|---|---|---|---|---|---|
| | Pt-Cu | 8.97 | 2.63±0.03 | 0.01 | | |
| PtCu/HCN | Pt-Pt | 1.02 | 2.89±0.12 | 0.03 | 7.25 | 0.02 |
| | Pt-O | 0.23 | 1.96±0.1 | 0.002 | | |
| | Cu-Pt | 6.17 | 2.87±0.25 | 0.01 | | |
| PtCu/HCN | Cu-Cu | 2.20 | 2.61±0.01 | 0.008 | 13.35 | 0.006 |
| | Cu-O | 0.88 | 1.96±0.02 | 0.005 | | |
| Pt-foil | Pt-Pt | 12* | 2.77±0.008 | 0.004±0.001 | 8.36 | 0.004 |
| Cu-foil | Cu-Cu | 12* | 2.54±0.003 | 0.008±0.001 | 5.06 | 0.003 |

$S_0^2$ is the amplitude reduction factor; N is the coordination number; R is interatomic distance (the bond length between central atoms and surrounding coordination atoms); $\sigma^2$ is Debye-Waller factor (a measure of thermal and static disorder in absorber-scatterer distances); $\Delta E_0$ is edge-energy shift (the difference between the zero kinetic energy value of the sample and that of the theoretical model). R factor is used to value the goodness of the fitting. *This value was fixed during EXAFS fitting, based on the known structure of Pt metal and Cu metal. Data ranges: $3<k<11$ angstrom, $1.0<R<3.0$ angstrom.

**Table S2.** Comparison of calculated surface areas (ECSAs) for PtM/HCN and Pt/C samples obtained from TPD and $H_{upd}$.

| Sample | TPD ($m^2 \cdot g_{Pt}^{-1}$) | $H_{upd}$ ($m^2 \cdot g_{Pt}^{-1}$) |
|---|---|---|
| PtCu/HCN | 90.79 | 39.99 |
| PtNi/HCN | 68.01 | 39.41 |
| PtFe/HCN | 49.04 | 26.48 |
| PtCo/HCN | 37.61 | 35.67 |
| Pt/C | 70.00 | 83.95 |

**Table S3.** ICP-MS results for all PtM/HCN samples shown in this table. Metal % Pt and Metal % M represent the fraction of Pt and M (Cu, Ni, Fe, Co) in total PtM/HCN sample, while the ratio of metal atoms represents the weight percentage ratio of Pt to M.

| Sample | Metal % (Pt) | Metal % (M) | The ratio of metal atoms |
|---|---|---|---|
| PtCu/HCN | 9.59 | 4.37 | Pt: Cu=0.42: 0.58 |
| PtNi/HCN | 7.77 | 5.45 | Pt: Ni=0.30: 0.70 |
| PtFe/HCN | 8.58 | 4.38 | Pt: Fe=0.36: 0.64 |
| PtCo/HCN | 5.05 | 2.19 | Pt: Co=0.41: 0.59 |

**Table S4.** MOR performance of PtCu/HCN catalyst and state-of-the-art Pt-based nanocatalysts from recent published works.

| Sample name | Solution condition | MA ($A \cdot mg_{Pt}^{-1}$) | SA ($mA \cdot cm^{-2}$) | CA (s) | Ref. |
|---|---|---|---|---|---|
| PtCu/HCN | 0.1 M $HClO_4$ + 1 M $CH_3OH$ | 2.81 | 7.02 | 50000 | This work |
| CuNi/Pt-Cu | 0.1 M $HClO_4$ + 1 M $CH_3OH$ | 0.99 | 7.49 | 1800 | [20] |
| porous Pt-Ag nanotubes | 0.1 M $HClO_4$ + 1 M $CH_3OH$ | 2.08 | 6.63 | N/A | [21] |
| PtRuCu nanocrystalline alloy | 0.1 M $HClO_4$ + 1 M $CH_3OH$ | 1.35 | 5.2 | N/A | [22] |
| PtCoNiRh nanowires | 0.1 M $HClO_4$ + 0.5 M $CH_3OH$ | 1.36 | 2.08 | 10000 | [23] |
| $Pt_3CoRu/C/NC$ $Pt_3CoRu$ | 0.1 M $HClO_4$ + 0.5 M $CH_3OH$ | 0.97 | 1.60 | 6000 | [24] |
| PtRu/PC–H | 0.1 M $HClO_4$ + 0.5 M $CH_3OH$ | 1.674 | 4.41 | 7200 | [25] |
| PtPb concave nanocubes | 0.1 M $HClO_4$ + 0.5 M $CH_3OH$ | 0.97 | 2.09 | 6000 | [26] |
| Pt/SiC-3 | 0.5 M $H_2SO_4$ + 0.5 M $CH_3OH$ | 0.647 | N/A | 6000 | [27] |
| AL-Pt/$Pt_3Ga$ | 0.5 M $H_2SO_4$ + 1 M $CH_3OH$ | 1.094 | 7.195 | 1000 | [28] |
| PtNi CNCs | 0.5 M $H_2SO_4$ + 0.5 M $CH_3OH$ | 0.696 | 1.37 | 3600 | [29] |
| Pt/$CeO_2$-Pt | 0.5 M $H_2SO_4$ + 1 M $CH_3OH$ | 0.714 | 8.06 | 1000 | [30] |
| Pt-WP-CL/AEG-3 | 0.5 M $H_2SO_4$ + 1 M $CH_3OH$ | 2.217 | N/A | 3600 | [31] |
| PtCu nanotubes | 0.5 M $H_2SO_4$ + 1 M $CH_3OH$ | 2.252 | 6.09 | 12000 | [32] |
| PtNiP/P-graphene | 0.5 M $H_2SO_4$ + 1 M $CH_3OH$ | 0.826 | 0.653 | 7200 | [33] |
| PtCo CNCs | 0.5 M $H_2SO_4$ + 1 M $CH_3OH$ | 0.692 | 3.04 | 8000 | [34] |
| $Pt_{72}Ru_{28}$ nanoalloys | 0.1 M $HClO_4$ + 0.5 M $CH_3OH$ | 1.70 | 10.98 | 3600 | [35] |
| PtPdAg HNDs | 0.1 M $HClO_4$ + 0.2 M $CH_3OH$ | 1.58 | 3.75 | 10000 | [36] |
| PtRu NWs | 0.1 M $HClO_4$ + 0.5 M $CH_3OH$ | 0.82 | 1.16 | 4000 | [37] |
| PtNiRh NWs | 0.1 M $HClO_4$ + 0.5 M $CH_3OH$ | 1.72 | 2.49 | 5000 | [38] |
| PtCoNiMo NS | 0.5 M $H_2SO_4$ + 1 M $CH_3OH$ | 1.82 | N/A | N/A | [39] |

**Table S5.** Comparing the catalytic performance of PtCu/HCN with the ever-reported acidic HER catalysts.

| Sample name | Overpotential (mV) | Tafel ($mV \cdot dec^{-1}$) | TOF ($s^{-1}$) | MA ($A\ mg^{-1}$) | Ref. |
|---|---|---|---|---|---|
| PtCu/HCN | 37.3@$\eta_{200}$ | 10.1 | 20.00@-0.1V<br>11.27@-0.05V | 19.54@-0.1V<br>10.55@-0.05V | This work |
| $Pt_1$/hNCNC | 20@$\eta_{20}$ | 24 | 7.67@-0.02V | 7.60@-0.02V | [40] |
| Pt-Au-SiNW-2 | ~266@$\eta_{200}$ | 24 | N/A | ~2.00@-0.06V | [41] |
| Pt-$Mo_2TiC_2T_x$ | 104@$\eta_{200}$ | 30 | N/A | 8.30@-0.077V | [42] |
| $ALD_{50}$Pt/NGNs | ~67@$\eta_{30}$ | 29 | N/A | 10.10@-0.05V | [43] |
| Pt@NHPCP（0.1 M $HClO_4$） | ~200@$\eta_{40}$ | 27 | N/A | 5.00@-0.057V | [44] |
| $Rh_1$-TiC | 86@$\eta_{100}$ | 25 | 5.97@-0.025V | 10.68@-0.025V | [45] |
| $Pt_1$/OLC | 38@$\eta_{10}$ | 35 | 11@-0.05V | 7.40@-0.038V | [46] |
| Ru/OMSNNC | ~145@$\eta_{60}$ | N/A | N/A | 10@-0.1V | [47] |
| Pt-$MoS_2$ | ~100@$\eta_{40}$ | 30 | N/A | N/A | [48] |
| 400-SWNT/Pt | 210@$\eta_{180}$ | 38 | N/A | N/A | [49] |
| Pt@PCM | 105@$\eta_{10}$ | 65.3 | ~3@-0.1V | ~0.2@-0.1V | [50] |
| PtCo@PtSn | ~40@$\eta_{40}$ | 26 | ~0.01@-0.04V | ~0.016@-0.05V | [51] |
| Ru@p-$Co_3HHTP_2$ | 60@$\eta_{60}$ | 33 | N/A | N/A | [52] |
| Pt-$MoS_2$ | 67.4@$\eta_{10}$ | 76 | N/A | N/A | [53] |
| $Pt_{SA}$-$Ti_3C_2T_x$ | 38@$\eta_{10}$ | 45 | ~6@-0.1V | ~7@-0.05V | [54] |
| Ru S/DAs+Ru NC | ~210@$\eta_{100}$ | 44 | N/A | N/A | [55] |
| PtRu/mCNTs | 60@$\eta_{100}$ | 22.6 | N/A | N/A | [56] |
| NMHEA (IrPdPtRhRu) NPs | 60@$\eta_{10}$ | N/A | 8@-0.1V | N/A | [57] |

All test conditions are in 0.5 M $H_2SO_4$.

**Table S6.** The Bader charge of surface metal atoms.

| Catalysts | Pt (111) | CuPt | $CuO_2$ (111) | $Cu_2O$/Pt |
|---|---|---|---|---|
| Average Bader charge of Pt / e | 0 | -0.14 | - | 0.020 |
| Average Bader charge of Cu / e | - | 0.62 | 0.530 | 0.493 |

**Table S7.** Comparison of recently reported nano-alloys prepared by ion exchange or domain-limited effects and other advanced methods.

| Figure and Method | Characteristics | Reference |
|---|---|---|
| 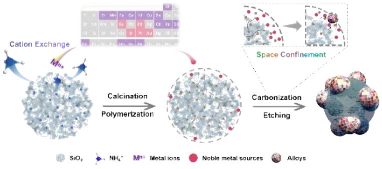<br>Cation exchange &Space confinement | 1. The general preparation of nanoalloys from low to high entropy.<br>2. The controllable synthesis of nanoalloys from nanoparticles to hollow nano-frames.<br>3. The outstanding ability in suppressing alloy sintering for our synthesis method. | This work |
| 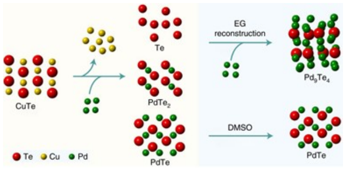<br>Cation exchange | 1. Enables the preparation of binary alloys in 0 to 3 dimensions.<br>2. Expansion of a wide range of noble metal chalcogenides.<br>3. Direct preparation of high entropy alloys is more difficult. | *Nat. Synth* 1, 626–634 (2022) |
| 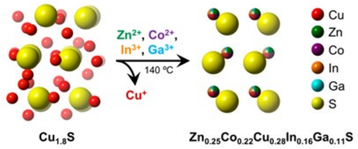<br>Cation exchange | 1. Direct low temperature preparation of high entropy alloys in the liquid phase.<br>2. Limited choice of metal ion species.<br>3. The prepared low entropy alloy has phase separation. | *J. Am. Chem. Soc* 143, 1017-1023 (2021) |
| 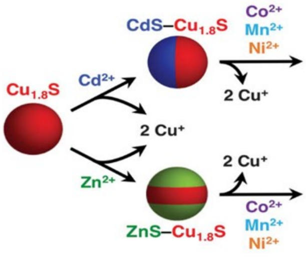<br>Cation exchange | 1. Preparation and expansion of complex heterostructure nanoparticle.<br>2. The simultaneous introduction of multiple metallic elements is more complex.<br>3. Phased alloys rather than solid solution alloys. | *Science* 360, 513–517 (2018) |
| 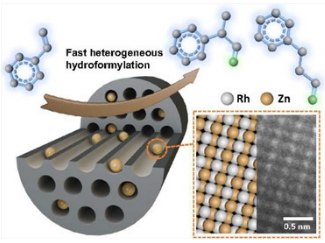 | 1. Small size and atomic ordering of the prepared alloy.<br>2. Lack of ion induction leads to difficulties in introducing multiple metal elements. | *J. Am. Chem. Soc* 143, 20907–20915 (2021) |

| Space confinement | | |
|---|---|---|
| 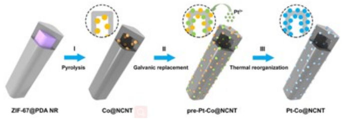<br>Galvanic replacement &Thermal reorganization | 1. Preparation of PtCo alloys with sub-10 nm.<br>2. Limited by the abundance of base metal element species. | Angew. Chem. Int. Ed 60, 1–7 (2021) |
| 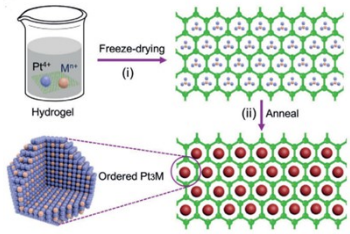<br>Freeze-drying &Confinement effect | 1. Preparation and expansion of ordered Pt-based intermetallic.<br>2. Lack of ion induction leads to difficulties in introducing multiple metal elements. | *Angew. Chem. Int. Ed* 59, 7857 –7863 (2020) |
| 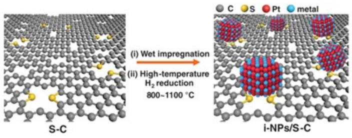<br>Wet impregnation &Sulfur-anchoring | 1. Preparation of 46 low to high entropy Pt-based intermetallic.<br>2. Prepared alloys feature atomic ordering and small size.<br>3. Preparation of alloys requires multiple calcinations. | *Science* 374, 459–464 (2021) |
| 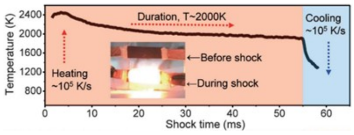<br>Carbothermal shock | 1. Rapid preparation of alloys.<br>2. Prepared alloys feature a wide range of element, small size and different entropies.<br>3. The alloying process is achieved at higher temperatures. | *Science* 359, 1489–1494 (2018) |
| 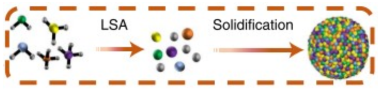<br>Laser beam | 1. Preparation of alloys on different substrates.<br>2. Highly adaptable element types and a wide range of alloys.<br>3. Special equipment required. | *Nat Synth* 1, 138–146 (2022). |

## Supplementary References


1 S. Werner, F. Arthur and B. Ernst, *J. Colloid Interface Sci.*, 1968, **26**, 62–69.
2 G. Kresse and J. Furthmüller, *Comput. Mater. Sci.*, 1996, **6**, 15–50.
3 G. Kresse and J. Furthmüller, *Phys. Rev. B*, 1996, **54**, 11169–11186.
4 G. Kresse and J. Hafner, *Phys. Rev. B*, 1993, **47**, 558–561.
5 J. P. Perdew, K. Burke and M. Ernzerhof, *Phys. Rev. Lett.*, 1996, **77**, 3865–3868.
6 G. Kresse and D. Joubert, *Phys. Rev. B - Condens. Matter Mater. Phys.*, 1999, **59**, 1758–1775.
7 P. E. Blöchl, *Phys. Rev. B*, 1994, **50**, 17953–17979.
8 S. Grimme, J. Antony, S. Ehrlich and H. Krieg, *J. Chem. Phys.*, 2010, **132**, 154104.
9 H. J. Monkhorst and J. D. Pack, *Phys. Rev. B*, 1976, **13**, 5188–5192.
10 S. L. Dudarev, G. A. Botton, S. Y. Savrasov, C. J. Humphreys and A. P. Sutton, *Phys. Rev. B*, 1998, **57**, 1505–1509.
11 L. I. Bendavid and E. A. Carter, *J. Phys. Chem. C*, 2013, **117**, 26048–26059.
12 J. K. Nørskov, T. Bligaard, A. Logadottir, J. R. Kitchin, J. G. Chen, S. Pandelov and U. Stimming, *J. Electrochem. Soc.*, 2005, **152**, J23–J26.
13 W. Sheng, M. Myint, J. G. Chen and Y. Yan, *Energy Environ. Sci.*, 2013, **6**, 1509–1512.
14 H. A. Hansen, V. Viswanathan and J. K. Nørskov, *J. Phys. Chem. C*, 2014, **118**, 6706–6718.
15 C. G. Morales-Guio, L. A. Stern and X. Hu, *Chem. Soc. Rev.*, 2014, **43**, 6555–6569.
16 G. Feng, F. Ning, J. Song, H. Shang, K. Zhang, Z. Ding, P. Gao, W. Chu and D. Xia, *J. Am. Chem. Soc.*, 2021, **143**, 17117–17127.
17 H. J. Niu, H. Y. Chen, G. L. Wen, J. J. Feng, Q. L. Zhang and A. J. Wang, *J. Colloid Interface Sci.*, 2019, **539**, 525–532.
18 H. Jin, Z. Xu, Z. Hu, Z. Yin, Z. Wang, Z. Deng, P. Wei, S. Feng, S. Dong, J. Liu, S. Luo, Z. Qiu, L. Zhou, L. Mai, B. Su, D. Zhao and Y. Liu, *Nat. Commun.*, 2023, **14**, 15118.
19 Z. Li, R. Yu, J. Huang, Y. Shi, D. Zhang, X. Zhong, D. Wang, Y. Wu and Y. Li, *Nat. Commun.*, 2015, **6**, 8248.
20 C. Li, X. Chen, L. Zhang, S. Yan, A. Sharma, B. Zhao, A. Kumbhar, G. Zhou and J. Fang, *Angew. Chem. Int. Ed.*, 2021, **60**, 7675–7680.
21 H. Liu, K. Liu, P. Zhong, J. Qi, J. Bian, Q. Fan, K. Ren, H. Zheng, L. Han, Y. Yin and C. Gao, *Chem. Mater.*, 2018, **30**, 7744–7751.
22 S. Xue, W. Deng, F. Yang, J. Yang, I. S. Amiinu, D. He, H. Tang and S. Mu, *ACS Catal.*, 2018, **8**, 7578–7584.
23 W. Wang, X. Chen, X. Zhang, J. Ye, F. Xue, C. Zhen, X. Liao, H. Li, P. Li, M. Liu, Q. Kuang, Z. Xie and S. Xie, *Nano Energy*, 2020, **71**, 104623.
24 Q. Wang, S. Chen, H. Lan, P. Li, X. Ping, S. Ibraheem, D. Long, Y. Duan and Z. Wei, *J. Mater. Chem. A*, 2019, **7**, 18143–18149.
25 J. Zhang, X. Qu, Y. Han, L. Shen, S. Yin, G. Li, Y. Jiang and S. Sun, *Appl. Catal. B Environ.*, 2020, **263**, 118345.
26 L. Huang, X. Zhang, Y. Han, Q. Wang, Y. Fang and S. Dong, *Chem. Mater.*, 2017, **29**, 4557–4562.
27 G. Bai, C. Liu, Z. Gao, B. Lu, X. Tong, X. Guo and N. Yang, *Small*, 2019, **15**, 1902951.
28 Q. Feng, S. Zhao, D. He, S. Tian, L. Gu, X. Wen, C. Chen, Q. Peng, D. Wang and Y. Li, *J. Am. Chem. Soc.*, 2018, **140**, 2773–2776.
29 P. Yang, X. Yuan, H. Hu, Y. Liu, H. Zheng, D. Yang, L. Chen, M. Cao, Y. Xu, Y. Min, Y. Li and Q. Zhang, *Adv. Funct. Mater.*, 2018, **28**, 1704774.
30 L. Tao, Y. Shi, Y. C. Huang, R. Chen, Y. Zhang, J. Huo, Y. Zou, G. Yu, J. Luo, C. L. Dong and S. Wang, *Nano Energy*, 2018, **53**, 604–612.
31 C. Zhang, Y. Dai, H. Chen, Y. Ma, B. Jing, Z. Cai, Y. Duan, B. Tang and J. Zou, *J. Mater. Chem. A*, 2018, **6**, 22636–22644.
32 H. H. Li, Q. Q. Fu, L. Xu, S. Y. Ma, Y. R. Zheng, X. J. Liu and S. H. Yu, *Energy Environ. Sci.*, 2017, **10**, 1751–1756.
33 L. Yang, G. Li, R. Ma, S. Hou, J. Chang, M. Ruan, W. Cai, Z. Jin, W. Xu, G. Wang, J. Ge, C. Liu and W. Xing, *Nano Res.*, 2021, **14**, 2853–2860.
34 Z. Li, X. Jiang, X. Wang, J. Hu, Y. Liu, G. Fu and Y. Tang, *Appl. Catal. B Environ.*, 2020, **277**, 119135.
35 W. Y. Zhao, B. Ni, Q. Yuan, P. L. He, Y. Gong, L. Gu and X. Wang, *Adv. Energy Mater.*, 2017, **7**, 1601593.
36 T. Zhang, Y. Sun, X. Li, X. Li, D. Liu, G. Liu, C. Li, H. J. Fan and Y. Li, *Small Methods*, 2020, **4**, 1900709.

37 L. Huang, X. Zhang, Q. Wang, Y. Han, Y. Fang and S. Dong, *J. Am. Chem. Soc.*, 2018, **140**, 1142–1147.
38 W. Zhang, Y. Yang, B. Huang, F. Lv, K. Wang, N. Li, M. Luo, Y. Chao, Y. Li, Y. Sun, Z. Xu, Y. Qin, W. Yang, J. Zhou, Y. Du, D. Su and S. Guo, *Adv. Mater.*, 2019, **31**, 1805833.
39 L. Huang, M. Wei, N. Hu, P. Tsiakaras and P. Kang Shen, *Appl. Catal. B Environ.*, 2019, **258**, 117974.
40 Z. Zhang, Y. Chen, L. Zhou, C. Chen, Z. Han, B. Zhang, Q. Wu, L. Yang, L. Du, Y. Bu, P. Wang, X. Wang, H. Yang and Z. Hu, *Nat. Commun.*, 2019, **10**, 1657.
41 B. Jiang, Z. Tang, F. Liao, H. Lin, S. Lu, Y. Li and M. Shao, *J. Mater. Chem. A*, 2017, **5**, 21903–21908.
42 J. Zhang, Y. Zhao, X. Guo, C. Chen, C. L. Dong, R. S. Liu, C. P. Han, Y. Li, Y. Gogotsi and G. Wang, *Nat. Catal.*, 2018, **1**, 985–992.
43 N. Cheng, S. Stambula, D. Wang, M. N. Banis, J. Liu, A. Riese, B. Xiao, R. Li, T. K. Sham, L. M. Liu, G. A. Botton and X. Sun, *Nat. Commun.*, 2016, **7**, 13638.
44 J. Ying, G. Jiang, Z. Paul Cano, L. Han, X. Y. Yang and Z. Chen, *Nano Energy*, 2017, **40**, 88–94.
45 J. Yang, W. H. Li, S. Tan, K. Xu, Y. Wang, D. Wang and Y. Li, *Angew. Chem. Int. Ed.*, 2021, **60**, 19085–19091.
46 D. Liu, X. Li, S. Chen, H. Yan, C. Wang, C. Wu, Y. A. Haleem, S. Duan, J. Lu, B. Ge, P. M. Ajayan, Y. Luo, J. Jiang and L. Song, *Nat. Energy*, 2019, **4**, 512–518.
47 Y. L. Wu, X. Li, Y. S. Wei, Z. Fu, W. Wei, X. T. Wu, Q. L. Zhu and Q. Xu, *Adv. Mater.*, 2021, **33**, 2006965.
48 X. Huang, Z. Zeng, S. Bao, M. Wang, X. Qi, Z. Fan and H. Zhang, *Nat. Commun.*, 2013, **4**, 1444.
49 M. Tavakkoli, N. Holmberg, R. Kronberg, H. Jiang, J. Sainio, E. I. Kauppinen, T. Kallio and K. Laasonen, *ACS Catal.*, 2017, **7**, 3121–3130.
50 H. Zhang, P. An, W. Zhou, B. Y. Guan, P. Zhang, J. Dong and X. W. Lou, *Sci. Adv.*, 2018, **4**, 6657.
51 J. Chen, G. Qian, H. Zhang, S. Feng, Y. Mo, L. Luo and S. Yin, *Adv. Funct. Mater.*, 2022, **32**, 2107597.
52 X. Wu, W. Xu, Z. Wang, H. Li, M. Wang, D. Zhang, J. Lai and L. Wang, *Chem. Eng. J.*, 2022, **431**, 133247.
53 A. Shan, X. Teng, Y. Zhang, P. Zhang, Y. Xu, C. Liu, H. Li, H. Ye and R. Wang, *Nano Energy*, 2022, **94**, 106913.
54 J. Zhang, E. Wang, S. Cui, S. Yang, X. Zou and Y. Gong, *Nano Lett.*, 2022, **22**, 1398–1405.
55 Y. Liu, N. Chen, W. Li, M. Sun, T. Wu, B. Huang, X. Yong, Q. Zhang, L. Gu, H. Song, R. Bauer, J. S. Tse, S. Zang, B. Yang and S. Lu, *SmartMat*, 2021, **3**, 249–259.
56 B. Pang, X. Liu, T. Liu, T. Chen, X. Shen, W. Zhang, S. Wang, T. Liu, D. Liu, T. Ding, Z. Liao, Y. Li, C. Liang and T. Yao, *Energy Environ. Sci.*, 2022, **15**, 102–108.
57 D. Wu, K. Kusada, Y. Nanba, M. Koyama, T. Yamamoto, T. Toriyama, S. Matsumura, O. Seo, I. Gueye, J. Kim, L. S. Rosantha Kumara, O. Sakata, S. Kawaguchi, Y. Kubota and H. Kitagawa, *J. Am. Chem. Soc.*, 2022, **144**, 3365–3369.
